# Aqueous Extract of *Syringa oblata* Lindl. Alleviates Murine Endometritis by Modulating TLR4/MyD88 Signaling and Macrophage Polarization

**DOI:** 10.3390/vetsci13060526

**Published:** 2026-05-28

**Authors:** Yang Zhang, Jinjin Shen, Jiawen Li, Tong Zhu, Jiahao Fu, Xueying Chen, Jing Su, Jingyou Hao, Yanhua Li, Yanyan Liu

**Affiliations:** 1 College of Veterinary Medicine, Northeast Agricultural University, Harbin 150030, China; s240602146@neau.edu.cn (Y.Z.); sxj13435594349@163.com (J.S.); 15063390190@163.com (J.L.); 15561885429@163.com (T.Z.); 1583512857a@gmail.com (J.F.); chenxueying@neau.edu.cn (X.C.); liyanhua@neau.edu.cn (Y.L.); 2 Heilongjiang Key Laboratory for Animal Disease Control and Pharmaceutical Development, Harbin 150030, China; 3 Heilongjiang Provincial Center for Animal Disease Prevention and Control, No. 243, Haping Road, Xiangfang District, Harbin 150000, China; 13804539246@139.com; 4 Harbin Lvda Biotechnology Co., Ltd., No. 77, Chenggaozi Section, Jiangnan Middle Ring Road, Xiangfang District, Harbin 150000, China; shj.228@163.com

**Keywords:** *Syringa oblata* Lindl., endometritis, TLR4/MyD88 signaling, macrophage polarization, flavonoids

## Abstract

*Syringa oblata* Lindl. (SOL) has long been used in traditional medicine for inflammatory disorders, yet its molecular actions in reproductive tract inflammation remain poorly defined. This study investigated the phytochemical composition and anti-inflammatory mechanisms of an aqueous SOL leaf extract using murine and cellular models of endometritis. Ultra-performance liquid chromatography–tandem mass spectrometry (UPLC–MS/MS) analysis revealed major constituents including rutin, salidroside, and esculetin. In a murine model of bacterial endometritis induced by *Escherichia coli* and *Staphylococcus aureus*, SOL markedly attenuated uterine edema, epithelial disruption, leukocyte infiltration, and bacterial burden. Mechanistic analyses demonstrated that SOL suppressed the Toll-like receptor 4 (TLR4)/myeloid differentiation primary response 88 (MyD88) axis and decreased the uterine expression of interleukin-1 beta (IL-1β), interleukin-6 (IL-6), and tumor necrosis factor-alpha (TNF-α). In lipopolysaccharide (LPS)-stimulated RAW264.7 macrophages, SOL and its principal monomers significantly reduced nitric oxide (NO) and reactive oxygen species (ROS) production both in the presence and absence of the TLR4 inhibitor TAK-242, suggesting additional modulation of redox-responsive pathways beyond canonical TLR4 signaling. Moreover, SOL selectively decreased the M1 macrophage marker CD86 in uterine tissue without altering CD163, consistent with partial inhibition of pro-inflammatory macrophage polarization. Collectively, these findings indicate that SOL exerts potent antimicrobial, anti-inflammatory, and antioxidative effects through coordinated regulation of innate immune signaling and macrophage activation, supporting its potential as a natural therapeutic candidate for inflammation-associated reproductive disorders.

## Simple Summary

Uterine infections in livestock, such as dairy cows and pigs, cause poor reproductive health and significant economic losses. These bacterial infections are usually treated with antibiotics, but the rising global threat of antibiotic resistance makes it urgent to find safe, natural alternatives. This study aimed to investigate whether a water-based extract from the leaves of *Syringa oblata* Lindl., which has a long history in traditional medicine, could effectively treat these infections. By testing this extract on mice with bacterial uterine infections and on immune cells in the laboratory, we found that the treatment significantly reduced uterine swelling, tissue damage, and the number of harmful bacteria. The plant extract works by blocking specific biological signals that cause excessive inflammation and by calming overactive immune cells. We conclude that this lilac leaf extract has strong potential to be developed into an effective, plant-derived medicine for animal reproductive tract infections. This discovery is valuable to society because it offers a natural way to improve livestock health while helping to reduce the farming industry’s reliance on conventional antibiotics.

## 1. Introduction

Endometritis is increasingly recognised not only in human reproductive medicine but also in livestock production, where it contributes to infertility, implantation failure, and reproductive inefficiency. In women, chronic endometritis has been shown to be significantly more prevalent among those experiencing infertility and recurrent pregnancy loss, underscoring its impact on reproductive outcomes [1,2]. In the livestock sector, especially in dairy cows and sows, postpartum endometritis remains one of the most common uterine disorders, with uterine microbial contamination undermining endometrial repair, disrupting estrous cycles and extending calving-to-conception intervals, thereby imposing substantial economic burdens on production systems [3]. Given the rising global demand for animal-derived food products and stricter regulations on antimicrobial residues, the development of safe, effective and sustainable alternatives to conventional antibiotics for uterine infection becomes a key priority in both veterinary and public health contexts.

Natural compounds with dual antimicrobial and host-directed immunomodulatory activities have recently attracted growing attention as alternatives to conventional drugs [4]. *Syringa oblata* Lindl. (SOL) has long been used in traditional folk medicine across Eurasia and northern China to address a range of ailments, such as respiratory and inflammatory disorders. Notably, it is rich in flavonoids, phenylethanoid glycosides, and lignans, which are phytochemical classes widely recognized for their anti-inflammatory and antioxidant properties [5]. Based on our previous findings [6], *S. oblata* leaf extract markedly alleviated murine endometritis induced by *Staphylococcus aureus* by reducing uterine inflammation and bacterial load, confirming its pharmacological potential. Building on this foundation, the present work aimed to elucidate the chemical constituents of *S. oblata* aqueous extract and dissect the mechanistic basis of its therapeutic effects. Modern phytochemical studies have confirmed the presence of bioactive components such as luteolin, rutin, and salidroside in *S. oblata* leaves, which exhibit in vitro antimicrobial activity against *S. aureus* and *E. coli* and inhibit inflammatory signaling in macrophages [7,8]. These findings suggest that *S. oblata* may provide a multi-target therapeutic strategy for reproductive tract infections. However, its mechanistic role in bacterial endometritis remains largely unexplored.

Bacterial endometritis pathogenesis is strongly associated with an excessive inflammatory response in the host, triggered by pathogen-associated molecular patterns (PAMPs). During and after parturition, opportunistic pathogens such as *E. coli* and *Staphylococcus* spp. can ascend into the uterine cavity, adhering to and damaging the endometrial epithelium [9,10]. This triggers innate immune recognition through pattern recognition receptors (PRRs), notably Toll-like receptors (TLRs). Among them, TLR4 has been identified as a central mediator in the recognition of lipopolysaccharide (LPS) and other pathogen-associated molecular patterns. Activation of TLR4 leads to the recruitment of the adaptor protein MyD88 and subsequent activation of NF-κB and MAPK signaling cascades, resulting in the transcriptional upregulation of pro-inflammatory cytokines, such as TNF-α, IL-1β, and IL-6 [11,12]. Furthermore, the dysregulation of macrophage polarization, particularly the excessive shift towards a pro-inflammatory M1 phenotype, is a critical factor in the amplification and perpetuation of uterine tissue damage. M1-polarized macrophages secrete pro-inflammatory mediators that exacerbate tissue injury, while M2 macrophages promote resolution and tissue remodeling [13]. In models of chronic uterine inflammation, a skewed balance toward M1 polarization correlates with fibrotic remodeling and impaired regenerative capacity [14]. Therefore, therapeutic strategies that both attenuate TLR4/MyD88 signaling and re-balance macrophage polarization represent promising approaches for the management of endometritis.

In this study, we investigated the anti-inflammatory and antimicrobial potential of an aqueous *S. oblata* leaf extract using UPLC-MS/MS-based chemical profiling and both in vivo and in vitro models of bacterial endometritis. Our objective was to determine if the *S. oblata* aqueous extract reduces uterine inflammation by inhibiting the TLR4/MyD88 pathway and altering macrophage polarization. This research offers insights into its potential as a plant-based therapeutic for managing livestock reproductive health.

## 2. Materials and Methods

### 2.1. Preparation of Aqueous SOL Extract

*Syringa oblata* Lindl. was harvested from the Lilac Plantation of Northeast Agricultural University from September to October. After cleaning, the raw materials were naturally air-dried in the shade at room temperature. Once dried and crushed, SOL was sieved through a No. 5 sieve to obtain a crude powder, which was then sealed and stored under room temperature conditions. Before extraction, the crude powder was soaked in advance for 3 h. The initial extraction used 20 times the volume of deionized water, followed by a 45 min simmer to produce the first decoction. A second extraction was then conducted with 15 times the volume of deionized water, simmered for 30 min. The resulting second decoction was filtered and combined with the first extract, after which the combined solution was rapidly concentrated under reduced pressure to one-tenth of its original volume, with the temperature maintained below 60 °C.

### 2.2. UPLC-MS/MS Analysis of SOL

The aqueous extract of *Syringa oblata* Lindl. (SOL) was analyzed using ultra-performance liquid chromatography–tandem mass spectrometry (UPLC–MS/MS) to characterize its major chemical constituents. The analysis was performed by Wuhan Metware Biotechnology Co., Ltd. (Wuhan, China). The liquid chromatography conditions were as follows: chromatographic separation was performed on an SB-C18 column (Agilent, Santa Clara, CA, USA; 2.1 × 100 mm, 1.8 µm) at a column temperature of 40 °C, with an injection volume of 2 µL and a flow rate of 0.35 mL/min. The mobile phase was composed of 0.1% formic acid in water (A) and acetonitrile (B). The elution gradient was programmed as follows: initially 95–5% A for 0–9 min, then maintained at 5% A for 9–10 min, increased to 95% A for 10–11.1 min, and finally held at 95% A for 11.1–14 min. For mass spectrometry, electrospray ionization (ESI) was operated in both positive and negative ion modes, with spray voltages set at +5500 V and −4500 V, respectively. The ion source gases were set as follows: gas I at 50 psi, gas II at 60 psi, and curtain gas at 25 psi. The ion source temperature was maintained at 500 °C. The collision-induced dissociation (CID) parameter was adjusted to high, with nitrogen as the collision gas at medium intensity. The triple quadrupole (QQQ) mass analyzer functioned in multiple reaction monitoring (MRM) mode. For each MRM transition, the declustering potential (DP) and collision energy (CE) were optimized. Monitoring specific MRM transitions across various elution time windows facilitated efficient metabolite detection.

We conducted qualitative analysis of the compounds using the MWDB database and tandem mass spectrometry data. The mass spectrometry data were processed and analyzed with Analyst 1.6.3 software.

### 2.3. Building the SOL Components-Key Targets-Endometritis Network

The targets associated with the active components of SOL were retrieved using the TCMSP database, while disease-related targets linked to endometritis were identified through the DrugBank and UniProt databases. *Sus scrofa* was selected as the target species to standardize the gene nomenclature of the identified targets. The intersection of disease targets and component-related targets was analyzed to identify the key therapeutic targets of *Syringa oblata* Lindl. extract. Based on the interactions between active components and potential key targets, an association network linking SOL components, key targets, and endometritis was constructed using Cytoscape 3.10.1 software. Topological analysis of the constructed network was performed using the CytoNCA plugin 2.1.6, with a focus on the key topological parameter—Degree. The Degree value represents the number of connections between nodes; a higher Degree indicates more connections and suggests a more central role of the node within the network, implying greater functional importance in the underlying therapeutic mechanism.

### 2.4. Quantitative Analysis of the Main Active Components in the SOL Extract

Based on the degree values of network pharmacology, the top three active components—rutin, salidroside, and esculetin—were selected. The contents of these components in the SOL were determined using high-performance liquid chromatography (HPLC). Rutin standard solutions with concentrations ranging from 50 to 500 µg/mL, salidroside standard solutions within the same concentration range, and esculetin standard solutions ranging from 5 to 100 µg/mL were prepared using methanol. HPLC analysis was performed using a C18 reversed-phase chromatographic column (Dikema, Beijing, China; 4.6 × 250 mm, 5 µm) at a column temperature of 25 °C. Each concentration was measured in triplicate, and the detailed HPLC conditions are summarized in Table 1.

### 2.5. Experimental Procedures Involving Animals

#### 2.5.1. Animal Model and Drug Administration

Female Kunming mice (6–8 weeks old and weighing 30–35 g) were purchased from Liaoning Changsheng Biotechnology Co., Ltd. (Benxi, China)All experimental procedures were conducted in accordance with the approved protocol (No. NEAUEC202303134) of the Animal Welfare and Ethics Committee of Northeast Agricultural University. After a one-week acclimatization period, the mice were randomly divided into the following eight groups (*n* = 7): Control Group (CG), Model Group (MG), *Syringa oblata* Control Group (CSOG), High-dose *Syringa oblata* Group (HSOG, 500 mg/mL), Medium-dose *Syringa oblata* Group (MSOG, 375 mg/mL), Low-dose *Syringa oblata* Group (LSOG, 250 mg/mL), Dexamethasone positive control group (DEXG, 5 mg/kg), and Yimu Shenghua positive traditional Chinese medicine control group (YSHG, 1.14 g/mL). The detailed daily intrauterine administration schedules for each group are visually summarized in Table 2. The dose selection and the specialized route of administration were established according to the validated protocols previously published by our laboratory [6], supplemented by preliminary dose-finding screening to ensure optimal therapeutic efficacy without inducing systemic toxicity. The experimental protocol spanned a six-day intervention period, divided into a three-day infection phase and a subsequent three-day treatment phase.

To establish the bacterial endometritis model, mice in the MG and all five treatment groups (HSOG, MSOG, LSOG, DEXG, and YSHG) received daily intrauterine infusions of a mixed bacterial suspension for three consecutive days. This suspension contained *Escherichia coli* and *Staphylococcus aureus*, each adjusted to a concentration of 1 × 10^9^ CFU/mL. The infusion was performed using a specialized dental side-vented needle to ensure even intra-uterine distribution. Immediately following each infusion, the mice were maintained in an inverted position for one minute to promote localized retention of the pathogens and prevent premature expulsion. Starting 24 h after the final bacterial challenge, the respective therapeutic formulations in a volume of 100 μL were administered via daily intrauterine infusion for three consecutive days. Concurrently, to control for any confounding artifacts arising from handling-induced stress, mice in the CG received 100 μL of normal saline daily throughout the entire six-day period. Mice in the MG received equal volumes of normal saline during the treatment phase, while the CSOG received normal saline during the infection phase, followed by 500 mg/mL SOL extract during the treatment phase. All mice were euthanized 24 h after the final administration, and uterine tissues were harvested for subsequent analysis.

#### 2.5.2. H&E Staining

Prior to histological processing, the uterine index was calculated for each mouse to evaluate macroscopic organ swelling. Subsequently, the excised uterine tissues were fixed in 4% paraformaldehyde solution for 24 h. The tissues were then processed for paraffin embedding, sectioned into thin slices, and stained with hematoxylin and eosin (H&E). Histopathological evaluation was performed by examining the stained sections under an optical microscope at 200× magnification. To quantitatively assess the severity of uterine pathology, a semi-quantitative histopathological scoring system (graded from 0 to 5) was employed. The sections were evaluated in a blinded manner based on the extent of tissue injury, interstitial edema, mucosal epithelial shedding, and inflammatory cell infiltration.

#### 2.5.3. Determination of Bacterial Load in the Uterine Tissues of Mice

The uterine tissues from each group were accurately weighed, and 1 mL of pre-cooled sterile PBS was used to homogenize the tissues. Subsequently, the tissue homogenates were serially diluted with sterile PBS, and aliquots of each dilution were inoculated onto LB solid medium. Following incubation at 37 °C for 16 to 20 h, the colonies were enumerated, and the total counts of *E. coli* and *S. aureus* were determined. The bacterial load was then calculated and expressed as colony-forming units per gram of tissue (CFUs/g tissue, log10).

#### 2.5.4. Uterine Tissues Immunofluorescence

Immunofluorescence staining was performed on the uterine tissues of mice. Following slide mounting, images were captured using a fluorescence microscope. Cell nuclei were stained blue (DAPI, excitation wavelength 330–380 nm, emission wavelength 420 nm); CD68 expression was visualized in red (CY3, excitation wavelength 510–560 nm, emission wavelength 590 nm); and NF-κB expression was detected in green (488, excitation wavelength 465–495 nm, emission wavelength 515–555 nm). Image J software was employed to analyze the fluorescence images, and the expression levels of CD68 and NF-κB proteins were quantified based on the corresponding fluorescence intensities.

### 2.6. Cellular Experimentation

#### 2.6.1. Cell Culture and Viability Assay

Murine macrophage RAW264.7 cells were obtained from the Shanghai Preservation Biotechnology Center (Shanghai, China). Cells were cultured in Dulbecco’s modified Eagle’s medium (DMEM) supplemented with 12.5% fetal bovine serum (FBS) at 37 °C in a humidified atmosphere containing 5% CO_2_. For all experiments, cells were seeded in appropriate culture plates and divided into the following groups: control (untreated), model (LPS), SOL-treated (0.625, 1.25, 2.5 mg/mL), monomer-treated (rutin 8.85 μg/mL, salidroside 6.64 μg/mL, esculetin 0.087 μg/mL), monomer combination and inhibitor co-treatment (TAK-242 + SOL/monomer). The in vitro concentrations of the SOL extract were strictly determined based on the maximal non-cytotoxic concentration. For the mixed monomers group, the three monomers were mixed at their exact individual final concentrations to preserve their natural mass ratios present in the 2.5 mg/mL SOL extract. The commercial standards of rutin, salidroside, and esculetin (all with purity ≥ 98%) were purchased from Shanghai Yuanye Bio-Technology Co., Ltd. (Shanghai, China).

#### 2.6.2. Cell Viability Assay

The cytotoxicity of SOL was assessed using the Cell Counting Kit-8 (CCK-8, Meilunbio, Dalian, China). RAW264.7 cells were seeded in 96-well plates at a density of 1 × 10^4^ cells/well and allowed to adhere for 12 h. Cells were then treated with SOL at concentrations of 0.625, 1.25, 2.5, 5, 10, 20, 40, 60, and 80 mg/mL for 24 h. Subsequently, 10 μL CCK-8 reagent was added to each well and incubated for 1 h at 37 °C. Absorbance was measured at 450 nm using a microplate reader (SpectraMax M5, Molecular Devices, San Jose, CA, USA).

#### 2.6.3. Determination of NO Production

The concentration of nitric oxide (NO) in the cell culture supernatants was measured using a commercial NO assay kit (Beyotime, Shanghai, China) based on the Griess reaction. RAW264.7 cells were seeded in 96-well plates and treated as described above. After 24 h incubation, culture supernatants were collected and mixed with Griess reagents I and II. Absorbance was recorded at 540 nm, and NO levels were calculated from a standard curve of sodium nitrite.

#### 2.6.4. Measurement of Cellular ROS

The intracellular reactive oxygen species (ROS) production in RAW264.7 macrophages was determined using a DCFH-DA fluorescent probe (Solarbio, Beijing, China) according to the manufacturer’s protocol. Briefly, cells were seeded in 96-well plates at a density of 1 × 10^4^ cells per well, with six parallel wells assigned to each treatment group. After adherence, cells were stimulated with 1 µg/mL LPS for 2 h to induce oxidative stress, followed by treatment with SOL or the indicated reference compounds for 24 h. Subsequently, the cells were incubated with 10 µM DCFH-DA working solution at 37 °C for 40 min in the dark to allow intracellular oxidation of the probe. After incubation, the cells were washed three times with PBS to remove excess dye, and the fluorescence intensity was measured using a multimode microplate reader (excitation at 485 nm, emission at 530 nm). The relative fluorescence intensity was used as an indicator of intracellular ROS levels.

#### 2.6.5. Morphological Observation and Macrophage Polarization

Macrophage M1 polarization was induced by stimulating cells with LPS (1 μg/mL) for 24 h. For inhibitor experiments, cells were pretreated with the TLR4 inhibitor TAK-242 (Resatorvid, 1 μM) for 1 h prior to addition of SOL or monomers. After treatment, the culture medium was aspirated, cells were gently washed three times with sterile PBS, and fixed with 4% paraformaldehyde in PBS for 30 min at room temperature. Fixed cells were rinsed twice with PBS and stained with hematoxylin for 3 min to enhance cytomorphological contrast; excess dye was removed by rinsing with distilled water. Morphological features were examined using an inverted fluorescence microscope and imaged at multiple fields per well. Morphological criteria were used to infer activation state: cells exhibiting flattened or spread morphologies with extended pseudopodia were considered consistent with M1-type activation, whereas rounded or less-extended cells were interpreted as less activated or shifted toward a non-inflammatory phenotype.

### 2.7. Quantitative Real-Time Polymerase Chain Reaction

Total RNA was extracted from uterine tissues and cells following the manufacturer’s instructions for the RNA extraction kit. The nucleic acid concentration of the isolated RNA was determined, and the RNA was subsequently reverse-transcribed into complementary DNA (cDNA) using a reverse transcription kit, ensuring uniform concentration across samples. The synthesized cDNA was stored at −20 °C for subsequent analysis. The mRNA expression levels of TLR4, MyD88, CD86, TNF-α, IL-1β, and IL-6, relative to the internal reference gene β-actin, were quantified using the 2^−ΔΔCt^ method. The qPCR primers were designed and synthesized by Saiwen Innovation (Harbin, China) Biotechnology Co., Ltd., and the corresponding primer sequences are detailed in Table 3.

### 2.8. Western Blot Analysis

Mouse uterine tissues were ground into a fine powder with liquid nitrogen and lysed in RIPA buffer containing PMSF and phosphatase inhibitor (100:1:1) for 30 min on ice. The lysates were centrifuged at 12,000 rpm for 10 min at 4 °C, and the supernatant was collected for protein quantification. Equal amounts of protein were separated via SDS-PAGE, transferred onto PVDF membranes, and blocked with 5% skim milk for 2 h at room temperature. Membranes were incubated overnight at 4 °C with primary antibodies against TLR4 (1:2000), MyD88 (1:500), CD86 (1:2000), CD163 (1:2000), β-actin (1:5000), IL-6 (1:2000), IL-1β (1:1000), TNF-α (1:2000), and NF-κB (1:1000), followed by HRP-conjugated secondary antibodies (1:15,000) for 1 h. Protein bands were visualized using a chemiluminescence imaging system and quantified with ImageJ (version 1.53).

### 2.9. Data Analysis

Statistical analysis was performed using GraphPad Prism 8. All data are presented as mean ± standard deviation ($\bar{x}$ ± SD). For comparison among multiple groups, one-way analysis of variance (ANOVA) was applied, followed by Tukey’s post hoc test for multiple comparisons. A *p*-value less than 0.05 was considered statistically significant.

## 3. Results

### 3.1. UPLC-MS/MS and Network Pharmacology Analysis of SOL Extract

**Figure 1:**
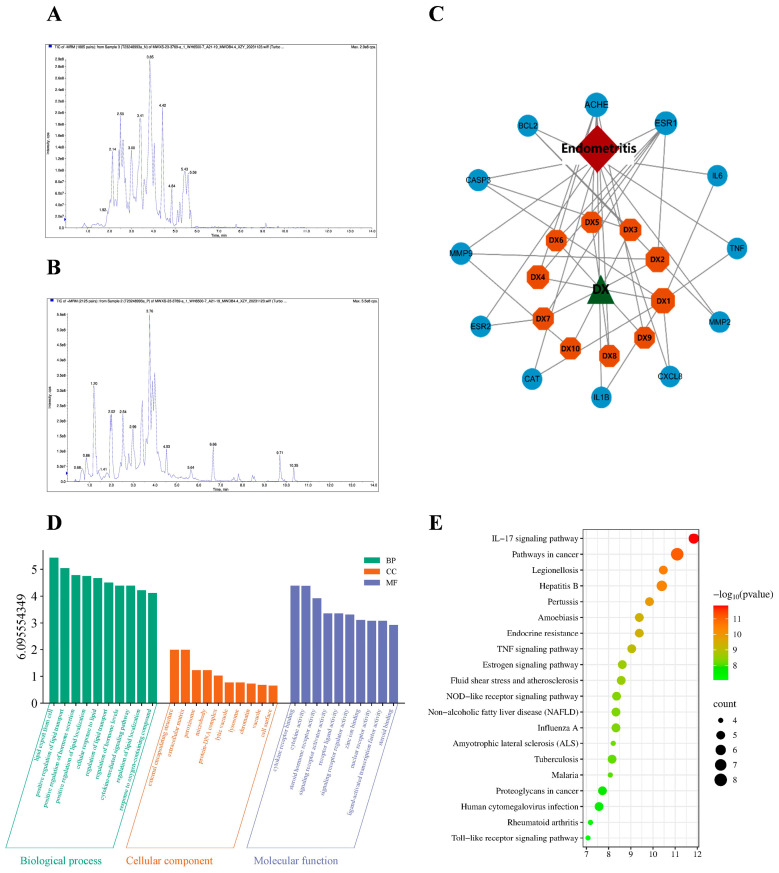
UPLC-MS/MS analysis of the SOL aqueous extract. Total Ion Chromatograms of the extract in negative ion mode (**A**) and positive ion mode (**B**). The full list of identified metabolites is available in Supplementary Table S1. (**C**) Establishment of SOL Components–key targets–endometritis Network. In the network, the green triangle represents the SOL extract (DX), the red diamond represents the disease (endometritis), orange hexagons represent the active components, and blue circles represent the core therapeutic targets. (**D**) GO enrichment analysis. (**E**) KEGG enrichment analysis.

Representative Total Ion Chromatograms (TIC) of the SOL aqueous extract in negative and positive ion modes are shown in Figure 1A,B. By matching the tandem mass spectrometry data with the MWDB database in MRM mode, a comprehensive chemical profile was obtained, identifying a total of 392 (negative ion mode) and 478 (positive ion mode) chemical substances. The complete list of all identified metabolites is provided in Supplementary Table S1. Subsequently, an association network linking SOL components, key targets, and endometritis was constructed, which revealed ten core compounds associated with the disease, as summarized in Table 4. These compounds include Rutin (DX1), Salidroside (DX2), Esculetin (DX3), Secoisolariciresinol (DX4), Dihydrocubebin (DX5), Dehydrodiconiferyl alcohol (DX6), 2,4-Dihydroxy-6-methoxyacetophenone (DX7), Embelin (DX8), Salvianic Acid A (DX9), and Vanillin (DX10). The simultaneous interactions between various disease targets and components suggest that the SOL extract exerts its therapeutic effects on endometritis through a synergistic, multi-target mechanism (Figure 1C). To further characterize the functional mechanisms underlying this multi-target network, Gene Ontology (GO) and Kyoto Encyclopedia of Genes and Genomes (KEGG) enrichment analyses were conducted on the intersecting targets. The GO biological process (BP) analysis (Figure 1D) indicated that the core targets were primarily involved in lipid localization and transport, cellular response to oxygen-containing compounds, and the cytokine-mediated signaling pathway. Concurrently, KEGG pathway enrichment analysis (Figure 1E) revealed that these targets were highly correlated with an interconnected network of classical inflammatory and immune cascades, including the IL-17 signaling pathway, TNF signaling pathway, NOD-like receptor signaling pathway, and Toll-like receptor signaling pathway. Although multiple downstream effector cascades showed strong enrichment, the Toll-like receptor signaling pathway represents the pivotal upstream pattern recognition axis driving bacterial recognition and initial cytokine mobilization. Therefore, these bioinformatics findings provided a direct theoretical basis for our subsequent experimental validation of the TLR4/MyD88 signaling pathway.

### 3.2. Content Determination of the Main Active Ingredients in SOL Extract

**Figure 2:**
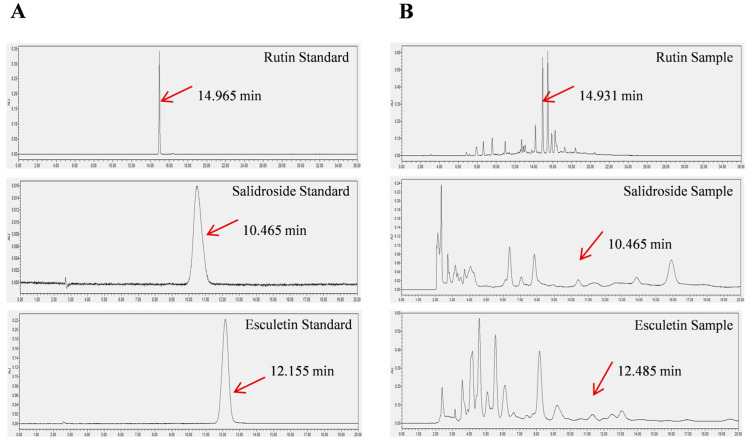
HPLC chromatograms of standards (**A**) and SOL sample (**B**).

The contents of the main effective components (rutin, salidroside, and esculetin) in the SOL were determined using HPLC. The retention times of the standard samples of rutin, salidroside, and esculetin were 14.965 min, 10.465 min, and 12.155 min, respectively, which corresponded to those of the corresponding components in the extract (Figure 2A,B). Additionally, the results indicated that rutin exhibited the highest content in the SOL. The standard curves and corresponding content data of the main active components in the extract are presented in the Supplementary Materials.

### 3.3. The Histopathological Effects of SOL Extract

**Figure 3:**
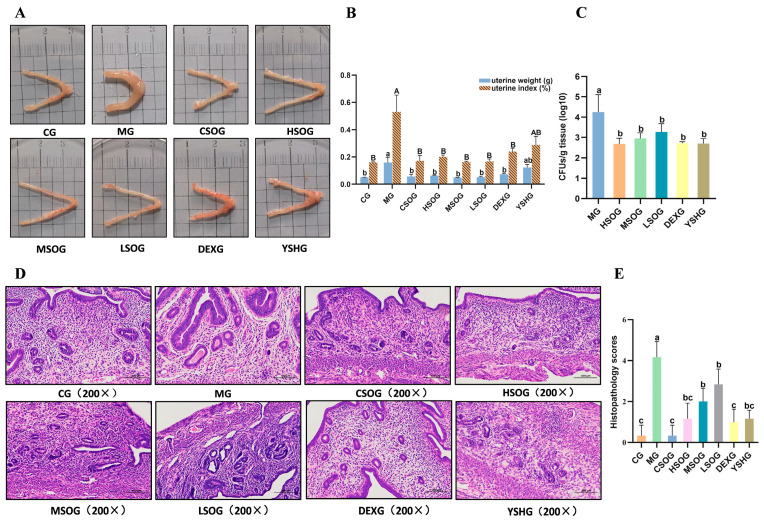
Effects of SOL on the uterine histopathology of mice with endometritis. (**A**) The representative images of the uterus in mice. (**B**) Differences in uterine indices in mice. (**C**) Bacterial load in uterine tissue. (**D**) H&E staining results of the mouse uterus. (**E**) Quantitative histopathological injury scores of uterine tissues in different groups. Values are expressed as means ± SD; Means without a common letter differ (*p* < 0.05).

Figure 3 illustrates the histopathological protective effects of the SOL aqueous extract on uterine tissues in a murine model of endometritis. Figure 3A displays representative images of uterine morphology in each experimental group three days after treatment. In the control group, the uteri exhibited uniform size, normal texture, and no evident edema. In contrast, the uteri in the model group appeared shorter, firmer, and exhibited severe edema. The uterine morphology of each treatment group administered with SOL was comparable to that of the control group, characterized by uniform size, soft texture, and absence of significant edema. The uteri in the DEXG and YSHG also showed uniform size, with mild edema and slightly increased firmness. Compared with the model group, the uterine indices in all treatment groups (except YSHG) were significantly reduced (*p* < 0.05), with no significant differences observed among these groups (*p* > 0.05). Furthermore, the uterine index in the YSHG showed a decreasing trend compared to the model group (Figure 3B).

Histopathological analysis of H&E-stained uterine sections revealed a normal architecture without inflammatory infiltration in the control group. Conversely, the model group exhibited marked pathological damage, including loose connective tissue, interstitial edema, focal lymphocyte infiltration, glandular enlargement, and eosinophilic exudates within the lumina (Figure 3D). To objectively evaluate tissue damage, a semi-quantitative histopathological scoring system was applied (Figure 3E). Quantitative analysis confirmed that the injury score of the model group was significantly elevated compared to the control group. Treatment with SOL dose-dependently attenuated these inflammatory lesions and significantly reduced the pathological scores. Specifically, minimal inflammatory cell infiltration was observed in the HSOG and YSHG, which closely resembled the control group. Slight glandular dilation and minor eosinophilic exudation remained in the LSOG and DEXG, respectively, which is consistent with their intermediate injury scores.

The bacterial load in murine uterine tissues is presented in Figure 3C. Compared with the model group, the bacterial load in the HSOG, MSOG, LSOG, DEXG, and YSHG was significantly decreased (*p* < 0.05). Integrated analysis of uterine morphology, quantitative histopathological scores, and bacterial load data collectively confirms the successful establishment of the mouse model of endometritis. Moreover, the SOL extract, dexamethasone, and Yimu Shenghua formulation all exhibited inhibitory effects on both endometritis-induced tissue damage and bacterial accumulation in the uterus.

### 3.4. SOL Alleviates Endometritis by Inhibiting the TLR4/MyD88 Signaling Pathway

**Figure 4:**
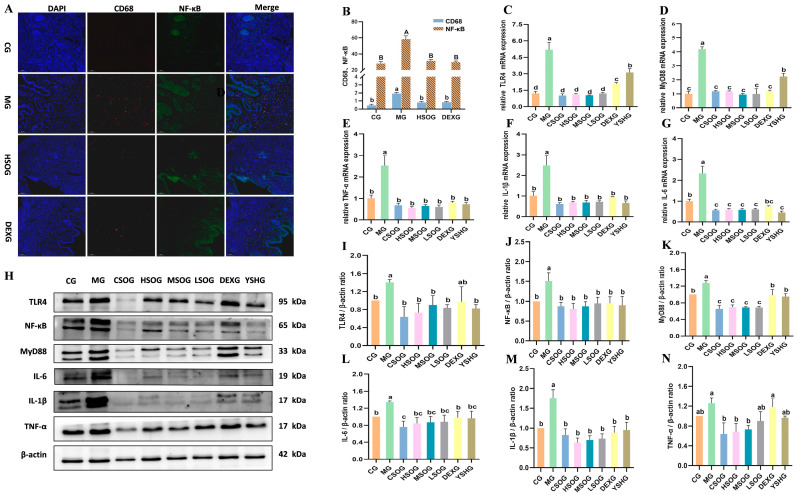
Therapeutic effects of SOL on endometritis in mice. (**A**) Immunofluorescence results and (**B**) mean fluorescence intensity of CD68 and NF-κB in mouse uterine tissues. mRNA transcription levels of TLR4 (**C**), MyD88 (**D**), TNF-α (**E**), IL-1β (**F**) and IL-6 (**G**) in mouse uterine tissues. Effects of SOL on the protein expression levels of TLR4 (**H**,**I**), NF-κB (**H**,**J**), MyD88 (**H**,**K**), IL-6 (**H**,**L**), IL-1β (**H**,**M**), and TNF-α (**H**,**N**) in mice with endometritis. Values are expressed as means ± SD; Means without a common letter differ (*p* < 0.05), (the original western blot pictures can be found in File S1).

The uterine tissues of mice were subjected to immunofluorescent double staining. CD68 was selected as a pan-macrophage marker to indicate macrophage infiltration, while NF-κB was used to assess changes in the inflammatory response. As shown in Figure 4A, the intensities of red fluorescence (CD68) and green fluorescence (NF-κB) in all treatment groups were reduced compared to the model group. Quantitative analysis confirmed statistically significant differences (*p* < 0.05) (Figure 4B). Collectively, these findings suggest that the SOL may suppress macrophage infiltration and attenuate the inflammatory response.

To investigate whether the SOL exerts a therapeutic effect on endometritis through modulation of the TLR4/MyD88 signaling, RT-PCR was employed for validation. The mRNA expression levels of key genes in the TLR4/MyD88 signaling pathway, specifically TLR4, MyD88, TNF-α, IL-1β, and IL-6, were measured in the uterine tissues of mice across different experimental groups. Compared with the model group, the mRNA expression levels of all five genes were significantly downregulated (*p* < 0.05). Furthermore, no statistically significant differences were observed in the mRNA expression levels of TLR4, MyD88, TNF-α, IL-1β, or IL-6 between the control group and the HSOG, MSOG, or LSOG (*p* > 0.05) (Figure 4C–G). These findings suggest that SOL has a notable regulatory effect on the TLR4/MyD88 signaling pathway.

Western blot analysis was further performed to evaluate the expression levels of TLR4, NF-κB (p65), MyD88, TNF-α, IL-1β, and IL-6 proteins in the uterine tissues of mice across all experimental groups. Following combined infection with *S. aureus* and *E. coli*, the expression levels of TLR4, NF-κB (p65), MyD88, IL-1β, and IL-6 in the model group were significantly higher than those in the control group (*p* < 0.05), while TNF-α showed an upward trend in expression. Compared with the control group, no significant differences were observed in the expression of these proteins in the HSOG, MSOG, and LSOG (*p* > 0.05), although MyD88 expression was significantly reduced (*p* < 0.05). Additionally, the expression levels of these proteins in the HSOG tended to be lower than those in the DEXG and YSHG. The crude drug concentrations of the HSOG and YSHG were 500 mg/mL and 1.14 g/mL, respectively, indicating that SOL still exhibited superior therapeutic efficacy at a lower crude drug concentration compared to the Yimu Shenghua formulation (Figure 4H–N).

### 3.5. SOL Extract Alleviates Endometritis via Suppression of Macrophage M1 Polarization

**Figure 5:**
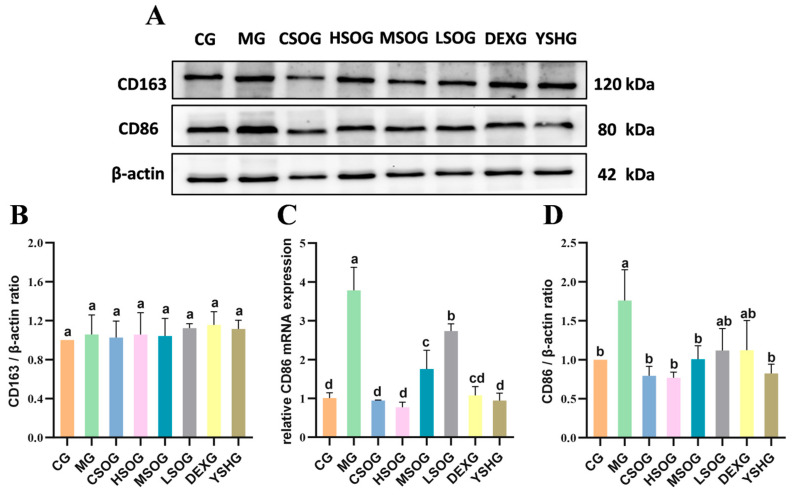
The effect of SOL on CD163 protein expression levels (**A**,**B**), CD86 mRNA expression levels (**C**), and CD86 protein expression levels (**A**,**D**) in endometritis-induced mice. Values are expressed as means ± SD; Means without a common letter differ (*p* < 0.05), (the original western blot pictures can be found in File S1).

To investigate the regulatory effect of SOL on macrophage M1 polarization, this study selected CD86 and CD163 as markers for M1 and M2 macrophages, respectively. The mRNA expression levels and protein content of CD86 in the uterine tissues of mice from each experimental group were measured, along with the protein expression levels of CD163. The results are shown in Figure 5A–D. Compared with the model group, both the mRNA transcription levels and protein expression levels of CD86 were significantly reduced in all treatment groups (*p* < 0.05). Moreover, the CD86 expression levels in the HSOG, MSOG, and LSOG exhibited a decreasing trend with increasing dosage (*p* < 0.05). In addition, no significant differences were observed in CD163 protein expression among the groups. These findings indicate that SOL inhibits macrophage polarization toward the M1 phenotype in a dose-dependent manner. Furthermore, the development of endometritis in mice is not significantly associated with M2 macrophage polarization, and SOL does not exert a notable regulatory effect on M2 macrophage polarization.

### 3.6. In Vitro Validation of the Anti-Inflammatory Effects of SOL in RAW264.7 Macrophages

**Figure 6:**
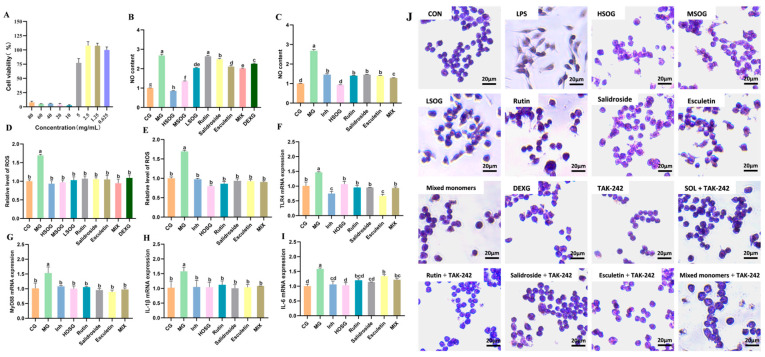
SOL suppresses LPS-induced inflammation in RAW264.7 cells in vitro. (**A**) Cell viability assessed using the CCK-8 assay. Effects of SOL, monomers, and MIX on NO (**B**,**C**) and ROS (**D**,**E**) production in the absence (**B**,**D**) or presence (**C**,**E**) of the TLR4 inhibitor TAK-242. mRNA expression levels of TLR4 (**F**), MyD88 (**G**), IL-1β (**H**), and IL-6 (**I**) measured under TAK-242 treatment to assess involvement of the TLR4/MyD88 pathway. (**J**) Representative microscopic images showing the morphological changes of RAW264.7 cells under different treatments (original magnification × 400). Values are expressed as means ± SD; Means without a common letter differ (*p* < 0.05).

To further verify the anti-inflammatory mechanisms of SOL observed in vivo, a series of cellular assays were performed using LPS-stimulated RAW264.7 macrophages. The CCK-8 assay showed that SOL concentrations up to 2.5 mg/mL maintained cell viability above 90%, while higher concentrations reduced viability in a dose-dependent manner (*p* < 0.05). Therefore, 2.5 mg/mL was selected as the maximal non-cytotoxic concentration for subsequent experiments (Figure 6A). Under LPS stimulation, RAW264.7 macrophages exhibited markedly elevated production of NO and ROS compared with the control group (*p* < 0.01). Pretreatment with SOL and its principal monomeric constituents—rutin, salidroside, and esculetin—significantly reduced both NO and ROS levels (*p* < 0.05), with the SOL and mixed-monomer groups showing the most pronounced inhibition. Co-treatment with the selective TLR4 inhibitor TAK-242 further attenuated NO and ROS generation; notably, high-dose SOL continued to suppress NO and ROS even in the presence of TAK-242 (*p* < 0.05). This finding indicates that, beyond blocking TLR4/MyD88 signaling, SOL may also modulate additional redox-regulatory pathways involved in macrophage activation (Figure 6B–E). To elucidate whether such inhibition affected canonical inflammatory signaling, the mRNA levels of TLR4, MyD88, IL-1β, and IL-6 were quantified in TAK-242–treated cells. In TAK-242–treated macrophages, SOL, monomeric compounds, and their mixture further reduced the mRNA expression of TLR4, MyD88, IL-6, and IL-1β compared with TAK-242 alone (*p* < 0.05), confirming a synergistic suppression of inflammatory transcriptional activity while preserving TLR4 pathway fidelity (Figure 6F–I).

Morphological observations supported these findings (Figure 6J). In the control group, macrophages exhibited small, rounded cell bodies with clear boundaries. LPS stimulation induced pronounced morphological activation—cells became flattened and extended multiple pseudopodia, consistent with M1-type polarization. Treatment with SOL or its monomeric constituents markedly alleviated these LPS-induced changes, restoring a more rounded, quiescent morphology. The mixed-monomer and SOL groups showed the greatest recovery, resembling control cells. Moreover, TAK-242 co-treatment enhanced this morphological normalization, suggesting that SOL mitigates macrophage activation at least partly through TLR4/MyD88-dependent regulation (Figure 6). Collectively, these results indicate that SOL exerts potent anti-inflammatory and antioxidant effects in activated macrophages by suppressing LPS-induced oxidative stress and cytokine production, consistent with its in vivo modulation of the TLR4/MyD88 pathway.

## 4. Discussion

Endometritis remains a major reproductive disorder compromising fertility and productivity in livestock, with increasing concerns over antimicrobial resistance driving the search for safer, plant-derived alternatives [15,16]. Plant-derived compounds and standardized botanical extracts are attractive candidates in this regard because they frequently act on multiple targets and pathways and often show favorable safety profiles in preclinical studies [4,17]. Among candidate botanicals, *Syringa oblata* Lindl. (Oleaceae) has a long tradition of ethnomedicinal use, and recent phytochemical and pharmacological reviews indicate that leaves and other aerial parts are rich in flavonoids, phenylethanoid glycosides and related phenolics—classes of compounds with documented anti-inflammatory and antimicrobial activities [5,18]. However, despite this phytochemical promise, SOL has not been systematically evaluated in disease-relevant models of reproductive tract infection. Our study addresses this gap, demonstrating that the aqueous extract of SOL effectively alleviates murine bacterial endometritis through coordinated antimicrobial actions and immunomodulation.

UPLC-MS/MS and network pharmacology identified rutin, salidroside, and esculetin as principal active constituents of *Syringa oblata* leaf extract. These compounds are known to exert anti-inflammatory and antioxidant effects by suppressing NF-κB, MAPK, and Nrf2-related signaling pathways [19,20,21]. This multi-target potential is further corroborated by our GO and KEGG enrichment analyses, which revealed a broad modulation of classical inflammatory and immune cascades, including the cytokine-mediated and Toll-like receptor signaling pathways. In our in vitro models, a mixture of the three commercial monomers significantly suppressed LPS-induced NO and ROS production. However, it is crucial to acknowledge that this simplified monomer mixture cannot fully represent the complex pharmacological profile of the crude SOL extract. Chinese herbal medicines characteristically exert their therapeutic effects through an intricate multi-component matrix. Beyond these three major compounds, our UPLC-MS/MS profiling identified 10 core disease-associated compounds, including various lignans and other phenolics. The superior morphological recovery and anti-inflammatory efficacy observed in the whole SOL extract group compared to the pure monomer mixture strongly suggest synergistic or additive interactions among these diverse phytochemicals. This synergy is a hallmark of botanical medicines, enabling them to target multiple distinct nodes within the inflammatory cascade simultaneously.

The TLR4/MyD88/NF-κB signaling pathway serves as a central driver of pathogen-induced uterine inflammation, linking bacterial recognition to downstream cytokine production and tissue injury. Excessive activation of this axis amplifies the transcription of pro-inflammatory cytokines such as IL-1β, IL-6, and TNF-α, disrupting endometrial homeostasis and compromising reproductive function [11,12]. In the present study, both in vivo and in vitro experiments demonstrated that SOL effectively blocks this TLR4-dependent innate immune cascade, as evidenced by significant histopathological restoration and the robust downregulation of downstream inflammatory mediators. Beyond canonical inflammatory cascades, SOL suppressed LPS-induced oxidative stress indicators. Notably, high-dose SOL continued to reduce NO and ROS levels even under pharmacological TLR4 inhibition with TAK-242. This observation aligns with increasing evidence that plant flavonoids exert parallel modulation of redox-sensitive pathways such as MAPK and Nrf2/HO-1, contributing to the attenuation of oxidative stress and inflammatory amplification [22,23]. This multifaceted action reflects a broader pharmacological paradigm where diverse plant-derived flavonoids and extracts combat endometritis through highly complementary mechanisms. For instance, astilbin, a natural flavonoid isolated from *Smilax china* L., mitigates uterine inflammation by interrupting the positive feedback loop between TLR4 and IL-6R signaling in a PPAR-γ-dependent manner [24]. Similarly, the total flavonoids derived from *Clinopodium chinense* confer significant protection against LPS-induced murine endometritis by specifically targeting NLRP3 inflammasome-mediated pyroptosis [25]. Furthermore, recent findings reveal that hyperoside, a widely distributed dietary flavonoid, exerts its anti-endometritic effects through a novel gut-uterus axis, where its microbiota-derived metabolites suppress uterine TLR4 activation [26]. Analogous to these well-characterized botanical agents, SOL likely operates through an integrated mechanism that simultaneously dampens TLR4/MyD88-driven cytokine cascades and reinforces endogenous antioxidant defenses, collectively preventing infection-induced uterine tissue damage.

Macrophage polarization represents a terminal effector response downstream of TLR4/NF-κB activation and plays a pivotal role in shaping the uterine inflammatory microenvironment [27,28]. Classically activated M1 macrophages, characterized by elevated CD86 expression and pro-inflammatory cytokine release, exacerbate tissue injury, whereas alternatively activated M2 macrophages expressing CD163 facilitate resolution and repair [29]. Dysregulated polarization toward the M1 phenotype has been closely linked to chronic endometritis and uterine fibrosis [14,30]. In the present study, SOL treatment markedly downregulated CD86 expression in murine uterine tissue while maintaining stable CD163 levels, suggesting selective inhibition of M1 polarization without excessive M2 skewing. These findings align with accumulating evidence that plant-derived flavonoids such as quercetin and luteolin modulate macrophage phenotypes and inflammatory tone via AKT1–FoxO1, Nrf2, and MAPK signaling cascades [31,32]. Such immunoregulatory effects reflect a broader pharmacological paradigm in ethnopharmacology: multi-component botanical extracts can achieve both antimicrobial and host-directed actions, restoring immune equilibrium and protecting tissue integrity through the simultaneous regulation of inflammatory and oxidative pathways.

While the present study demonstrates significant advantages by providing comprehensive mechanistic and histopathological evidence for the multi-target therapeutic potential of SOL, several limitations must be acknowledged. First, the use of a murine model and a limited set of in vitro assays, though mechanistically informative, may not fully capture the complex pharmacokinetics and immune dynamics of target livestock species. Second, our in vitro substitution of the crude extract with a three-monomer mixture fundamentally oversimplifies the complex synergy of the whole botanical matrix. Future studies should therefore incorporate pharmacokinetic profiling, metabolite identification, and validation in clinically relevant animal models such as sows or dairy cows to strengthen translational applicability. Additionally, exploration of other potential signaling routes, such as Nrf2/HO-1, STAT3, and autophagy-related pathways, will be necessary to fully delineate its multitarget effects.

## 5. Conclusions

In conclusion, this study demonstrates that the aqueous extract of *Syringa oblata* Lindl. effectively alleviates bacterial endometritis by inhibiting the TLR4/MyD88 signaling pathway and modulating macrophage polarization. From an ethnopharmacological perspective, this study validates the traditional use of Syringa species in inflammatory conditions and extends their relevance to reproductive tract disorders. Considering the growing challenge of antibiotic resistance and the need for safe veterinary alternatives, SOL emerges as a promising candidate for the prevention and management of endometritis.

## Figures and Tables

**Table 1:** HPLC analysis conditions.

Ingredients	Injection Volume	Absorption Wavelength	Flow Rate	Mobile Phase	Elution Procedure
Rutin	10 µL	355 nm	1.0 mL/min	A: 0.1% formic acid aqueous solution B: acetonitrile	0–30 min: 95–47% A 30–35 min: 47–95% A 35–36 min: 95% A
Salidroside	10 µL	275 nm		A: water B: methanol	A–B (80:20 *v*/*v*)
Esculetin	20 µL	352 nm		A: 0.1% phosphoric acid aqueous solution B: acetonitrile	A–B (88:12 *v*/*v*)

**Table 2:** Experimental grouping and daily intrauterine administration schedule.

Group	Description	Day 1–3 (Infection Phase)	Days 4–6 (Treatment Phase)	Day 7
CG	Control Group	Normal saline	Normal saline	Euthanasia
MG	Model Group	Bacterial suspension	Normal saline	
CSOG	*S. oblata* Control	Normal saline	SOL (500 mg/mL)	
HSOG	High-dose SOL	Bacterial suspension	SOL (500 mg/mL)	
MSOG	Medium-dose SOL	Bacterial suspension	SOL (375 mg/mL)	
LSOG	Low-dose SOL	Bacterial suspension	SOL (250 mg/mL)	
DEXG	Positive Chemical Control	Bacterial suspension	DEX (5 mg/kg)	
YSHG	Traditional Chinese Medicine Control	Bacterial suspension	YSH (1.14 g/mL)	

**Table 3:** Primer sequences used for RT-PCR.

Gene	Forward Primer Sequence (5′-3′)	Reverse Primer Sequence (5′-3′)
*β-actin*	GTGCTATGTTGCTCTAGACTTCG	ATGCCACAGGATTCCATACC
*TLR4*	TTGCTGCCAACATCATCCAGGAAG	ACCAACGGCTCTGAATAAAGTGTCTAG
*MyD88*	GGAGCCAGATTCTCTGATGC	GAGCTGTCCCAAAGGAAACA
*CD86*	TCTGCCGTGCCCATTTACAAAGG	TGCCCAAATAGTGCTCGTACAGAAC
*IL-1β*	TCCAGGATGAGGACATGAGCAC	GAACGTCACACACCAGCAGGTTA
*IL-6*	TTCTTGGGACTGATGCTGGTGAC	AGTGGTATCCTCTGTGAAGTCTCCTC
*TNF-α*	TATGGCCCAGACCCTCACA	GGAGTAGACAAGGTACAACCCATC

**Table 4:** Compounds associated with the active ingredients.

Name	Formula	Molecular Weight	Value	Ionization Model	Class
Rutin	C_27_H_30_O_16_	610.1534	7	[M + H]+	Flavonols
Salidroside	C_14_H_20_O_7_	300.1209	5	[M − H]−	Phenolic acids
Esculetin	C_9_H_6_O_4_	178.0266	3	[M − H]−	Coumarins
Secoisolariciresinol	C_20_H_26_O_6_	362.1729	3	[M + H]+	Lignans
Dihydrocubebin	C_20_H_22_O_6_	358.1416	3	[M + H]+	Lignans
Dehydrodiconiferyl alcohol	C_20_H_22_O_6_	358.1416	3	[M + H]+	Lignans
2,4-dihydroxy-6-methoxyacetophenone	C_9_H_10_O_4_	182.0579	3	[M + H]+	Ketone compounds
Embelin	C_17_H_26_O_4_	294.1831	2	[M − H]−	Quinones
Danshensu (Salvianic Acid A)	C_9_H_10_O_5_	198.0528	2	[M − H]−	Phenolic acids
Vanillin	C_8_H_8_O_3_	152.0473	2	[M − H]−	Aldehyde compounds

## Data Availability

The original contributions presented in this study are included in the article/supplementary material. Further inquiries can be directed to the corresponding author.

## Supplementary Materials

The following supporting information can be downloaded at: https://www.mdpi.com/article/10.3390/vetsci13060526/s1, Table S1: UPLC-MS/MS analysis of the SOL aqueous extract. File S1: Original images of Western-blotting.

## References

[1] Ticconi C., Inversetti A., Marraffa S., Campagnolo L., Arthur J., Zambella E., Di Simone N. (2024). Chronic endometritis and recurrent reproductive failure: A systematic review and meta-analysis. Front. Immunol..

[2] Li J., Wei J., Chen S., Wang X., Chen J., Zeng D., Fan L. (2024). Prevalence and risk factors for chronic endometritis in patients with adenomyosis and infertility: A retrospective cohort study. BMC Women’s Health.

[3] Silva J.C.C., Caldeira M.O., Moraes J.G.N., Sellmer Ramos I., Gull T., Ericsson A.C., Poock S.E., Spencer T.E., Lucy M.C. (2024). Metritis and the uterine disease microbiome are associated with long-term changes in the endometrium of dairy cows. Biol. Reprod..

[4] Swaroop A.K., Lalitha C., Shanmugam M., Subramanian G., Natarajan J., Selvaraj J. (2022). Plant Derived Immunomodulators; A Critical Review. Adv. Pharm. Bull..

[5] Zhu W., Wang Z., Sun Y., Yang B., Wang Q., Kuang H. (2021). Traditional uses, phytochemistry and pharmacology of genus *Syringa*: A comprehensive review. J. Ethnopharmacol..

[6] Wang X.Z., Song X.J., Liu C., Xing C., Wu T., Zhang Y., Su J., Hao J.Y., Chen X.Y., Zhang Z.Y. (2022). Active components and molecular mechanism of *Syringa oblata* Lindl. in the treatment of endometritis based on pharmacology network prediction. Front. Vet. Sci..

[7] Ding Y., Wen G., Wei X., Zhou H., Li C., Luo Z., Ou D., Yang J., Song X. (2024). Antibacterial activity and mechanism of luteolin isolated from *Lophatherum gracile* Brongn. against multidrug-resistant *Escherichia coli*. Front. Pharmacol..

[8] Chen S., Bai Y., Xia J., Zhang Y., Zhan Q. (2023). Rutin alleviates ventilator-induced lung injury by inhibiting NLRP3 inflammasome activation. iScience.

[9] Ye G., Huang J., Li G., Zhang J., Sun Y., Zeng D., Bao W., Zhong J., Huang Q. (2021). Clinical efficacy of intravaginal recombinant lysostaphin administration on endometritis in sows. Vet. Med. Sci..

[10] Yin P., Zhang Z., Li J., Shi Y., Jin N., Zou W., Gao Q., Wang W., Liu F. (2019). Ferulic acid inhibits bovine endometrial epithelial cells against LPS-induced inflammation via suppressing NK-κB and MAPK pathway. Res. Vet. Sci..

[11] Guo B., Chen J.H., Zhang J.H., Fang Y., Liu X.J., Zhang J., Zhu H.Q., Zhan L. (2023). Pattern-recognition receptors in endometriosis: A narrative review. Front. Immunol..

[12] Bao H., Cong J., Qu Q., He S., Zhao D., Zhao H., Yin S., Ma D. (2024). Rosiglitazone alleviates LPS-induced endometritis via suppression of TLR4-mediated NF-κB activation. PLoS ONE.

[13] Wang S., Xu S., Zhou J., Zhang L., Mao X., Yao X., Liu C. (2021). Luteolin transforms the polarity of bone marrow-derived macrophages to regulate the cytokine storm. J. Inflamm..

[14] You S., Zhu Y., Li H., He F., Liu S., Yang X., Wang L., Zeng H., Dai J., Hu L. (2023). Recombinant humanized collagen remodels endometrial immune microenvironment of chronic endometritis through macrophage immunomodulation. Regen. Biomater..

[15] Várhidi Z., Csikó G., Bajcsy Á.C., Jurkovich V. (2024). Uterine Disease in Dairy Cows: A Comprehensive Review Highlighting New Research Areas. Vet. Sci..

[16] Pandey S., Doo H., Keum G.B., Kim E.S., Kwak J., Ryu S., Choi Y., Kang J., Kim S., Lee N.R. (2024). Antibiotic resistance in livestock, environment and humans: One Health perspective. J. Anim. Sci. Technol..

[17] Zebeaman M., Tadesse M.G., Bachheti R.K., Bachheti A., Gebeyhu R., Chaubey K.K. (2023). Plants and Plant-Derived Molecules as Natural Immunomodulators. BioMed Res. Int..

[18] Tai B., Bai L., Ji R., Yu M., Nala, Huang L., Zheng H. (2022). Phytochemical and pharmacological progress on *Syringa oblata*, a traditional Mongolian medicine. Chin. Herb. Med..

[19] Jang H., Han S.C., Lee J., Shin H.Y., Hwang J.H., Ha J.H. (2025). Anti-inflammatory effects of rutin in lipopolysaccharide-stimulated canine macrophage cells. Nutr. Res. Pract..

[20] Wang X.L., Sun R.X., Li D.X., Chen Z.G., Li X.F., Sun S.Y., Lin F., Zhao G.A. (2023). Salidroside Regulates Mitochondrial Homeostasis After Polarization of RAW264.7 Macrophages. J. Cardiovasc. Pharmacol..

[21] Wang S.K., Chen T.X., Wang W., Xu L.L., Zhang Y.Q., Jin Z., Liu Y.B., Tang Y.Z. (2022). Aesculetin exhibited anti-inflammatory activities through inhibiting NF-κB and MAPKs pathway in vitro and in vivo. J. Ethnopharmacol..

[22] Bilski R., Nuszkiewicz J. (2025). Antioxidant Therapies as Emerging Adjuncts in Rheumatoid Arthritis: Targeting Oxidative Stress to Enhance Treatment Outcomes. Int. J. Mol. Sci..

[23] John O.D., Mushunje A.T., Surugau N., Guad R.M. (2022). The metabolic and molecular mechanisms of α-mangostin in cardiometabolic disorders (Review). Int. J. Mol. Med..

[24] Feng H., Li C., Chen J., Li Z., Ye X., Hou L., Wang C., Hou C., Liu W. (2025). Astilbin from *Smilax china* L. remarkably inhibits LPS-induced endometritis in rats via blocking positive feedback between TLR4 and IL-6R signalling pathways in a PPAR-γ-dependent manner. J. Ethnopharmacol..

[25] Li L., Qi J., Tao H., Wang L., Wang L., Wang N., Huang Q. (2023). Protective effect of the total flavonoids from Clinopodium chinense against LPS-induced mice endometritis by inhibiting NLRP3 inflammasome-mediated pyroptosis. J. Ethnopharmacol..

[26] Yang J., Yu J., Chen Y., Xu A., Yang C., Li J., Wu F., Li X., Bi J., Xiang B. (2026). Hyperoside, a dietary flavonoid, protects against endometritis via gut microbiota-dependent production of hydroxyphenyllactic acid and the gut-uterus axis. Food Funct..

[27] Liu B., Yu J., Zhang J., Ye W., Yao J. (2025). TLR4/NF-κB-mediated M1 macrophage polarization contributes to the promotive effects of ETS2 on ulcerative colitis. Eur. J. Med. Res..

[28] Firmal P., Shah V.K., Chattopadhyay S. (2020). Insight Into TLR4-Mediated Immunomodulation in Normal Pregnancy and Related Disorders. Front. Immunol..

[29] Strizova Z., Benesova I., Bartolini R., Novysedlak R., Cecrdlova E., Foley L.K., Striz I. (2023). M1/M2 macrophages and their overlaps—Myth or reality?. Clin. Sci..

[30] Li J.W., Wan R.T., Liu Q.D., Xu H.L., Chen Q. (2024). Causal association of immune cells and endometritis: A Mendelian randomization study. Sci. Rep..

[31] Xu J., Li Y., Yang X., Li H., Xiao X., You J., Li H., Zheng L., Yi C., Li Z. (2024). Quercetin inhibited LPS-induced cytokine storm by interacting with the AKT1-FoxO1 and Keap1-Nrf2 signaling pathway in macrophages. Sci. Rep..

[32] Gao S., Gao Y., Cai L., Qin R. (2024). Luteolin attenuates Staphylococcus aureus-induced endometritis through inhibiting ferroptosis and inflammation via activating the Nrf2/GPX4 signaling pathway. Microbiol. Spectr..

