# Supplementary figures and images for: Aqueous Extract of Syringa oblata Lindl. Alleviates Murine Endometritis by Modulating TLR4/MyD88 Signaling and Macrophage Polarization (part 2 of 2)
Source: Vet Sci. 2026 May 28;13(6):526. doi: 10.3390/vetsci13060526 (PMC13308303; doi:10.3390/vetsci13060526)

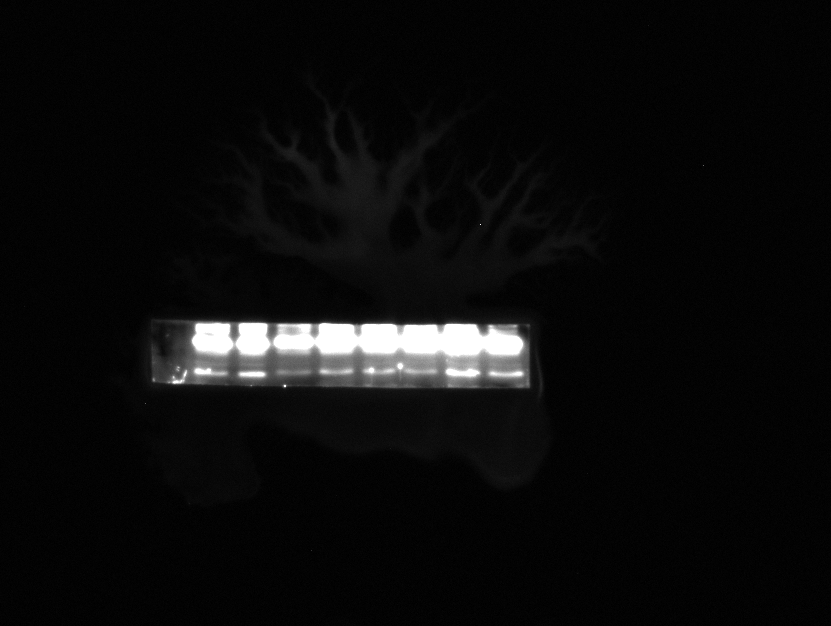

Supplement: Supplementary file 1 [file vetsci-13-00526-s001.zip › Supplementary file S1/Original Images wb/TNFα -2/contrast/contrast_6.png]

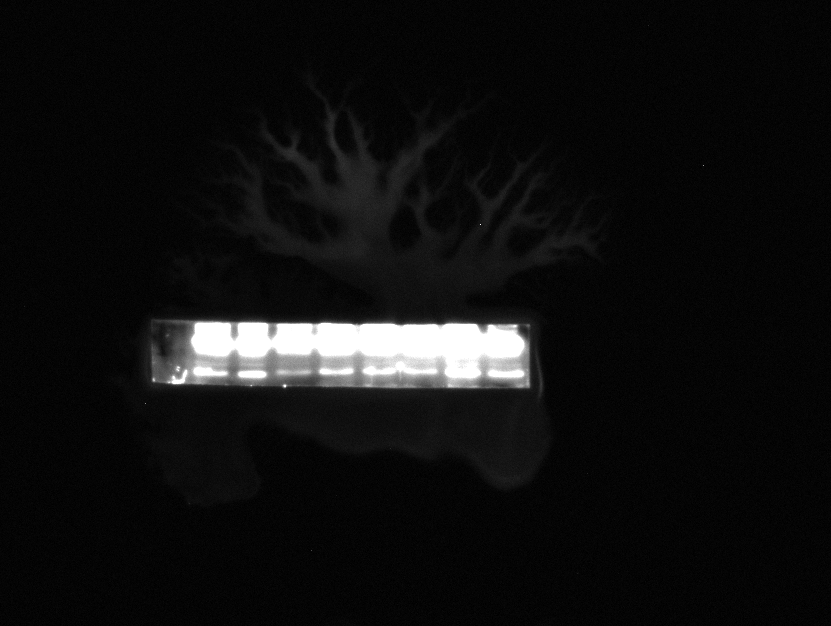

Supplement: Supplementary file 1 [file vetsci-13-00526-s001.zip › Supplementary file S1/Original Images wb/TNFα -2/contrast/contrast_7.png]

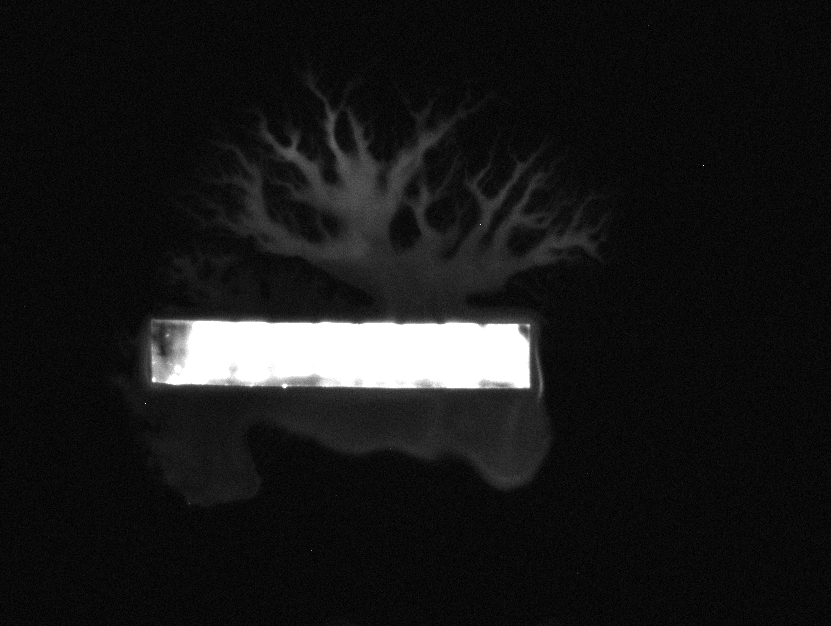

Supplement: Supplementary file 1 [file vetsci-13-00526-s001.zip › Supplementary file S1/Original Images wb/TNFα -2/contrast/contrast_8.png]

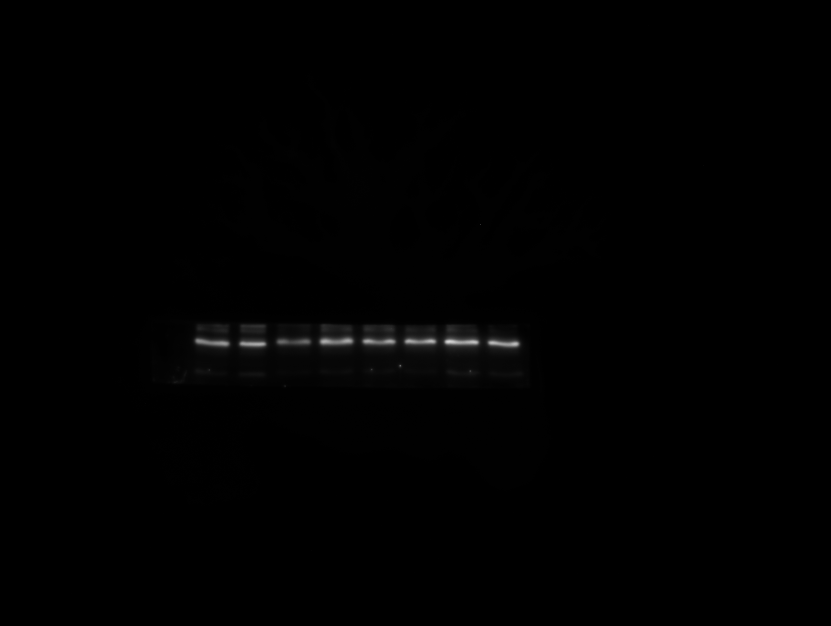

Supplement: Supplementary file 1 [file vetsci-13-00526-s001.zip › Supplementary file S1/Original Images wb/TNFα -2/Tnf-1-2023-10-27_14-59-03_1_16bit.png]

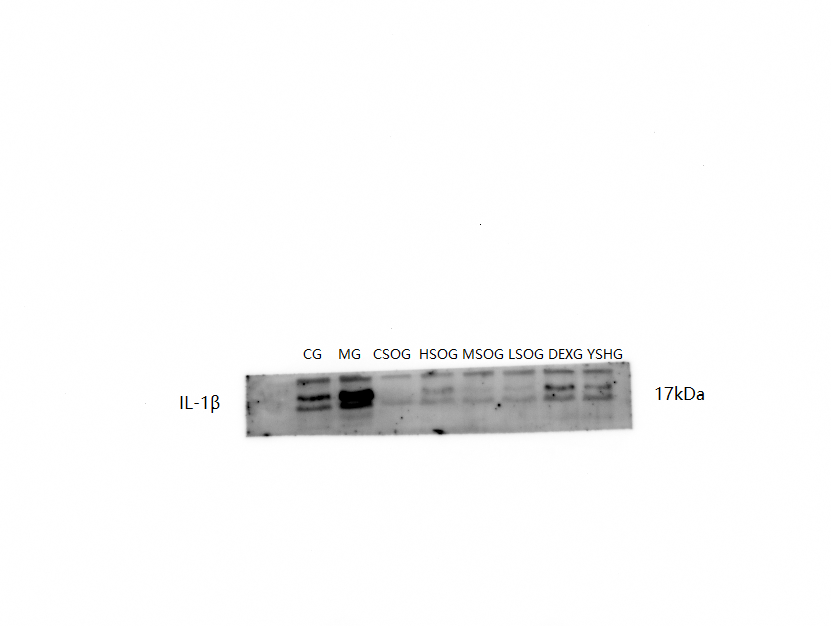

Supplement: Supplementary file 1 [file vetsci-13-00526-s001.zip › Supplementary file S1/Original Images WB marked/Fig.4 IL-1β.png]

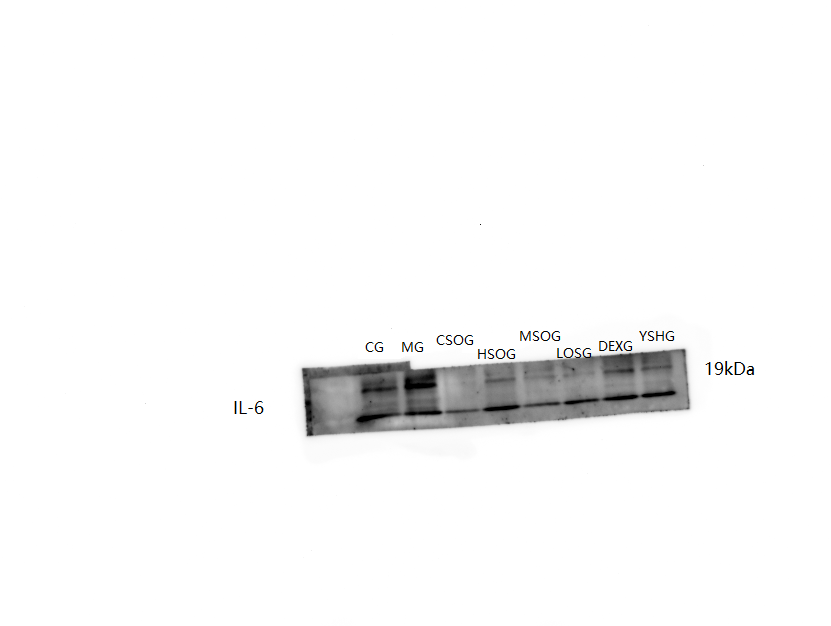

Supplement: Supplementary file 1 [file vetsci-13-00526-s001.zip › Supplementary file S1/Original Images WB marked/Fig.4 IL-6.png]

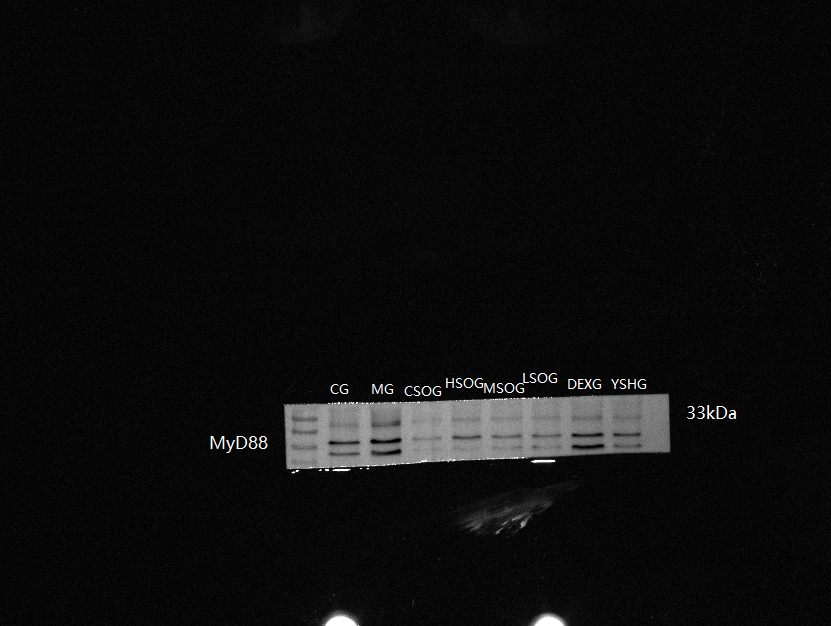

Supplement: Supplementary file 1 [file vetsci-13-00526-s001.zip › Supplementary file S1/Original Images WB marked/Fig.4 MyD88.png]

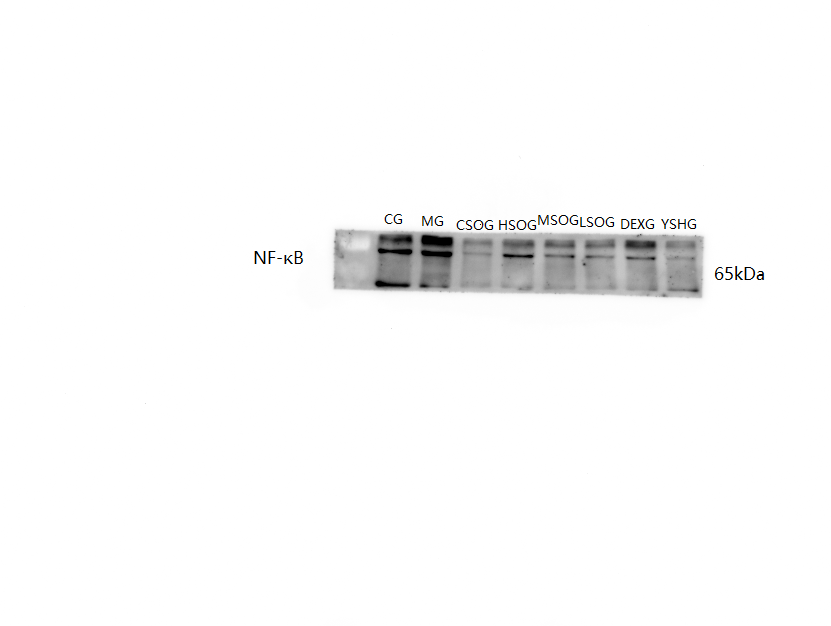

Supplement: Supplementary file 1 [file vetsci-13-00526-s001.zip › Supplementary file S1/Original Images WB marked/Fig.4 Nf-κB.png]

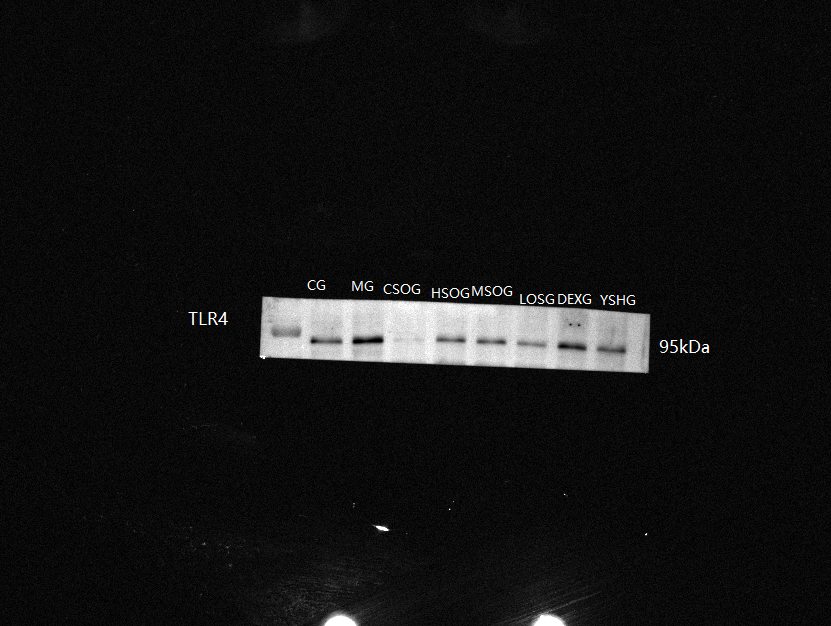

Supplement: Supplementary file 1 [file vetsci-13-00526-s001.zip › Supplementary file S1/Original Images WB marked/Fig.4 Tlr4.png]

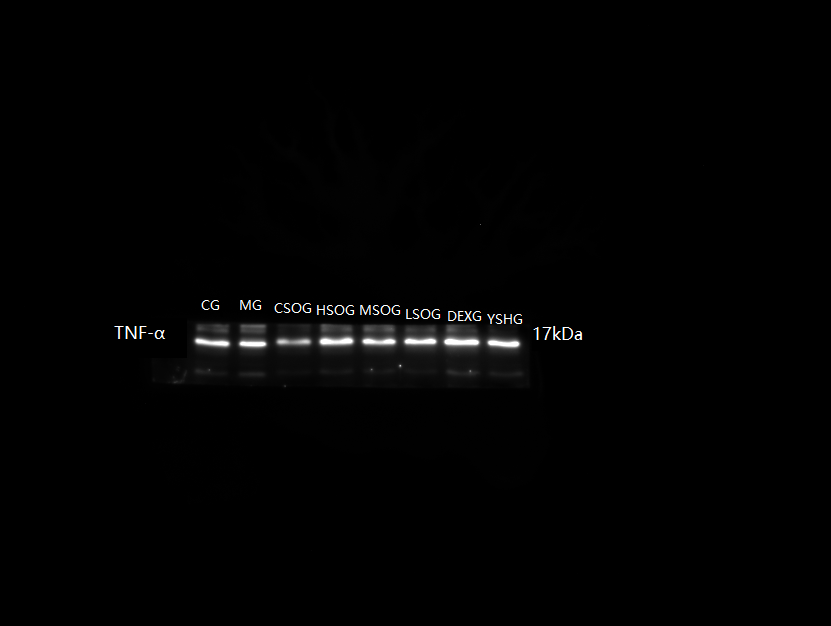

Supplement: Supplementary file 1 [file vetsci-13-00526-s001.zip › Supplementary file S1/Original Images WB marked/Fig.4 TNF-α.png]

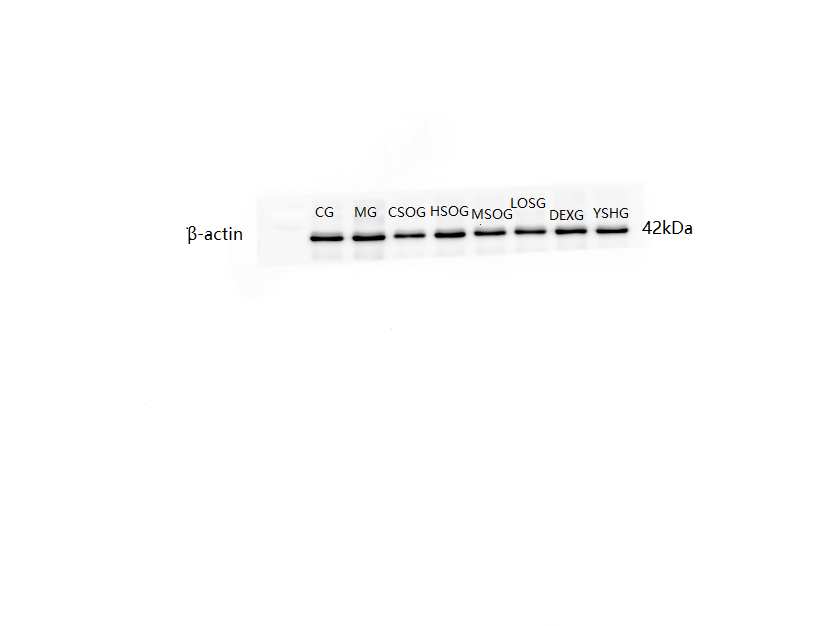

Supplement: Supplementary file 1 [file vetsci-13-00526-s001.zip › Supplementary file S1/Original Images WB marked/Fig.4,5 β-actin.png]

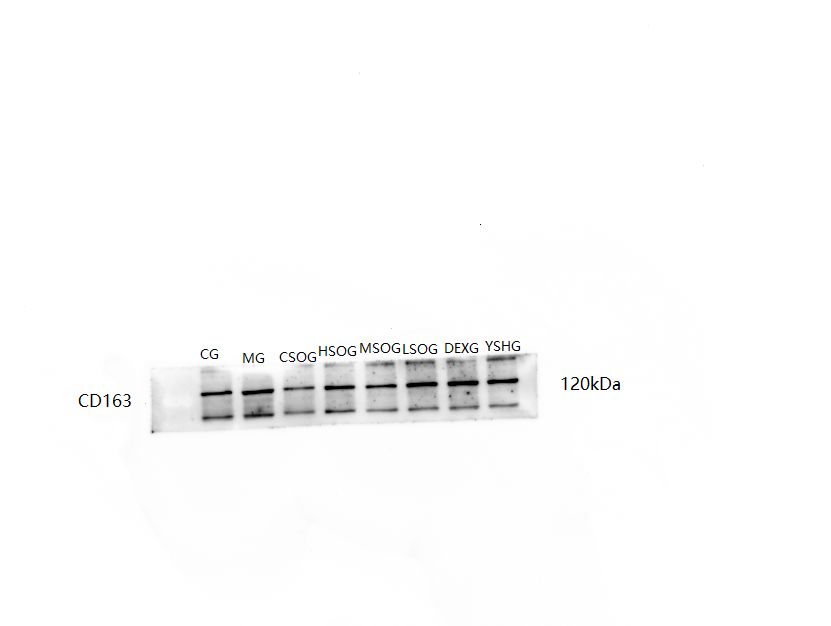

Supplement: Supplementary file 1 [file vetsci-13-00526-s001.zip › Supplementary file S1/Original Images WB marked/Fig.5 CD163.png]

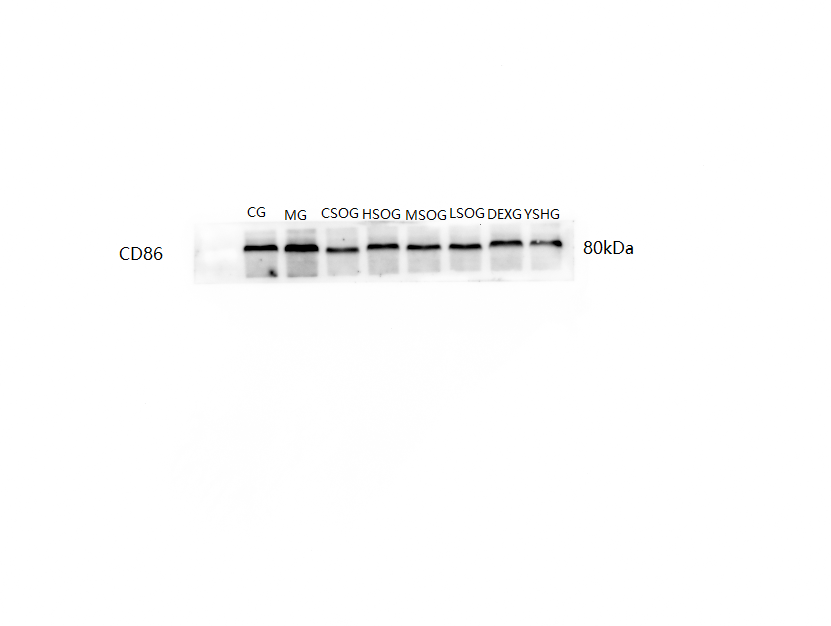

Supplement: Supplementary file 1 [file vetsci-13-00526-s001.zip › Supplementary file S1/Original Images WB marked/Fig.5 Cd86.png]
