# Supplementary figures and images for: Aqueous Extract of Syringa oblata Lindl. Alleviates Murine Endometritis by Modulating TLR4/MyD88 Signaling and Macrophage Polarization (part 1 of 2)
Source: Vet Sci. 2026 May 28;13(6):526. doi: 10.3390/vetsci13060526 (PMC13308303; doi:10.3390/vetsci13060526)

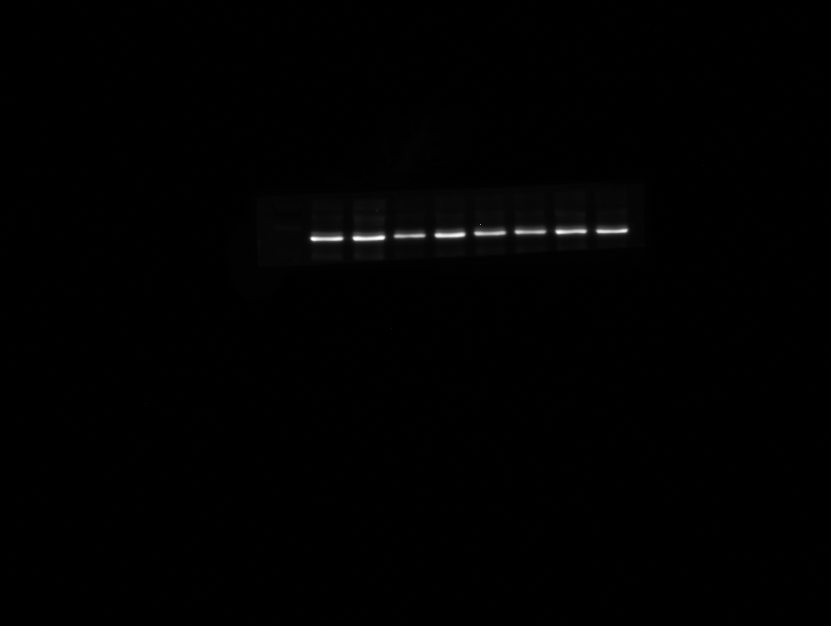

Supplement: Supplementary file 1 [file vetsci-13-00526-s001.zip › Supplementary file S1/Original Images wb/Actin/Ac-3-2023-11-30_12-36-12_1_16bit.png]

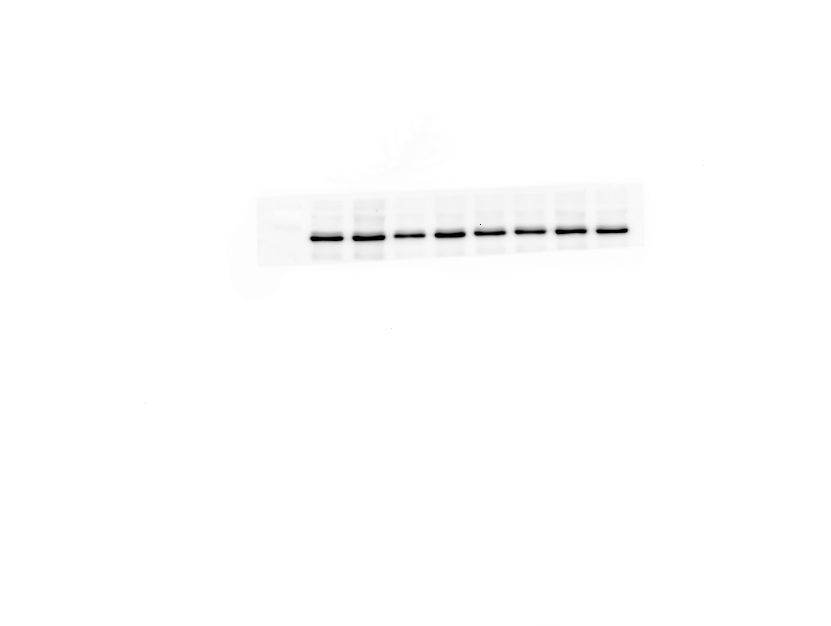

Supplement: Supplementary file 1 [file vetsci-13-00526-s001.zip › Supplementary file S1/Original Images wb/Actin/Ac-3-2023-11-30_12-36-12_8bit.png]

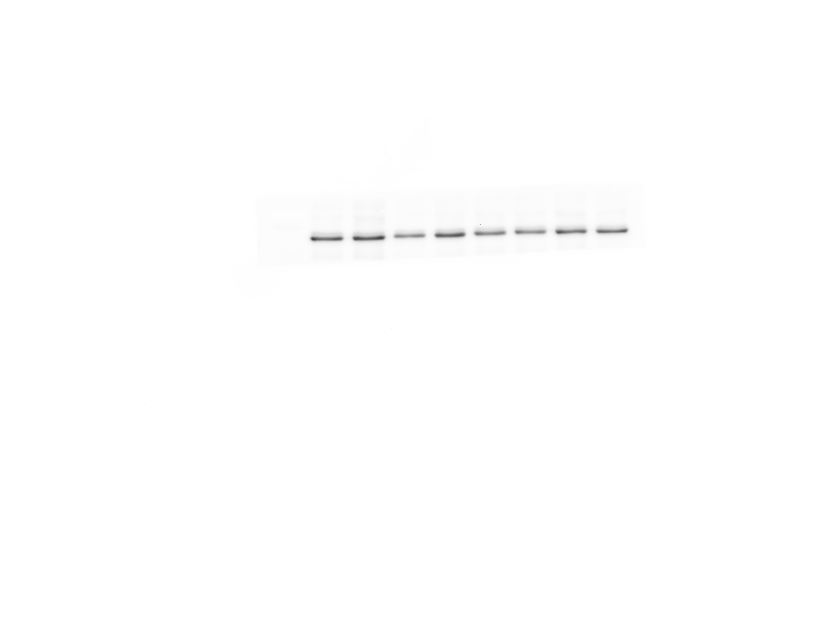

Supplement: Supplementary file 1 [file vetsci-13-00526-s001.zip › Supplementary file S1/Original Images wb/Actin/contrast/contrast_0.png]

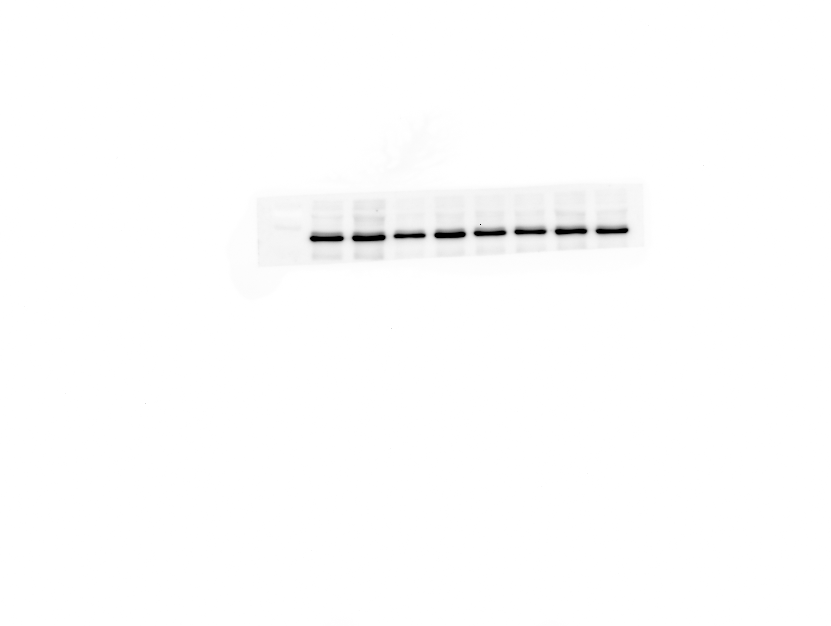

Supplement: Supplementary file 1 [file vetsci-13-00526-s001.zip › Supplementary file S1/Original Images wb/Actin/contrast/contrast_2.png]

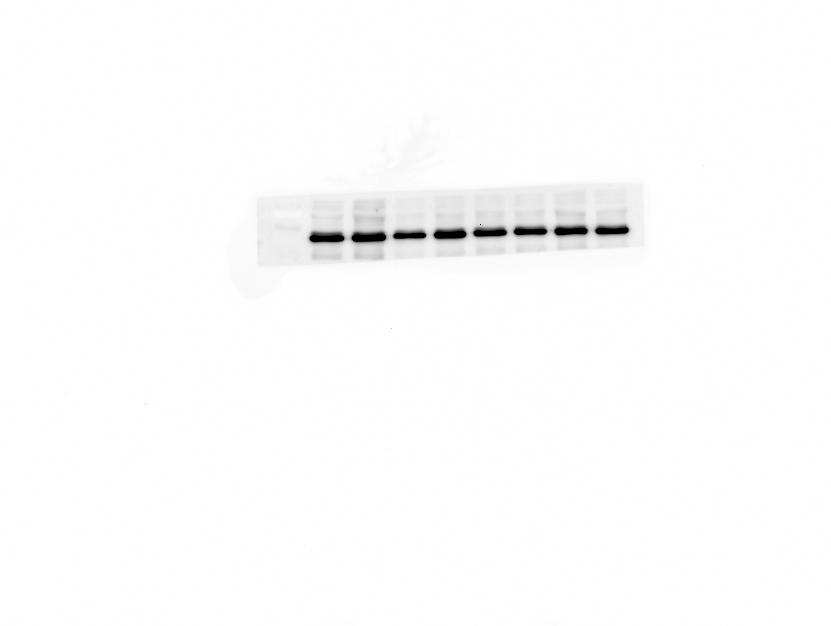

Supplement: Supplementary file 1 [file vetsci-13-00526-s001.zip › Supplementary file S1/Original Images wb/Actin/contrast/contrast_3.png]

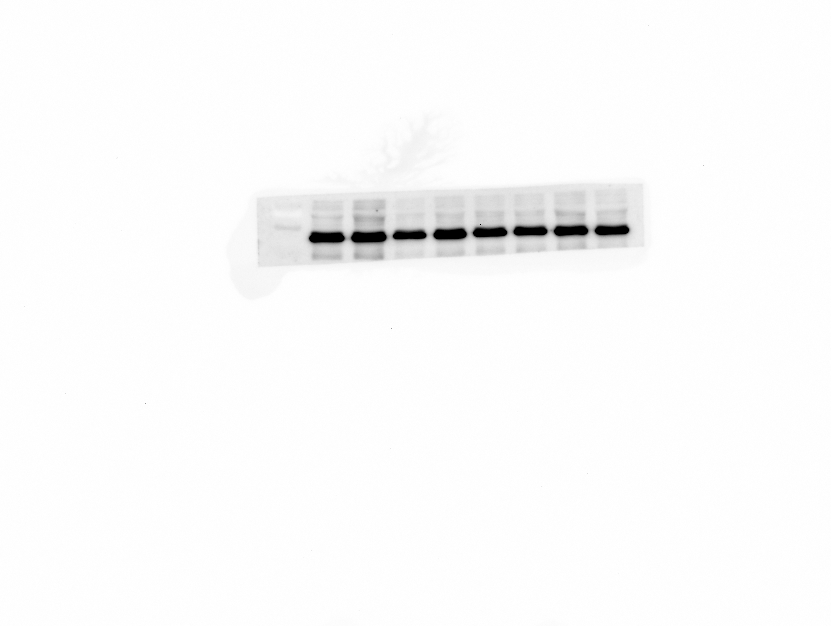

Supplement: Supplementary file 1 [file vetsci-13-00526-s001.zip › Supplementary file S1/Original Images wb/Actin/contrast/contrast_4.png]

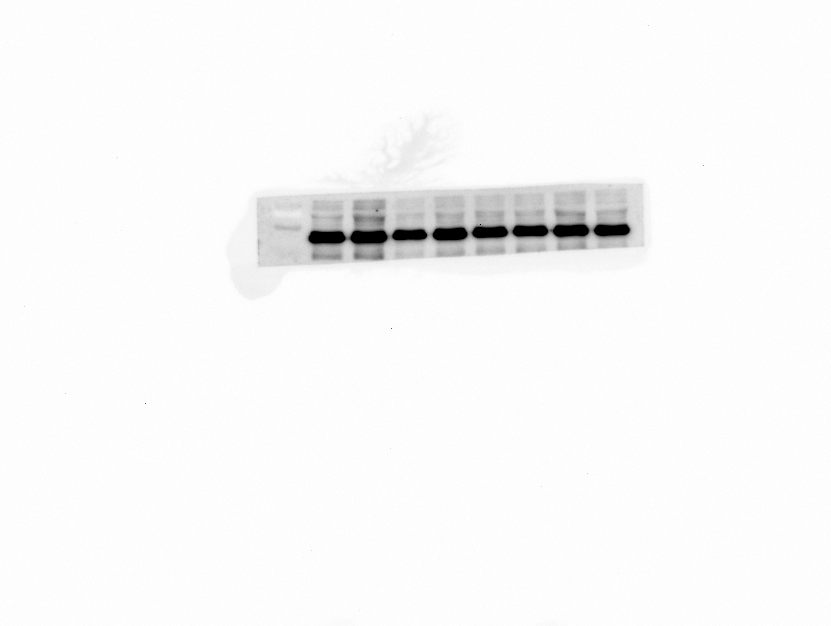

Supplement: Supplementary file 1 [file vetsci-13-00526-s001.zip › Supplementary file S1/Original Images wb/Actin/contrast/contrast_5.png]

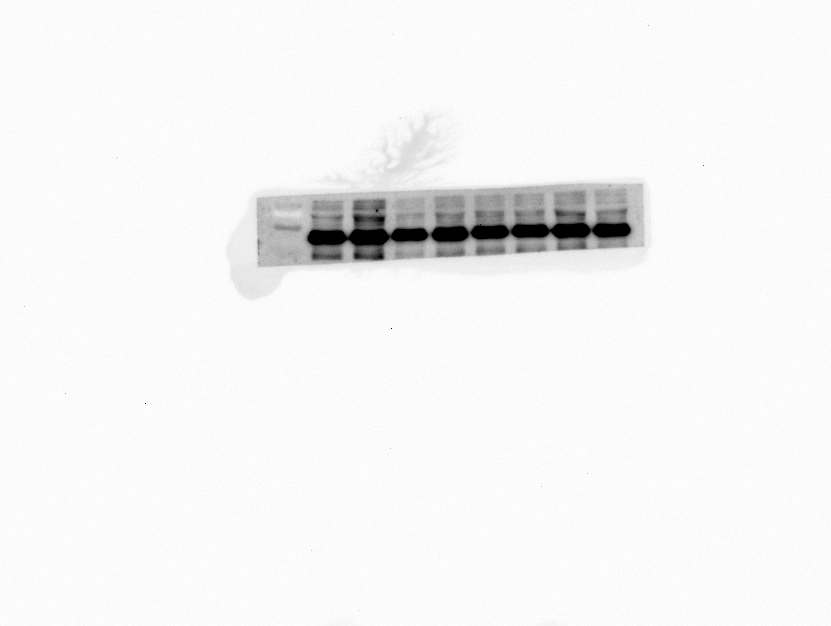

Supplement: Supplementary file 1 [file vetsci-13-00526-s001.zip › Supplementary file S1/Original Images wb/Actin/contrast/contrast_6.png]

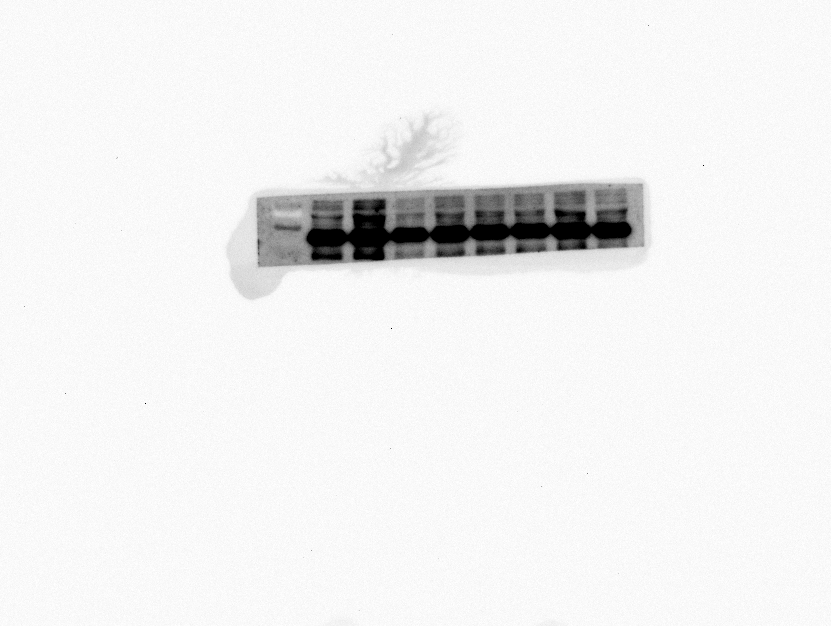

Supplement: Supplementary file 1 [file vetsci-13-00526-s001.zip › Supplementary file S1/Original Images wb/Actin/contrast/contrast_7.png]

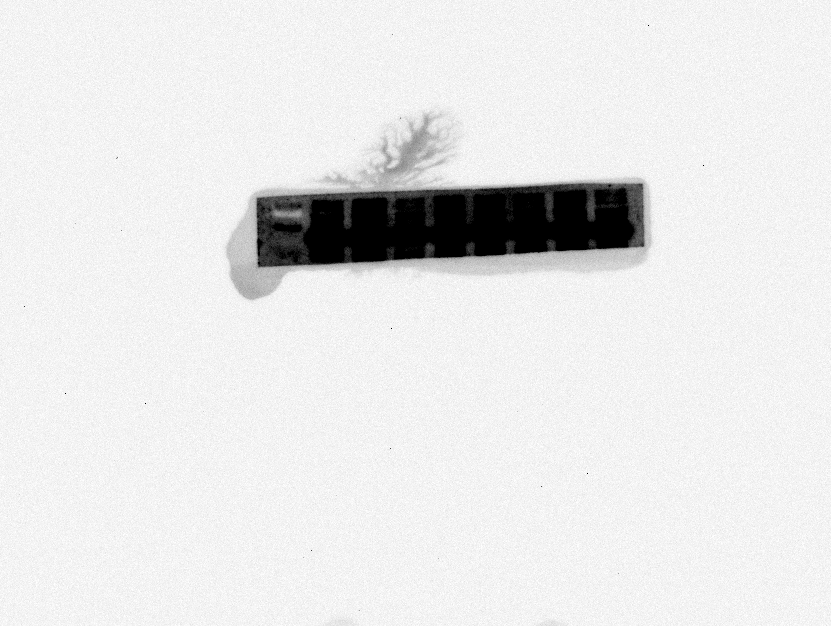

Supplement: Supplementary file 1 [file vetsci-13-00526-s001.zip › Supplementary file S1/Original Images wb/Actin/contrast/contrast_8.png]

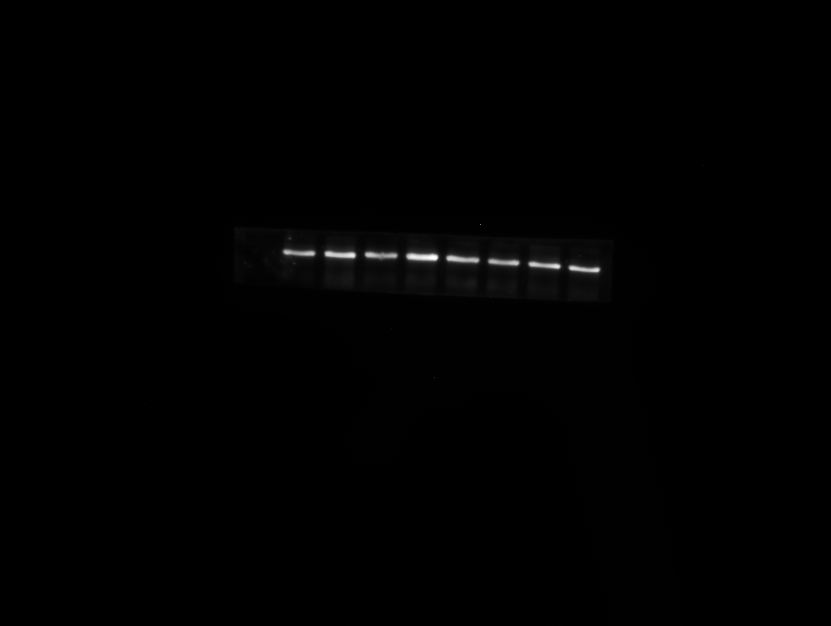

Supplement: Supplementary file 1 [file vetsci-13-00526-s001.zip › Supplementary file S1/Original Images wb/Actin-1mk/Ac-1mk2023-10-25_14-27-12_1_16bit.png]

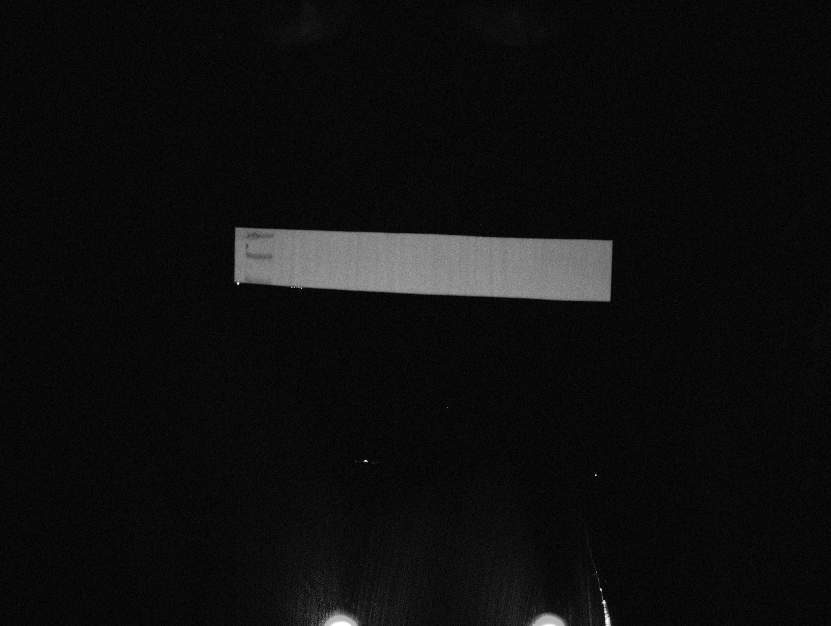

Supplement: Supplementary file 1 [file vetsci-13-00526-s001.zip › Supplementary file S1/Original Images wb/Actin-1mk/Ac-1mk2023-10-25_14-27-12_2_16bit.png]

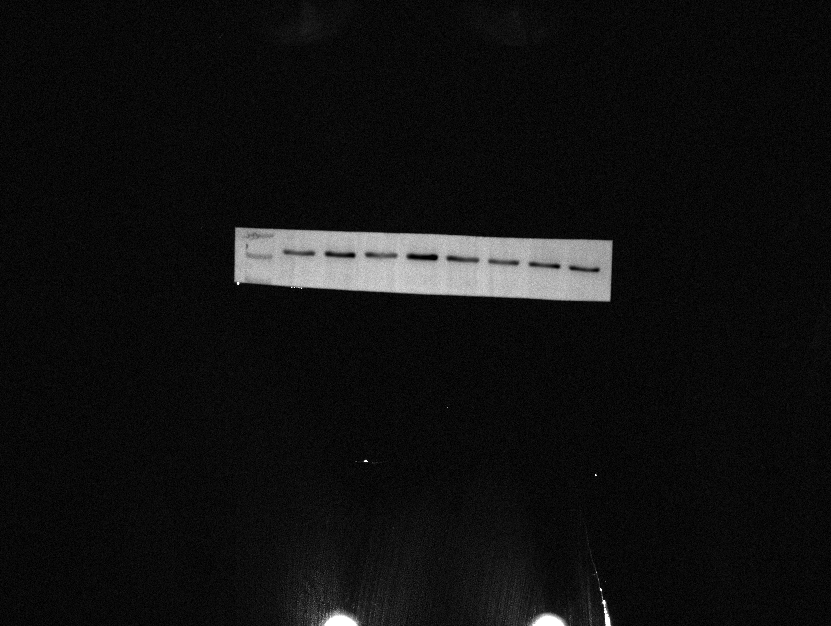

Supplement: Supplementary file 1 [file vetsci-13-00526-s001.zip › Supplementary file S1/Original Images wb/Actin-1mk/Ac-1mk2023-10-25_14-27-12_8bit.png]

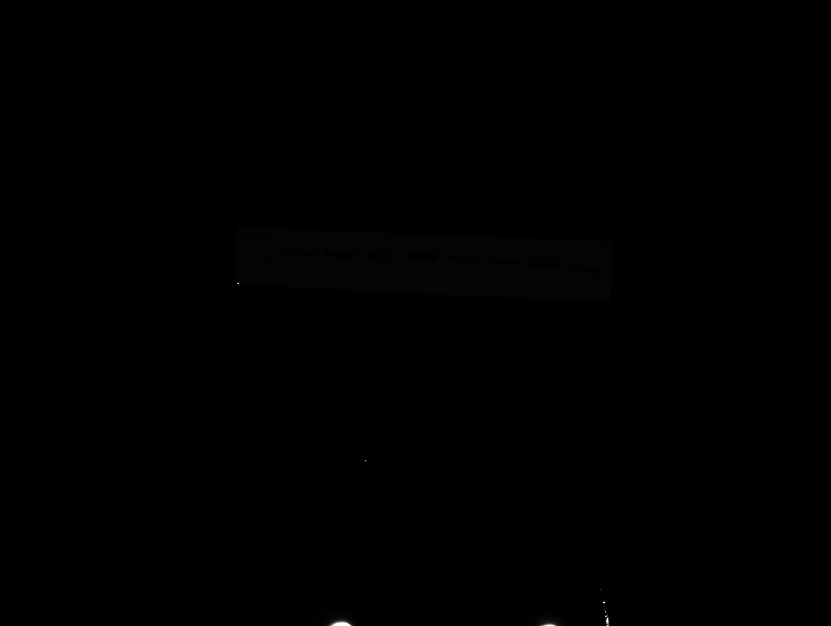

Supplement: Supplementary file 1 [file vetsci-13-00526-s001.zip › Supplementary file S1/Original Images wb/Actin-1mk/contrast/contrast_0.png]

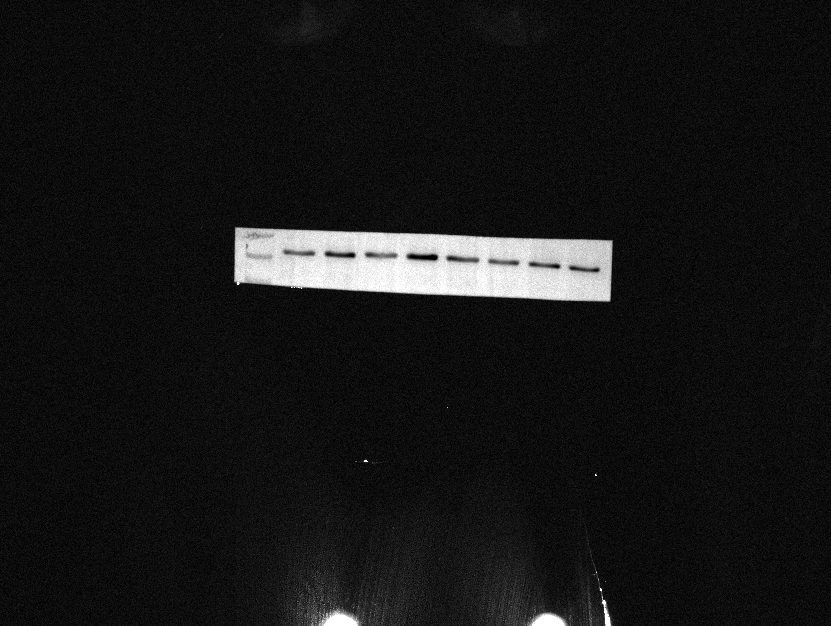

Supplement: Supplementary file 1 [file vetsci-13-00526-s001.zip › Supplementary file S1/Original Images wb/Actin-1mk/contrast/contrast_2.png]

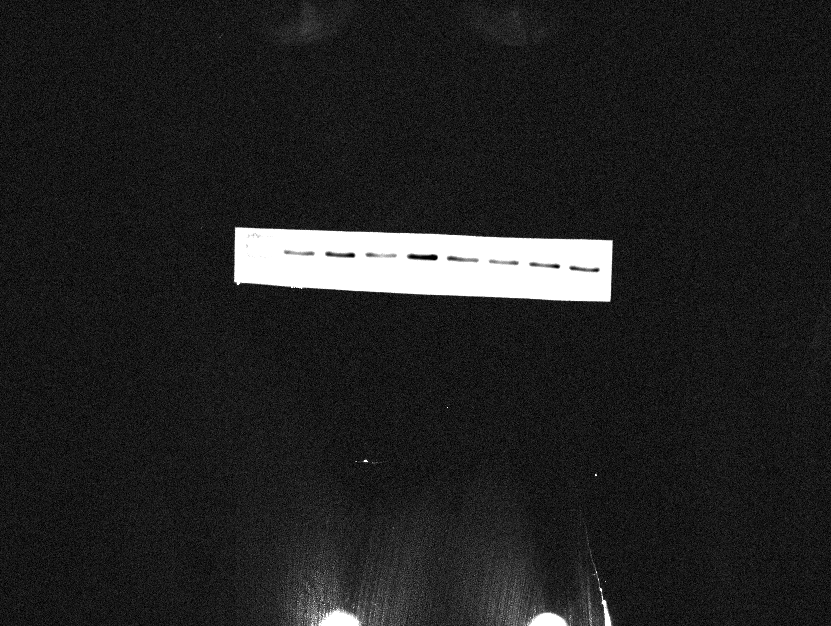

Supplement: Supplementary file 1 [file vetsci-13-00526-s001.zip › Supplementary file S1/Original Images wb/Actin-1mk/contrast/contrast_3.png]

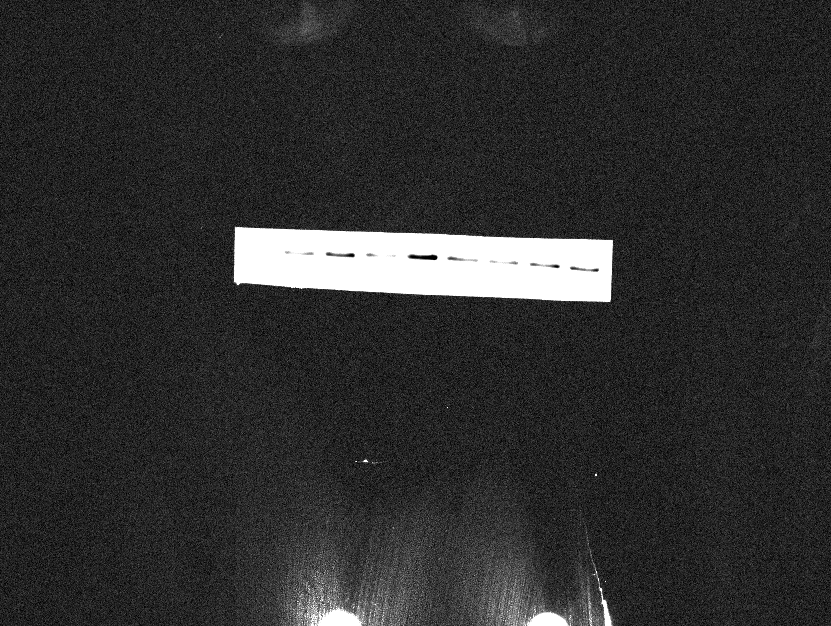

Supplement: Supplementary file 1 [file vetsci-13-00526-s001.zip › Supplementary file S1/Original Images wb/Actin-1mk/contrast/contrast_4.png]

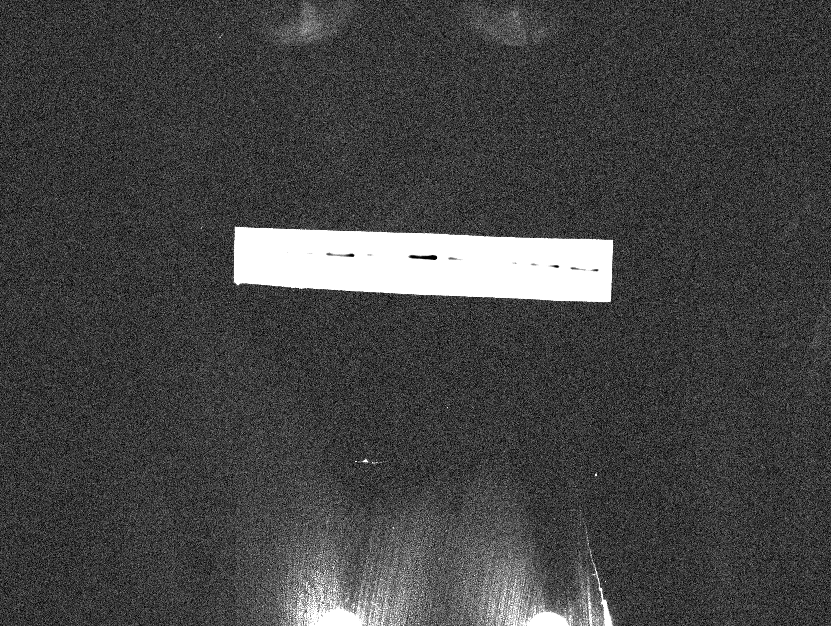

Supplement: Supplementary file 1 [file vetsci-13-00526-s001.zip › Supplementary file S1/Original Images wb/Actin-1mk/contrast/contrast_5.png]

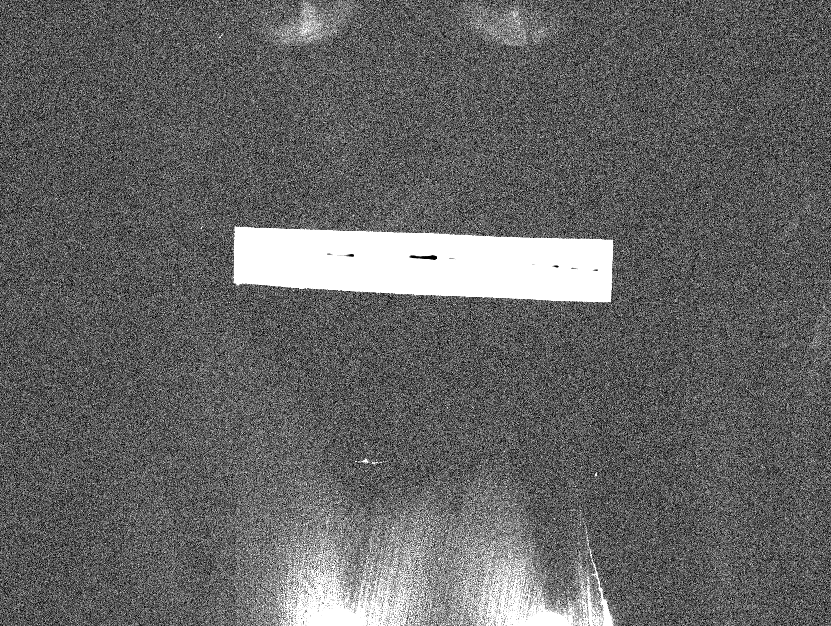

Supplement: Supplementary file 1 [file vetsci-13-00526-s001.zip › Supplementary file S1/Original Images wb/Actin-1mk/contrast/contrast_6.png]

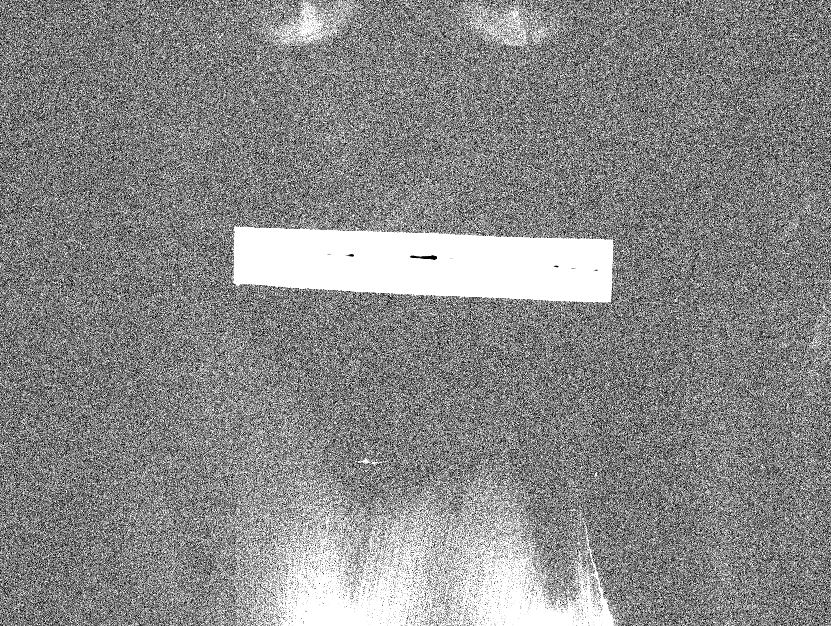

Supplement: Supplementary file 1 [file vetsci-13-00526-s001.zip › Supplementary file S1/Original Images wb/Actin-1mk/contrast/contrast_7.png]

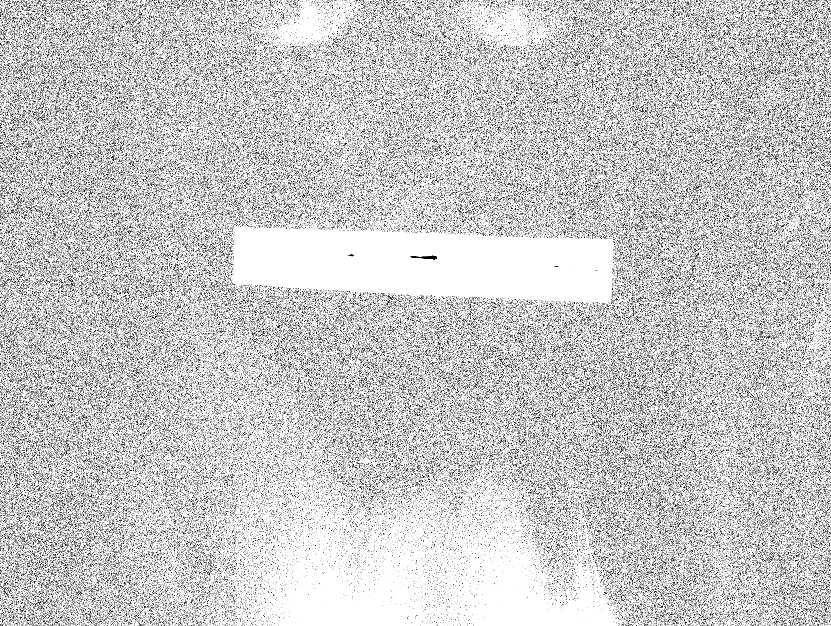

Supplement: Supplementary file 1 [file vetsci-13-00526-s001.zip › Supplementary file S1/Original Images wb/Actin-1mk/contrast/contrast_8.png]

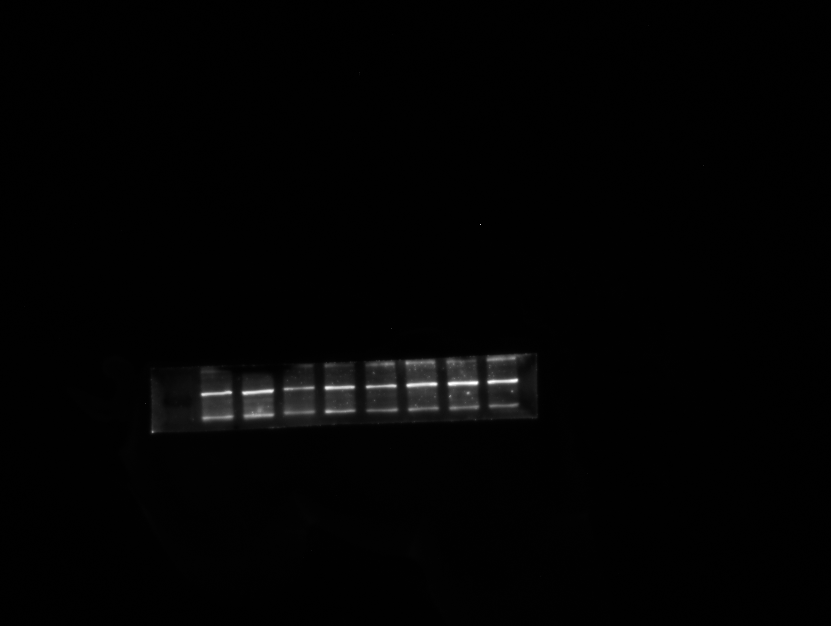

Supplement: Supplementary file 1 [file vetsci-13-00526-s001.zip › Supplementary file S1/Original Images wb/CD163-3-/163-3-2023-11-01_15-25-10_1_16bit.png]

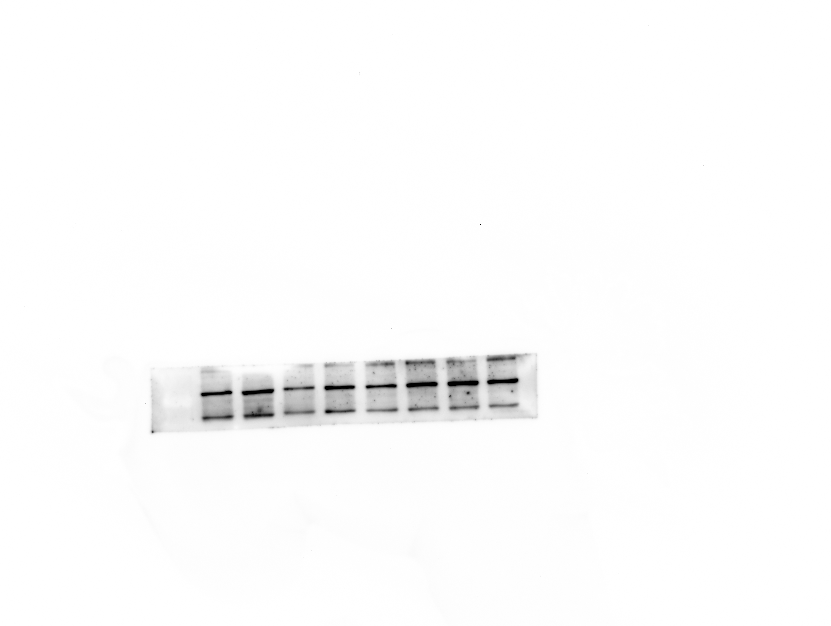

Supplement: Supplementary file 1 [file vetsci-13-00526-s001.zip › Supplementary file S1/Original Images wb/CD163-3-/163-3-2023-11-01_15-25-10_8bit.png]

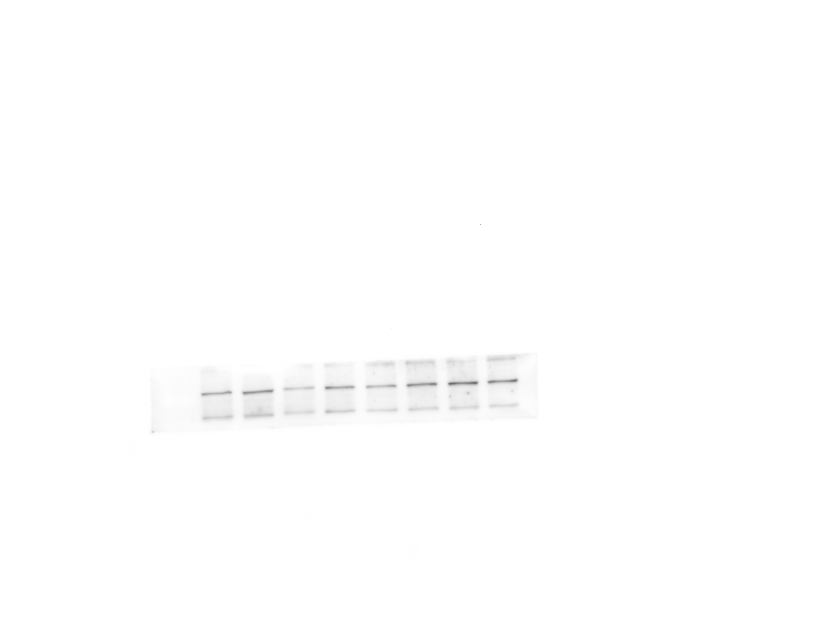

Supplement: Supplementary file 1 [file vetsci-13-00526-s001.zip › Supplementary file S1/Original Images wb/CD163-3-/contrast/contrast_0.png]

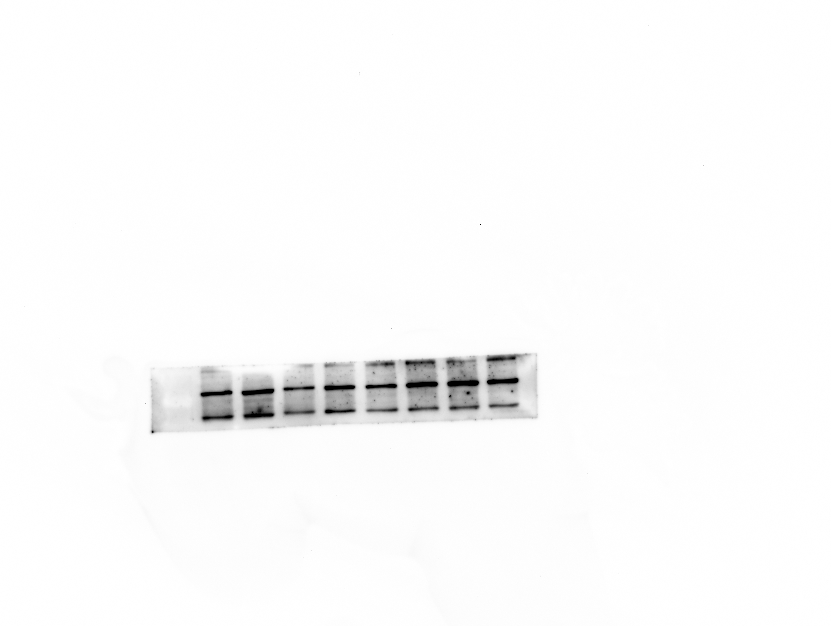

Supplement: Supplementary file 1 [file vetsci-13-00526-s001.zip › Supplementary file S1/Original Images wb/CD163-3-/contrast/contrast_2.png]

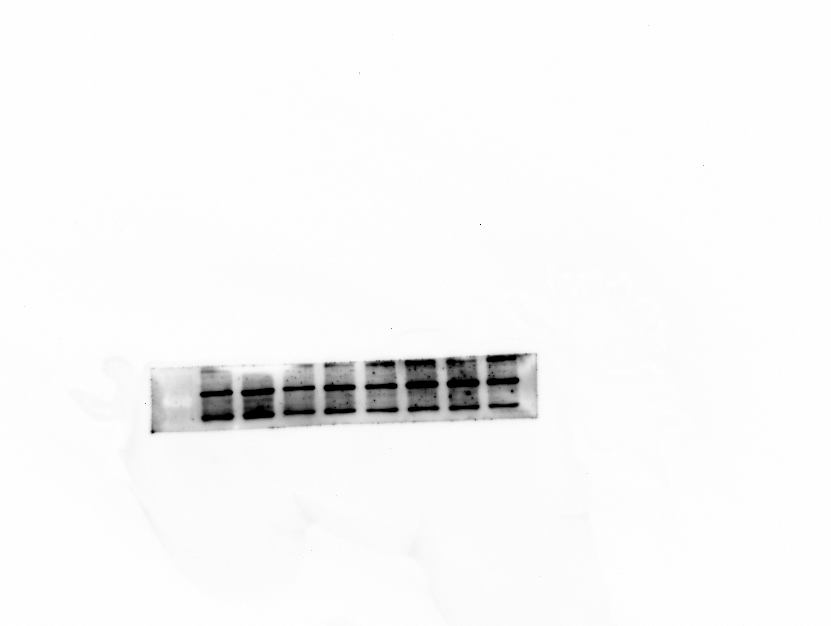

Supplement: Supplementary file 1 [file vetsci-13-00526-s001.zip › Supplementary file S1/Original Images wb/CD163-3-/contrast/contrast_3.png]

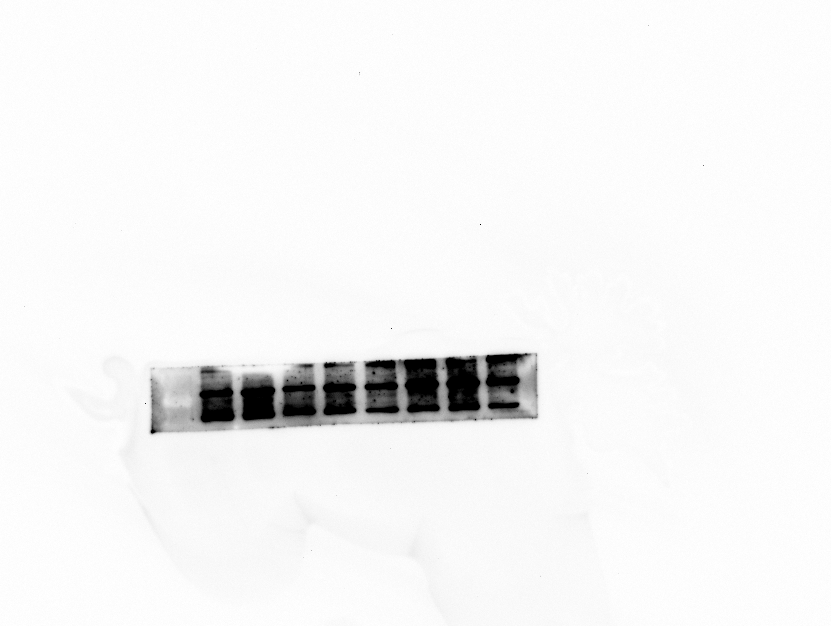

Supplement: Supplementary file 1 [file vetsci-13-00526-s001.zip › Supplementary file S1/Original Images wb/CD163-3-/contrast/contrast_4.png]

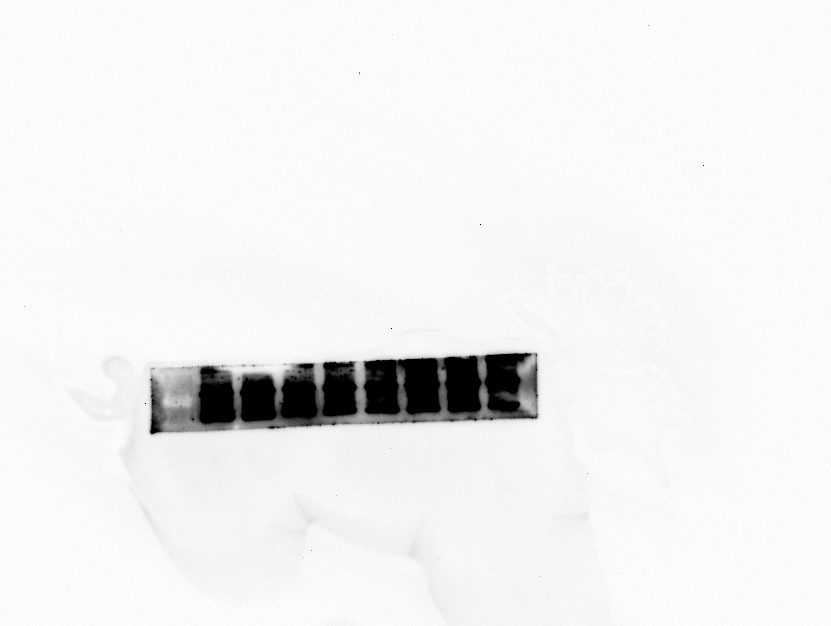

Supplement: Supplementary file 1 [file vetsci-13-00526-s001.zip › Supplementary file S1/Original Images wb/CD163-3-/contrast/contrast_5.png]

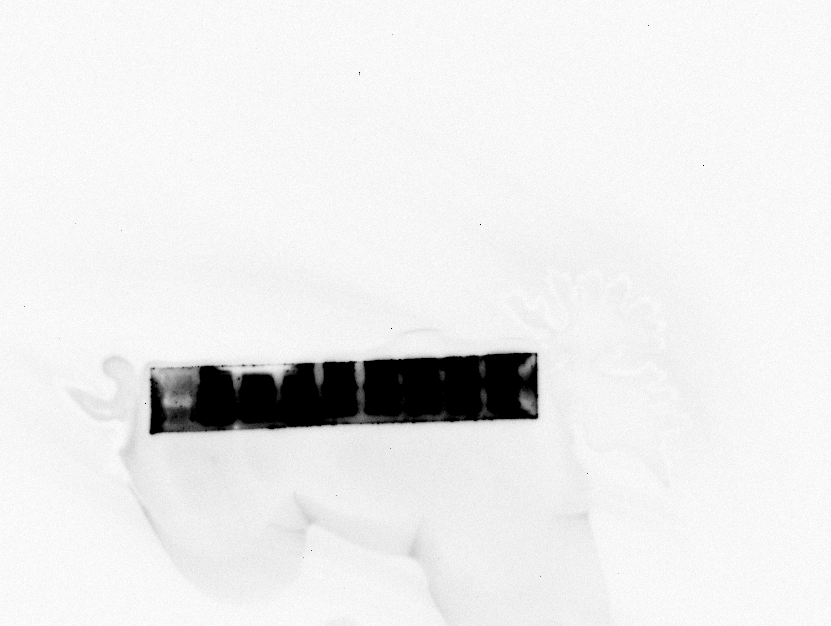

Supplement: Supplementary file 1 [file vetsci-13-00526-s001.zip › Supplementary file S1/Original Images wb/CD163-3-/contrast/contrast_6.png]

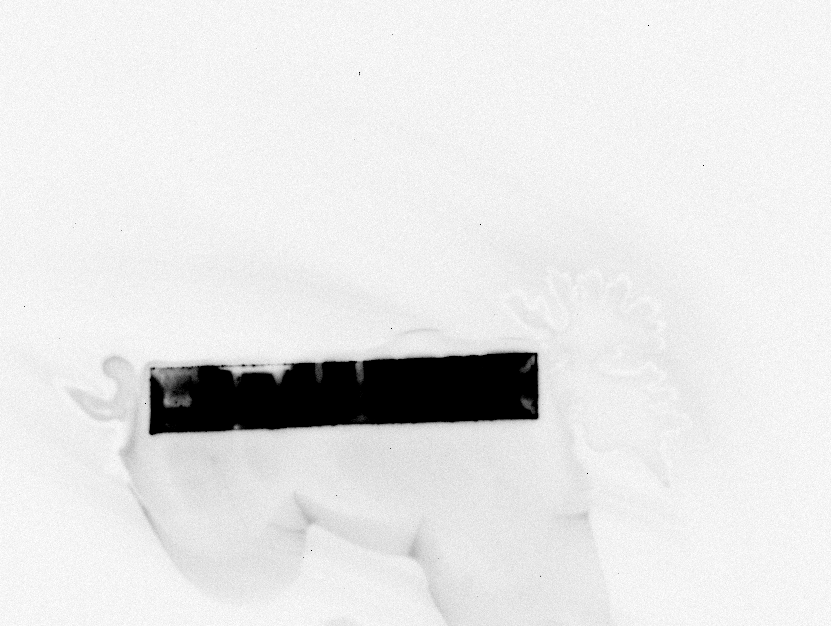

Supplement: Supplementary file 1 [file vetsci-13-00526-s001.zip › Supplementary file S1/Original Images wb/CD163-3-/contrast/contrast_7.png]

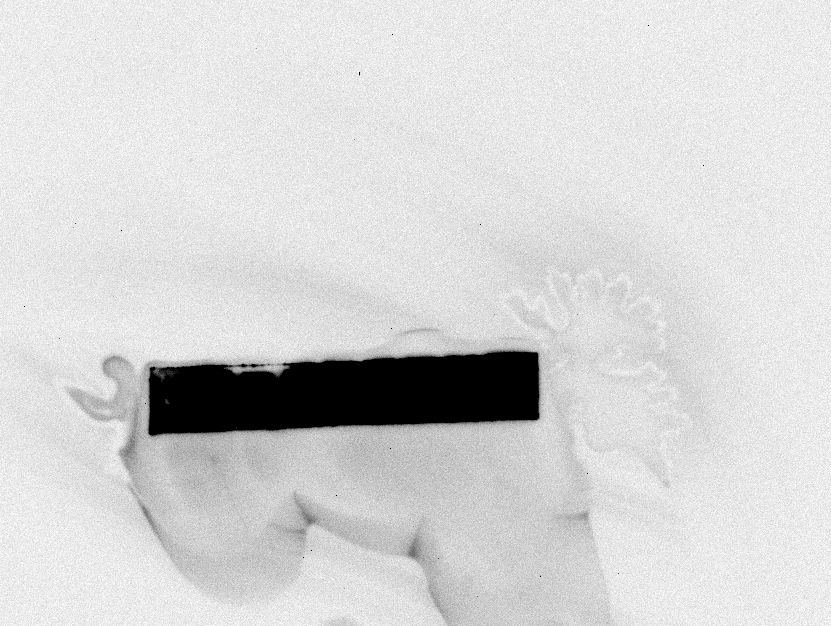

Supplement: Supplementary file 1 [file vetsci-13-00526-s001.zip › Supplementary file S1/Original Images wb/CD163-3-/contrast/contrast_8.png]

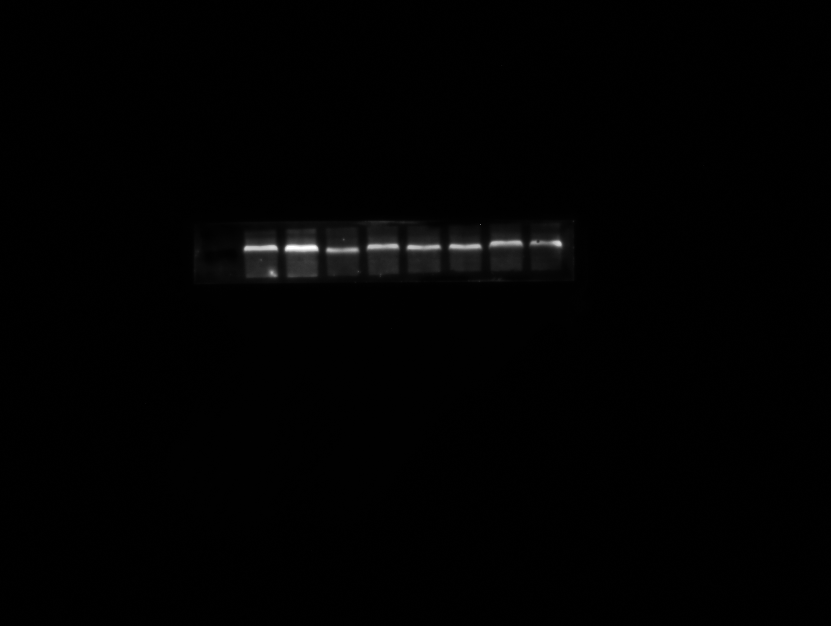

Supplement: Supplementary file 1 [file vetsci-13-00526-s001.zip › Supplementary file S1/Original Images wb/Cd86 2-/Cd2-2023-10-19_12-22-39_1_16bit.png]

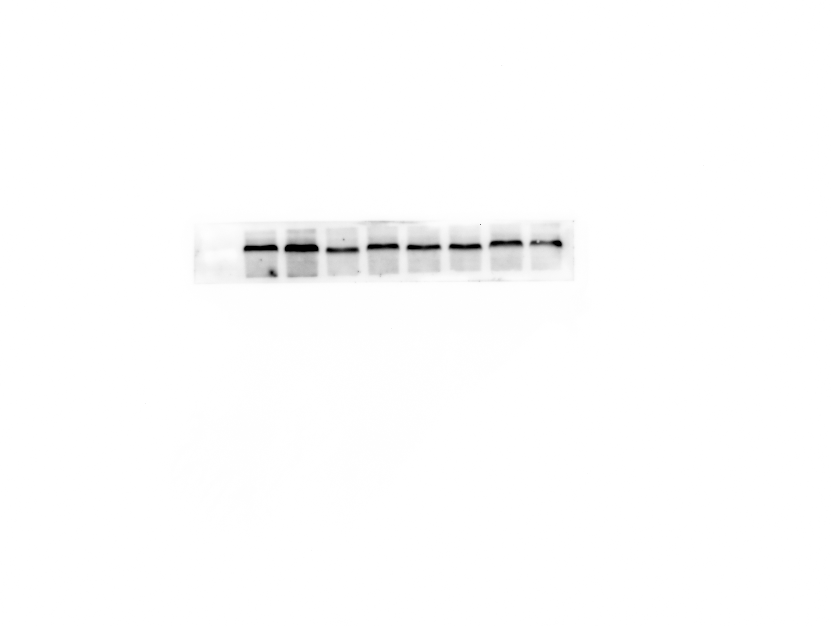

Supplement: Supplementary file 1 [file vetsci-13-00526-s001.zip › Supplementary file S1/Original Images wb/Cd86 2-/Cd2-2023-10-19_12-22-39_8bit.png]

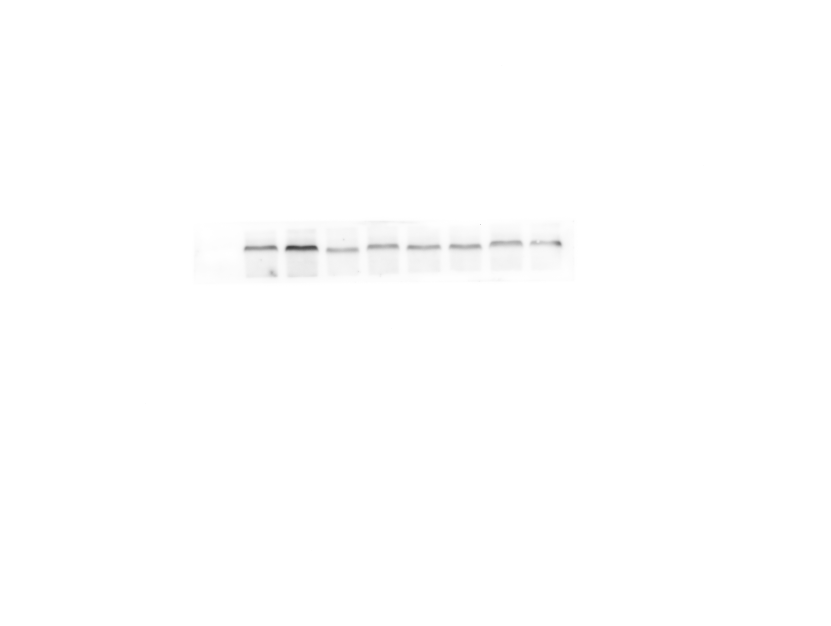

Supplement: Supplementary file 1 [file vetsci-13-00526-s001.zip › Supplementary file S1/Original Images wb/Cd86 2-/contrast/contrast_0.png]

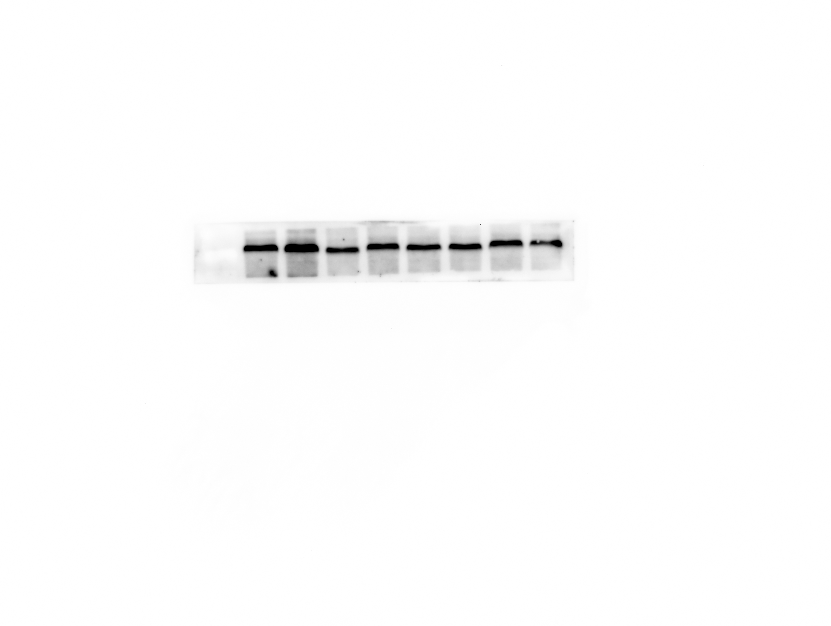

Supplement: Supplementary file 1 [file vetsci-13-00526-s001.zip › Supplementary file S1/Original Images wb/Cd86 2-/contrast/contrast_2.png]

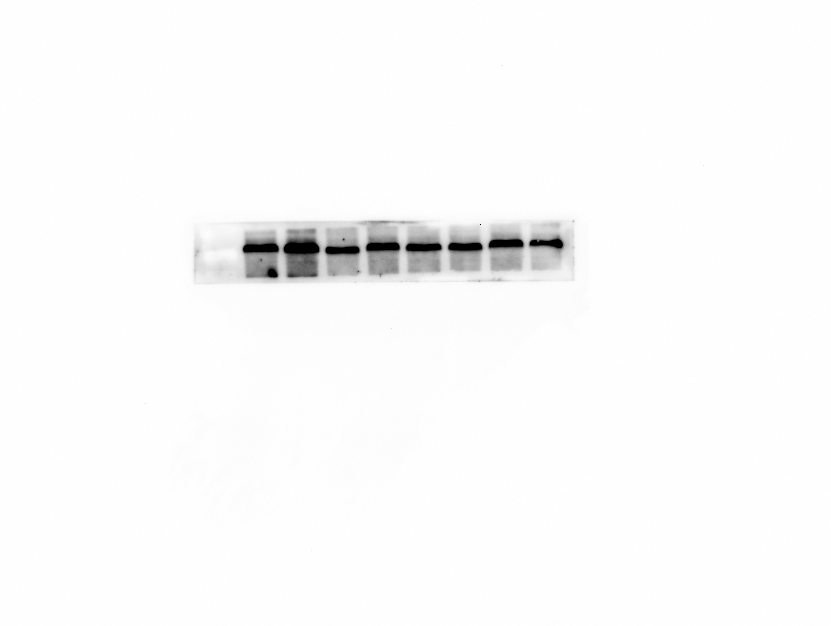

Supplement: Supplementary file 1 [file vetsci-13-00526-s001.zip › Supplementary file S1/Original Images wb/Cd86 2-/contrast/contrast_3.png]

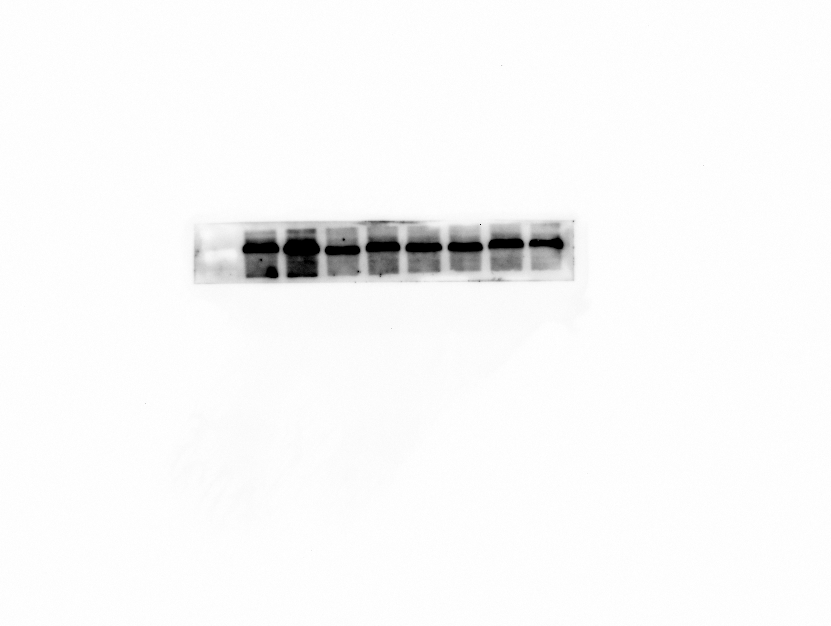

Supplement: Supplementary file 1 [file vetsci-13-00526-s001.zip › Supplementary file S1/Original Images wb/Cd86 2-/contrast/contrast_4.png]

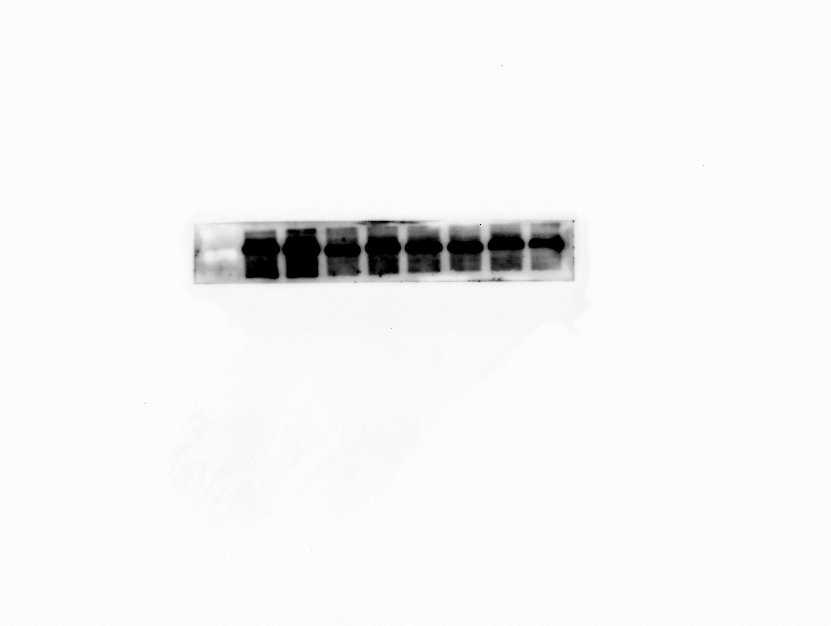

Supplement: Supplementary file 1 [file vetsci-13-00526-s001.zip › Supplementary file S1/Original Images wb/Cd86 2-/contrast/contrast_5.png]

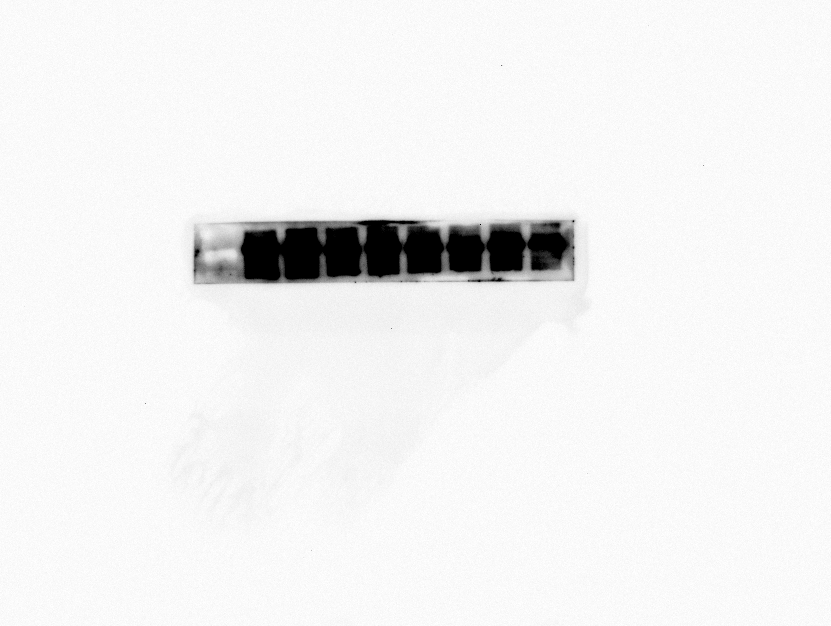

Supplement: Supplementary file 1 [file vetsci-13-00526-s001.zip › Supplementary file S1/Original Images wb/Cd86 2-/contrast/contrast_6.png]

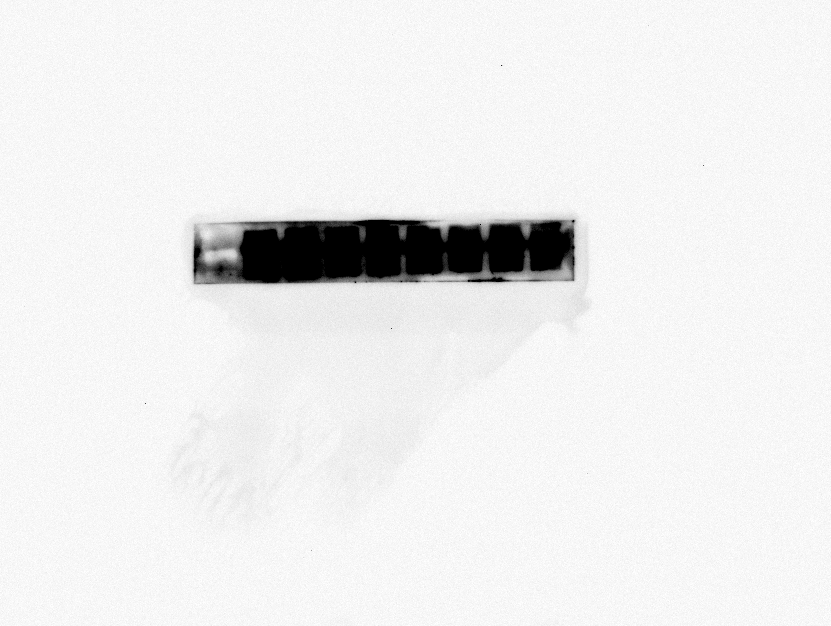

Supplement: Supplementary file 1 [file vetsci-13-00526-s001.zip › Supplementary file S1/Original Images wb/Cd86 2-/contrast/contrast_7.png]

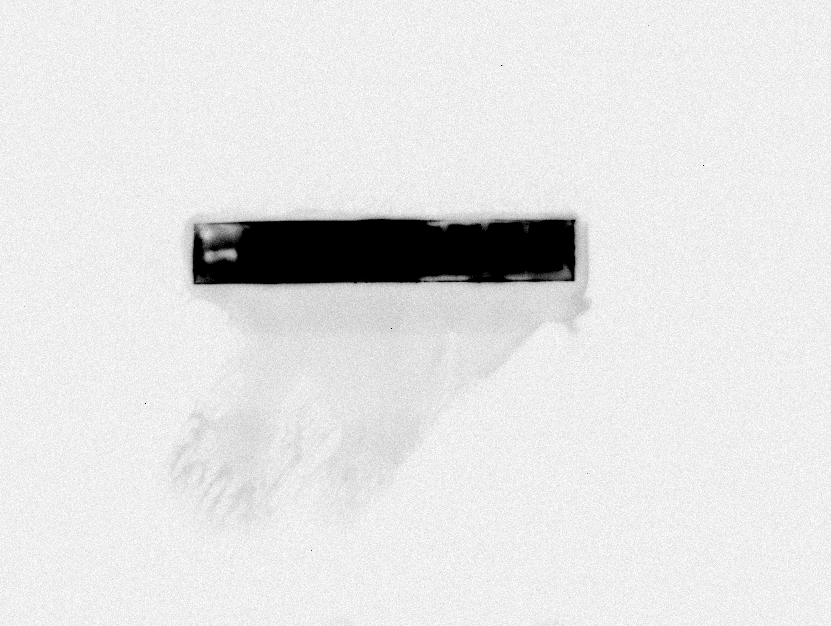

Supplement: Supplementary file 1 [file vetsci-13-00526-s001.zip › Supplementary file S1/Original Images wb/Cd86 2-/contrast/contrast_8.png]

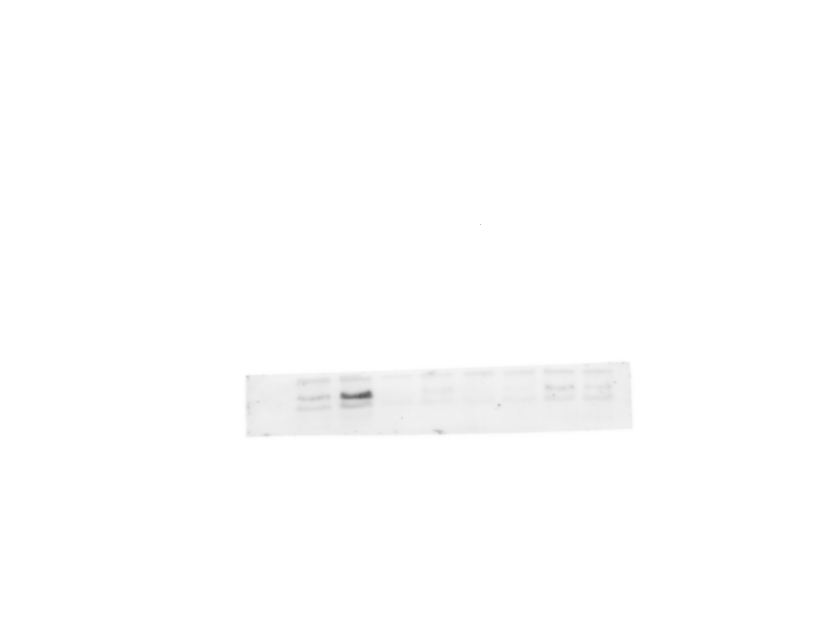

Supplement: Supplementary file 1 [file vetsci-13-00526-s001.zip › Supplementary file S1/Original Images wb/IL1β-1/contrast/contrast_0.png]

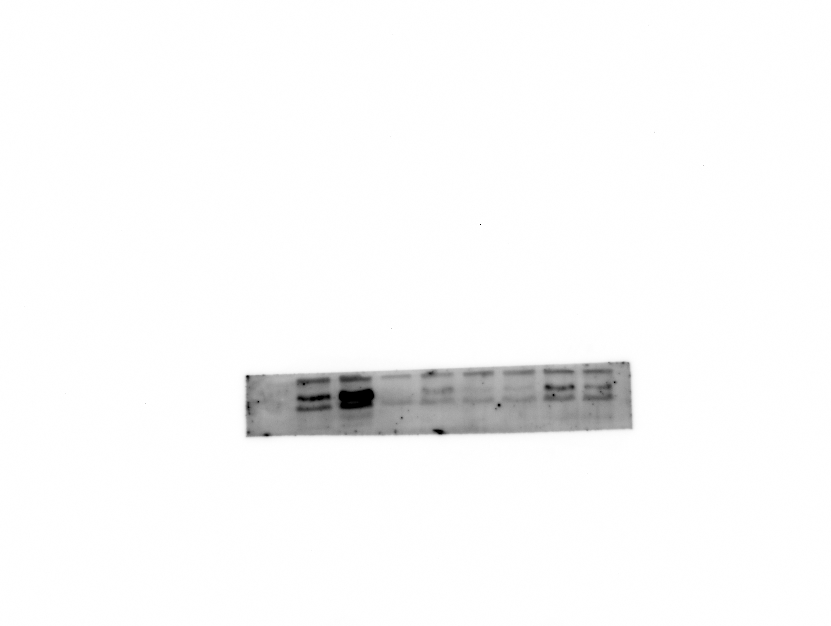

Supplement: Supplementary file 1 [file vetsci-13-00526-s001.zip › Supplementary file S1/Original Images wb/IL1β-1/contrast/contrast_1.png]

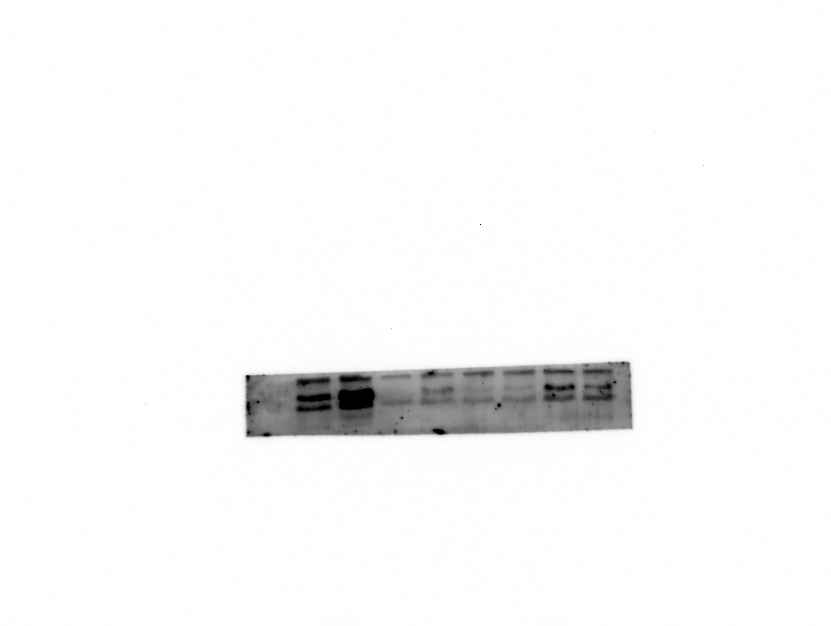

Supplement: Supplementary file 1 [file vetsci-13-00526-s001.zip › Supplementary file S1/Original Images wb/IL1β-1/contrast/contrast_2.png]

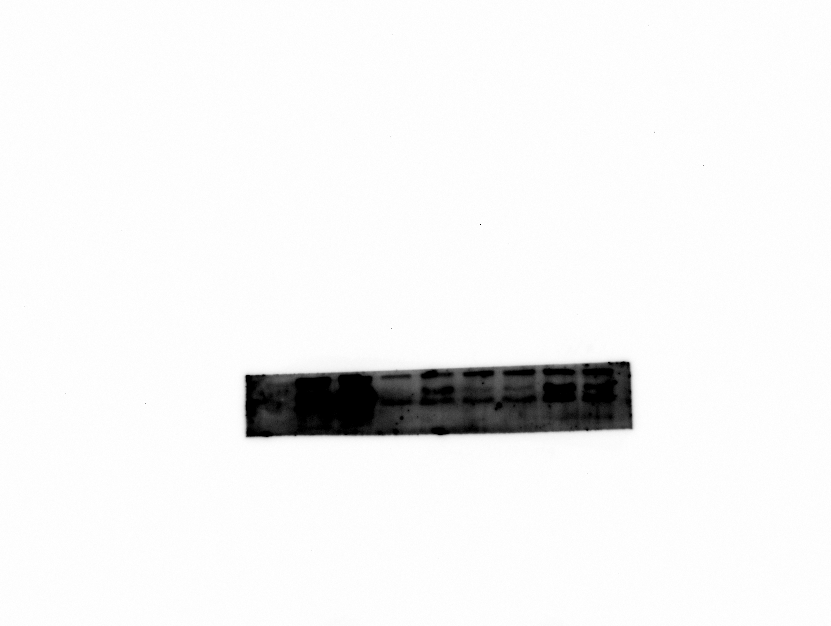

Supplement: Supplementary file 1 [file vetsci-13-00526-s001.zip › Supplementary file S1/Original Images wb/IL1β-1/contrast/contrast_3.png]

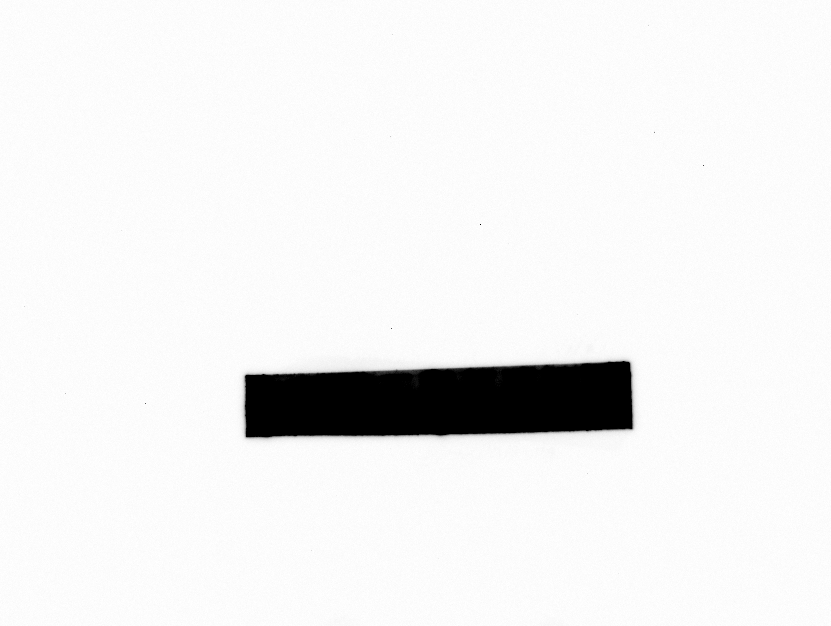

Supplement: Supplementary file 1 [file vetsci-13-00526-s001.zip › Supplementary file S1/Original Images wb/IL1β-1/contrast/contrast_4.png]

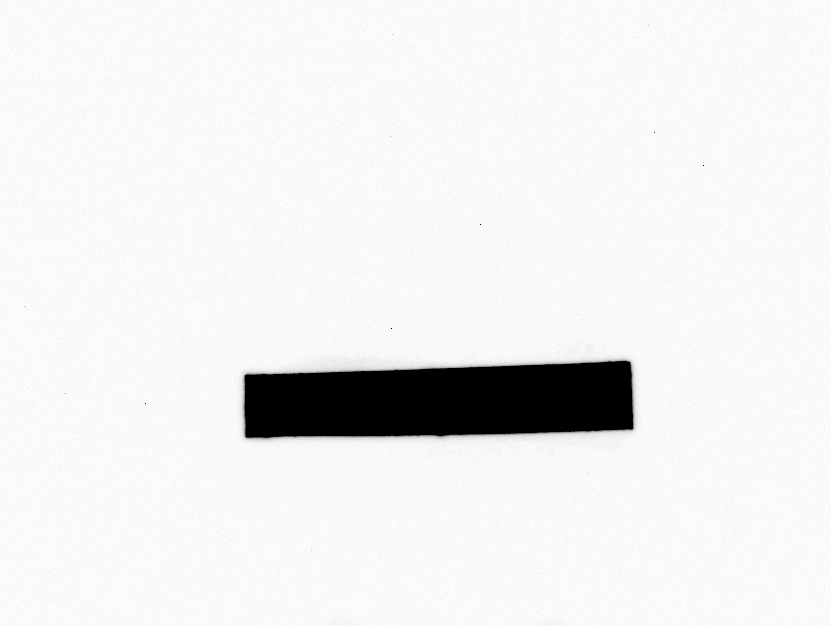

Supplement: Supplementary file 1 [file vetsci-13-00526-s001.zip › Supplementary file S1/Original Images wb/IL1β-1/contrast/contrast_5.png]

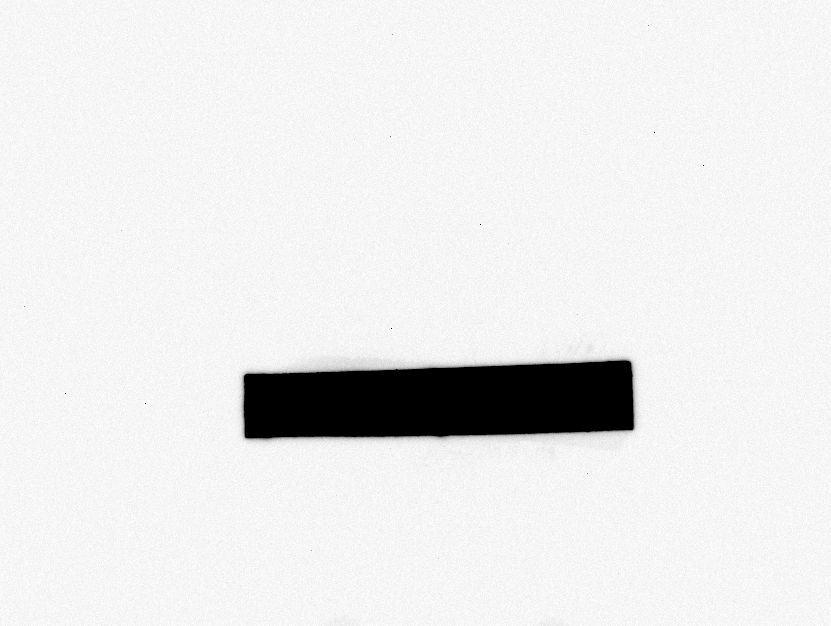

Supplement: Supplementary file 1 [file vetsci-13-00526-s001.zip › Supplementary file S1/Original Images wb/IL1β-1/contrast/contrast_6.png]

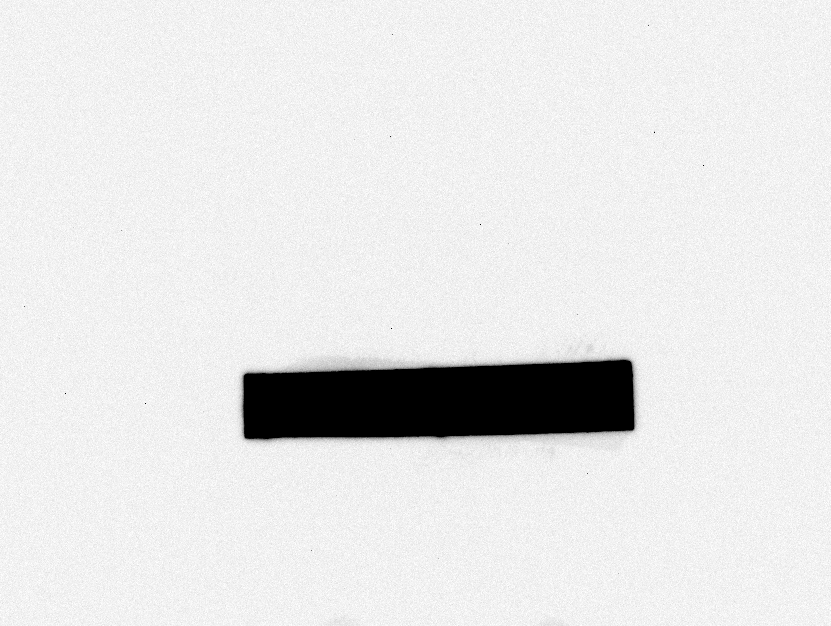

Supplement: Supplementary file 1 [file vetsci-13-00526-s001.zip › Supplementary file S1/Original Images wb/IL1β-1/contrast/contrast_7.png]

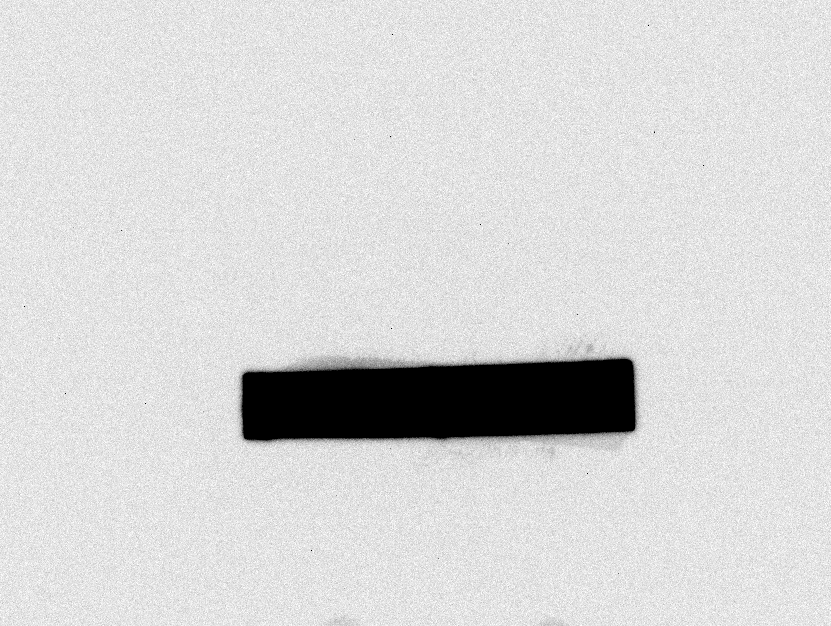

Supplement: Supplementary file 1 [file vetsci-13-00526-s001.zip › Supplementary file S1/Original Images wb/IL1β-1/contrast/contrast_8.png]

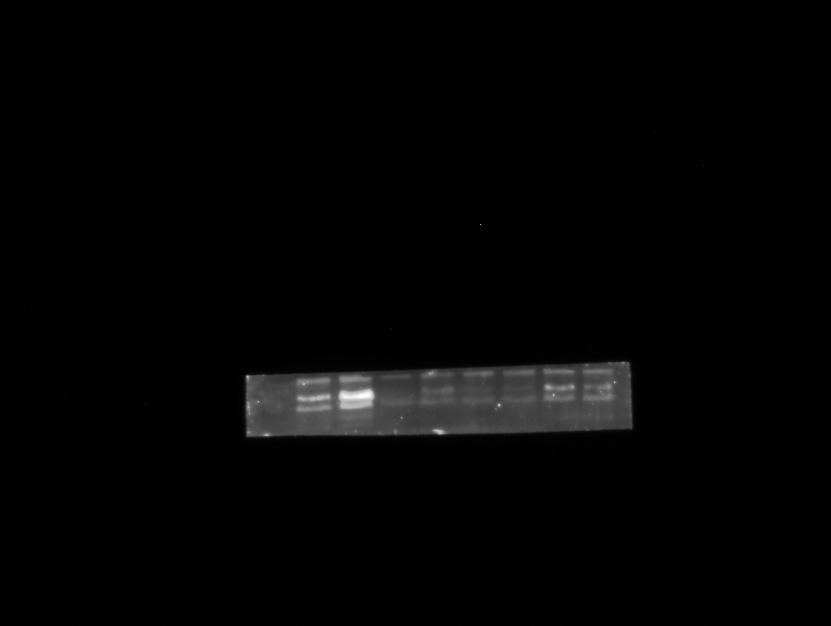

Supplement: Supplementary file 1 [file vetsci-13-00526-s001.zip › Supplementary file S1/Original Images wb/IL1β-1/Lb-1-2023-12-08_15-10-09_1_16bit.png]

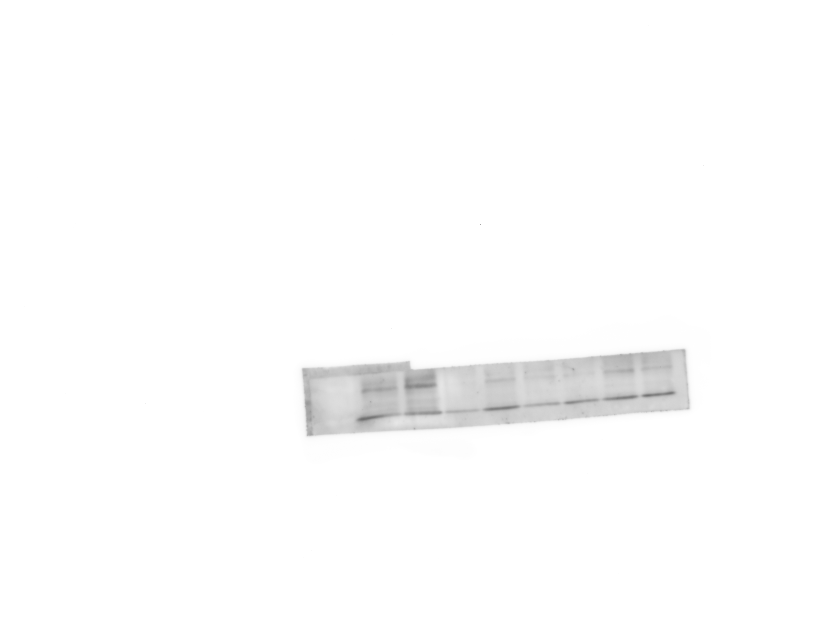

Supplement: Supplementary file 1 [file vetsci-13-00526-s001.zip › Supplementary file S1/Original Images wb/IL6/contrast/contrast_0.png]

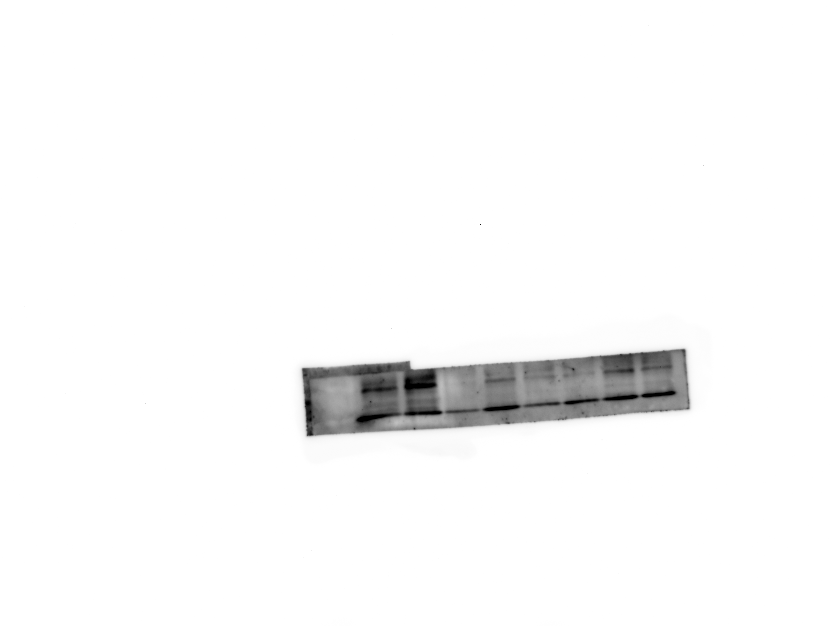

Supplement: Supplementary file 1 [file vetsci-13-00526-s001.zip › Supplementary file S1/Original Images wb/IL6/contrast/contrast_1.png]

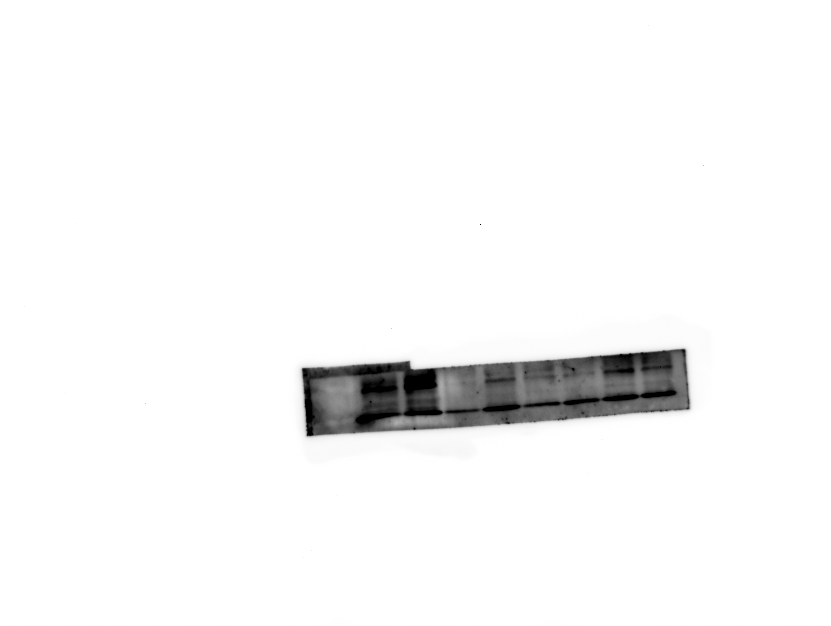

Supplement: Supplementary file 1 [file vetsci-13-00526-s001.zip › Supplementary file S1/Original Images wb/IL6/contrast/contrast_2.png]

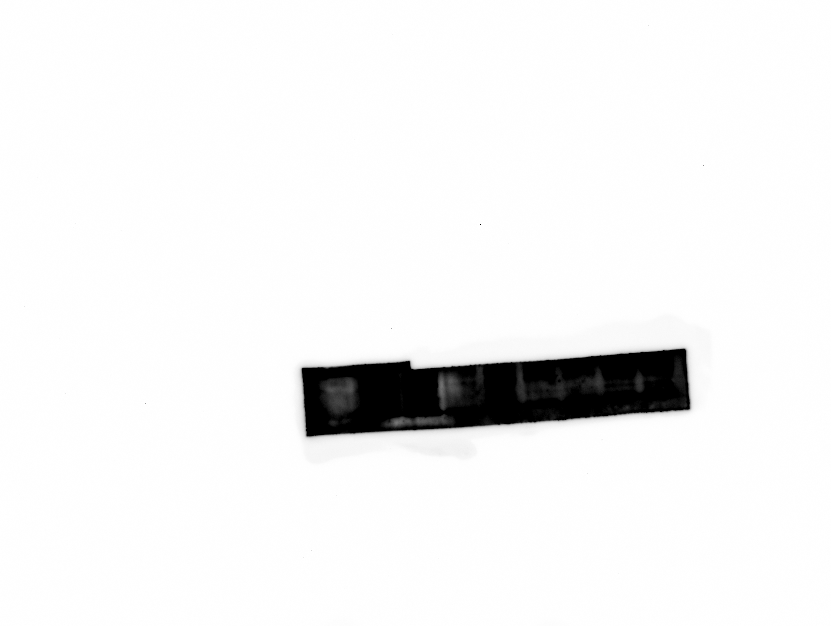

Supplement: Supplementary file 1 [file vetsci-13-00526-s001.zip › Supplementary file S1/Original Images wb/IL6/contrast/contrast_3.png]

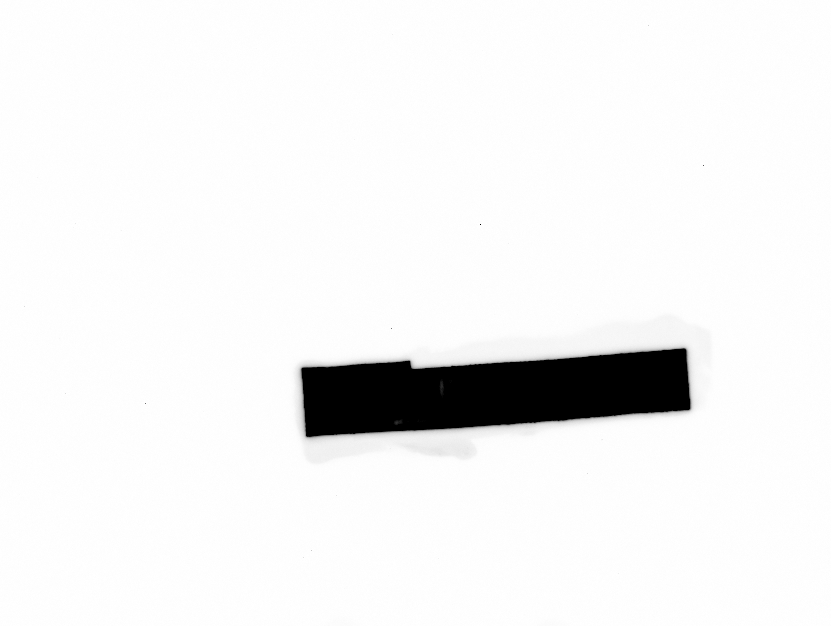

Supplement: Supplementary file 1 [file vetsci-13-00526-s001.zip › Supplementary file S1/Original Images wb/IL6/contrast/contrast_4.png]

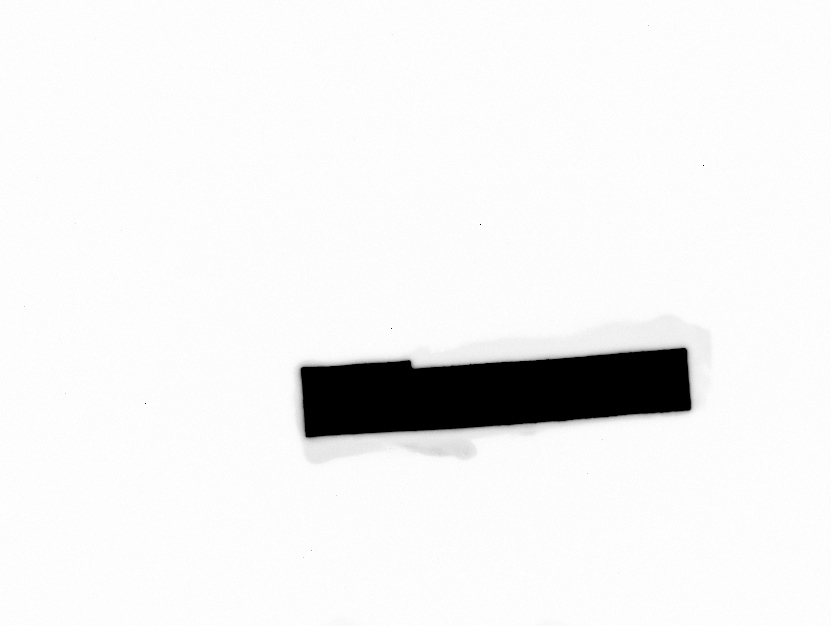

Supplement: Supplementary file 1 [file vetsci-13-00526-s001.zip › Supplementary file S1/Original Images wb/IL6/contrast/contrast_5.png]

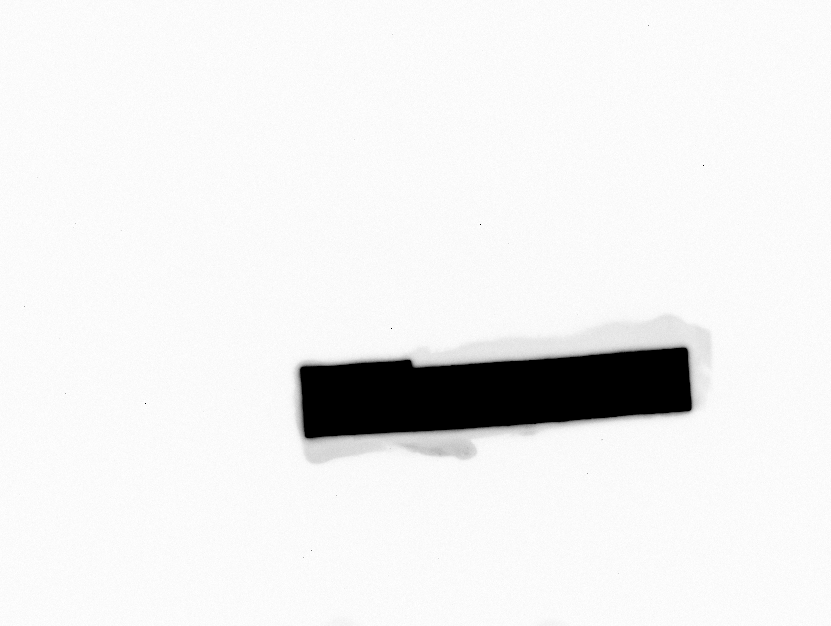

Supplement: Supplementary file 1 [file vetsci-13-00526-s001.zip › Supplementary file S1/Original Images wb/IL6/contrast/contrast_6.png]

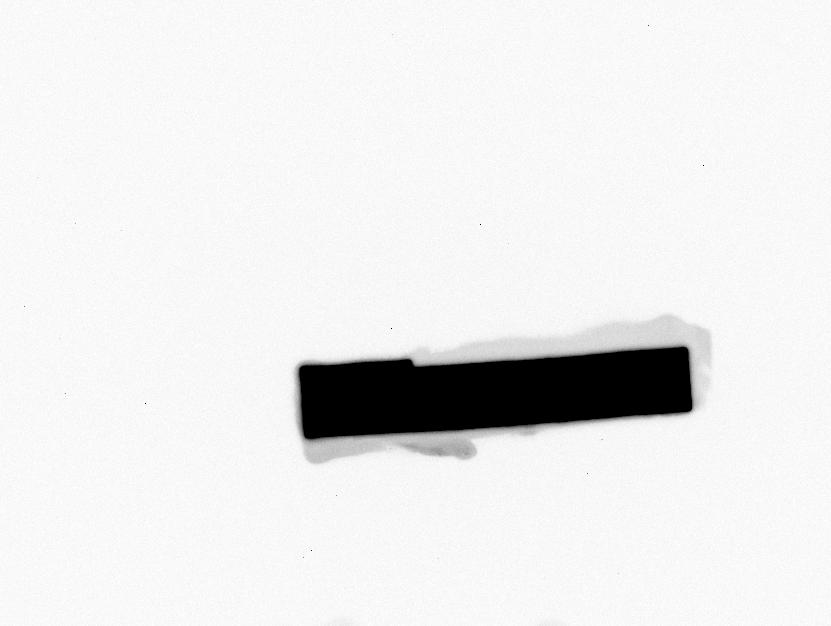

Supplement: Supplementary file 1 [file vetsci-13-00526-s001.zip › Supplementary file S1/Original Images wb/IL6/contrast/contrast_7.png]

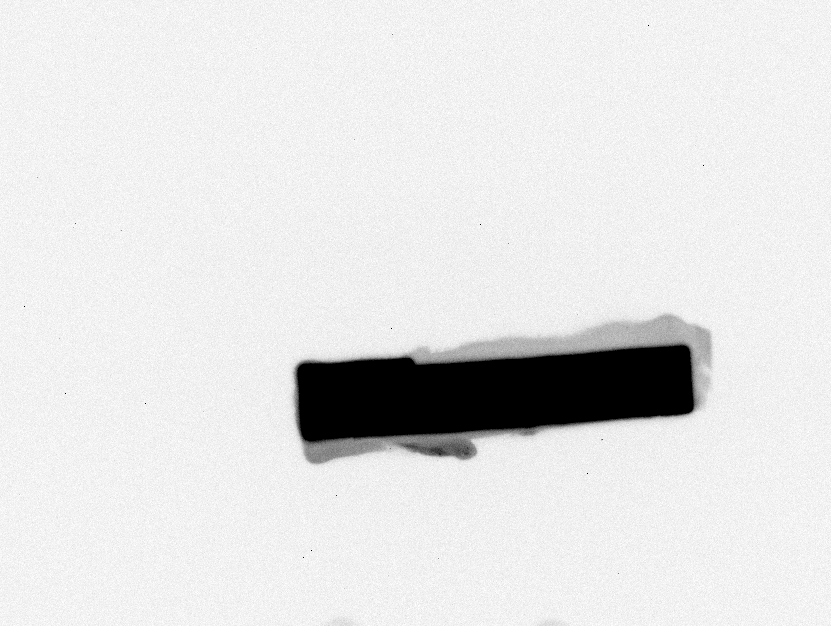

Supplement: Supplementary file 1 [file vetsci-13-00526-s001.zip › Supplementary file S1/Original Images wb/IL6/contrast/contrast_8.png]

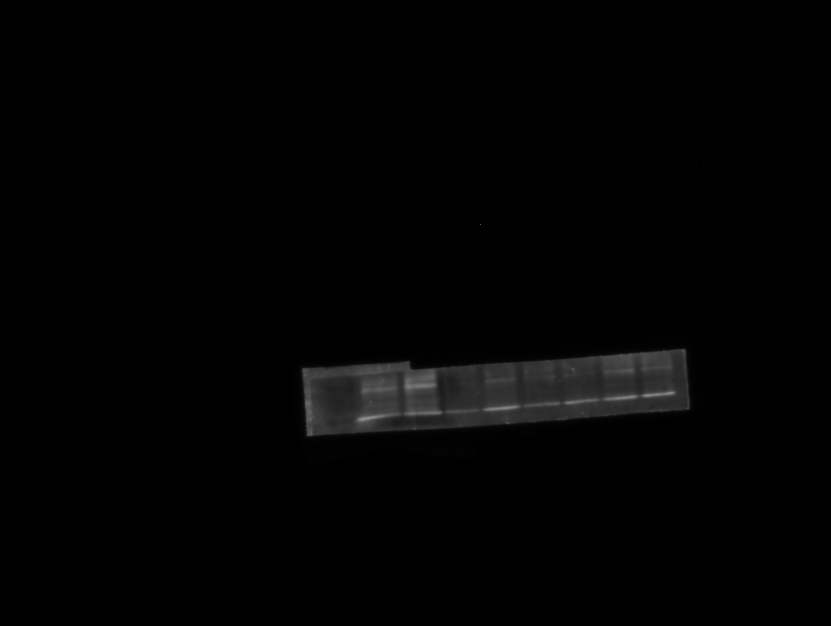

Supplement: Supplementary file 1 [file vetsci-13-00526-s001.zip › Supplementary file S1/Original Images wb/IL6/Il6-2023-12-28_10-05-46_1_16bit.png]

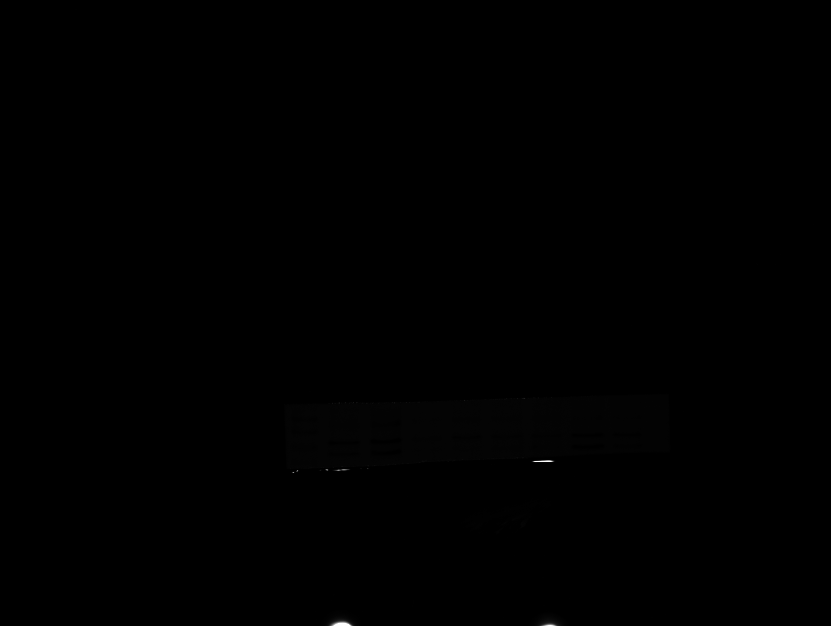

Supplement: Supplementary file 1 [file vetsci-13-00526-s001.zip › Supplementary file S1/Original Images wb/My-3mk/contrast/contrast_0.png]

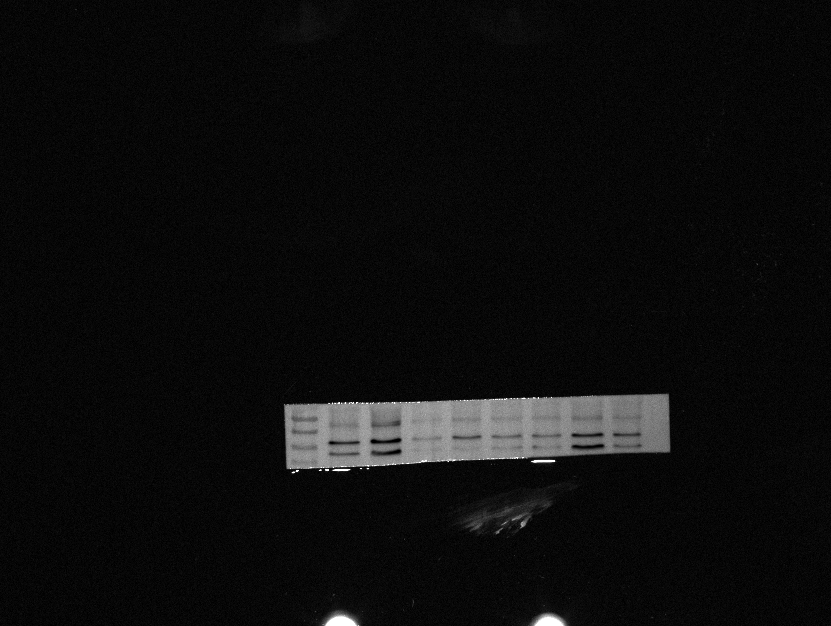

Supplement: Supplementary file 1 [file vetsci-13-00526-s001.zip › Supplementary file S1/Original Images wb/My-3mk/contrast/contrast_1.png]

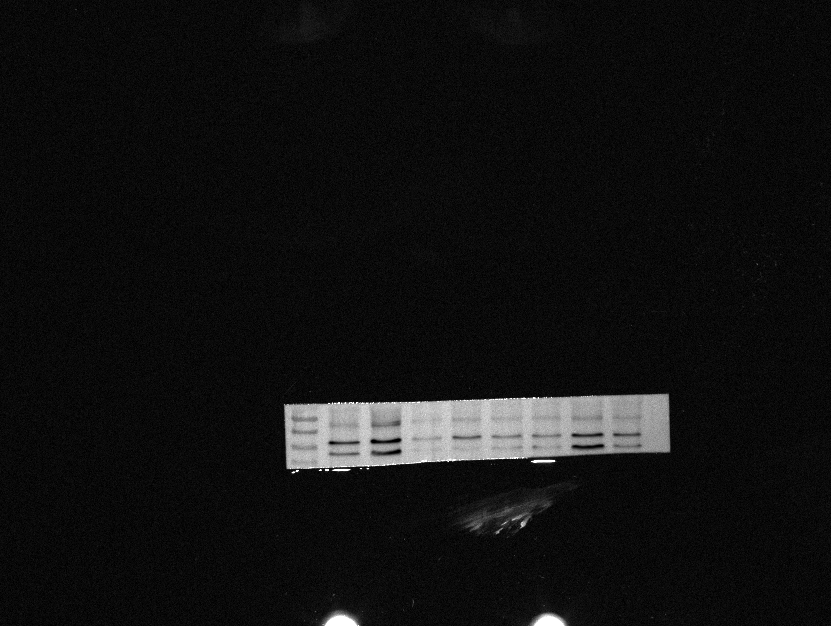

Supplement: Supplementary file 1 [file vetsci-13-00526-s001.zip › Supplementary file S1/Original Images wb/My-3mk/contrast/contrast_2.png]

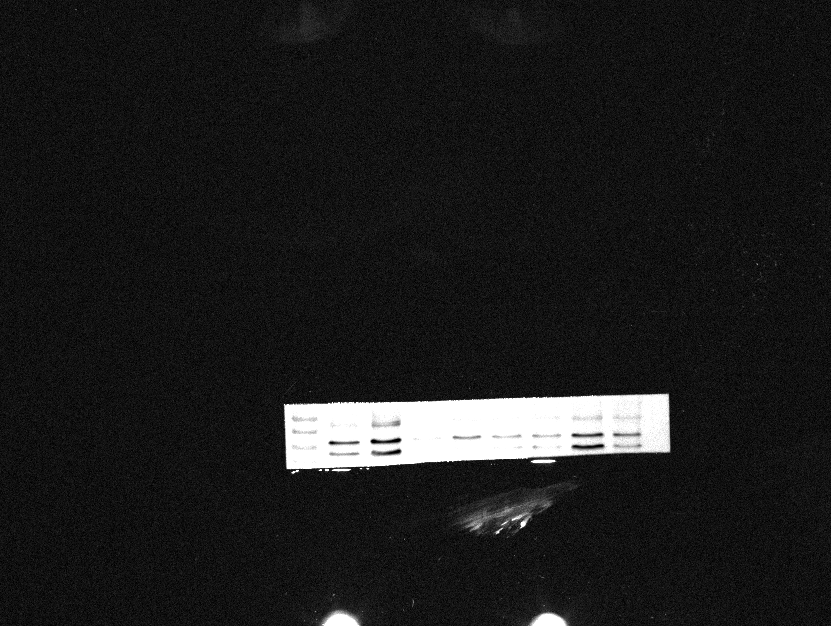

Supplement: Supplementary file 1 [file vetsci-13-00526-s001.zip › Supplementary file S1/Original Images wb/My-3mk/contrast/contrast_3.png]

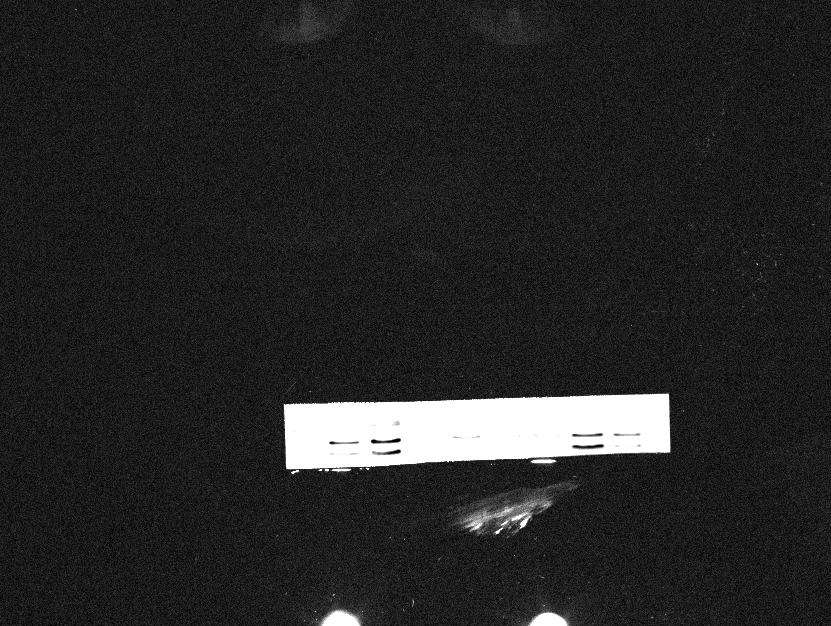

Supplement: Supplementary file 1 [file vetsci-13-00526-s001.zip › Supplementary file S1/Original Images wb/My-3mk/contrast/contrast_4.png]

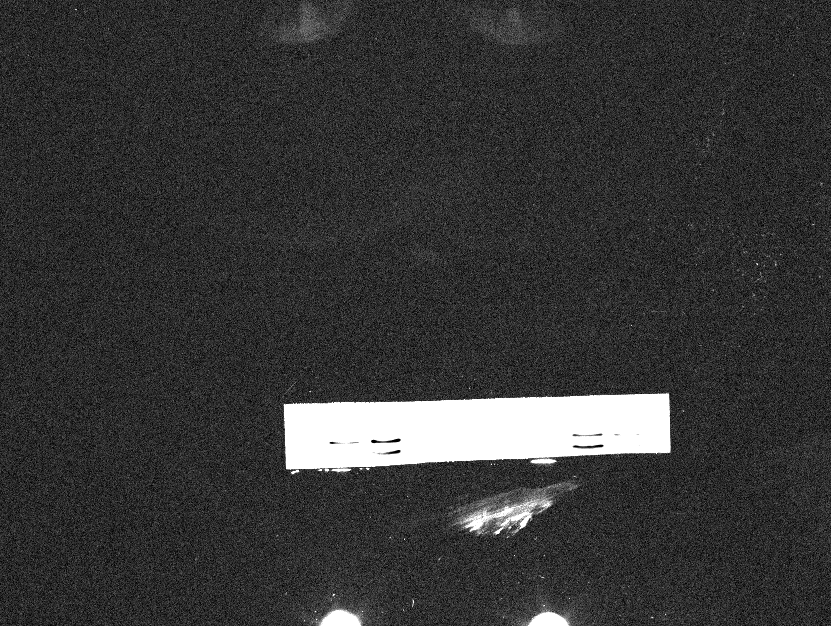

Supplement: Supplementary file 1 [file vetsci-13-00526-s001.zip › Supplementary file S1/Original Images wb/My-3mk/contrast/contrast_5.png]

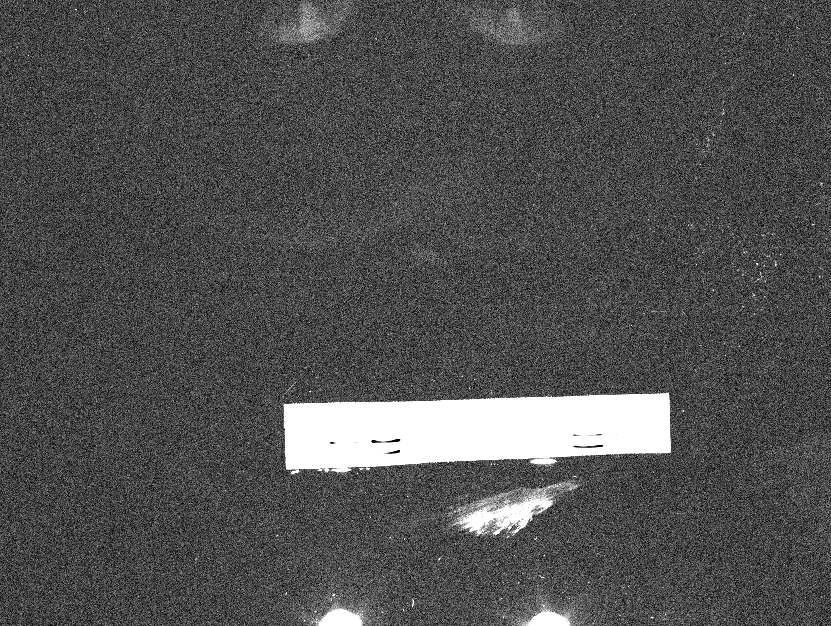

Supplement: Supplementary file 1 [file vetsci-13-00526-s001.zip › Supplementary file S1/Original Images wb/My-3mk/contrast/contrast_6.png]

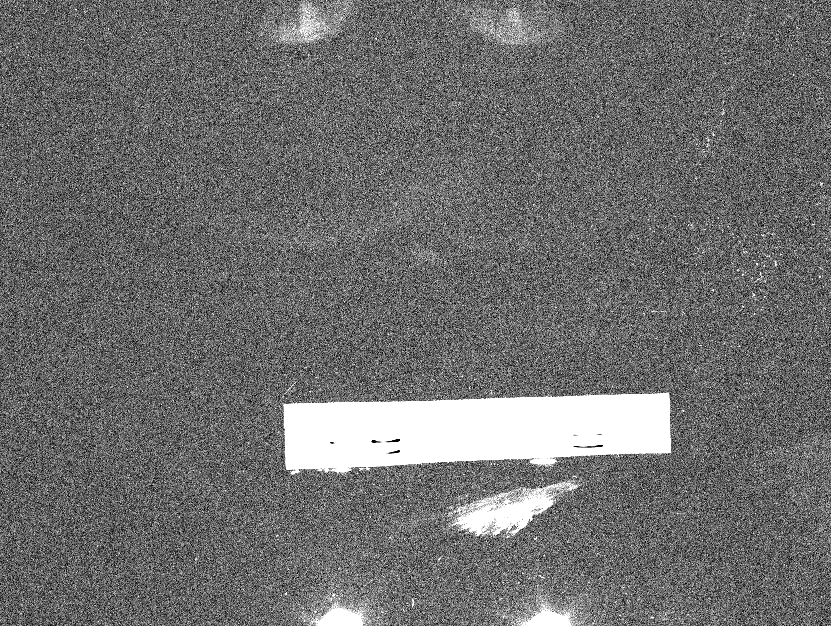

Supplement: Supplementary file 1 [file vetsci-13-00526-s001.zip › Supplementary file S1/Original Images wb/My-3mk/contrast/contrast_7.png]

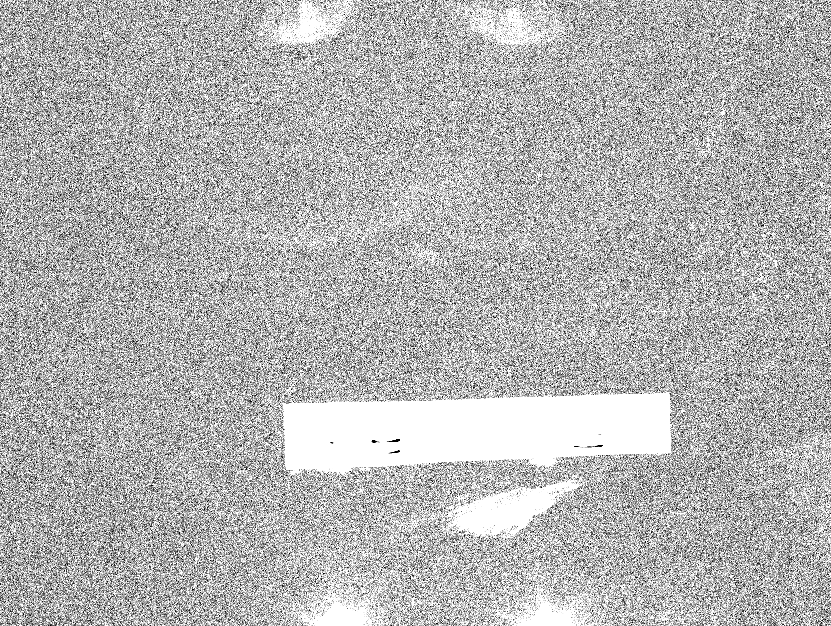

Supplement: Supplementary file 1 [file vetsci-13-00526-s001.zip › Supplementary file S1/Original Images wb/My-3mk/contrast/contrast_8.png]

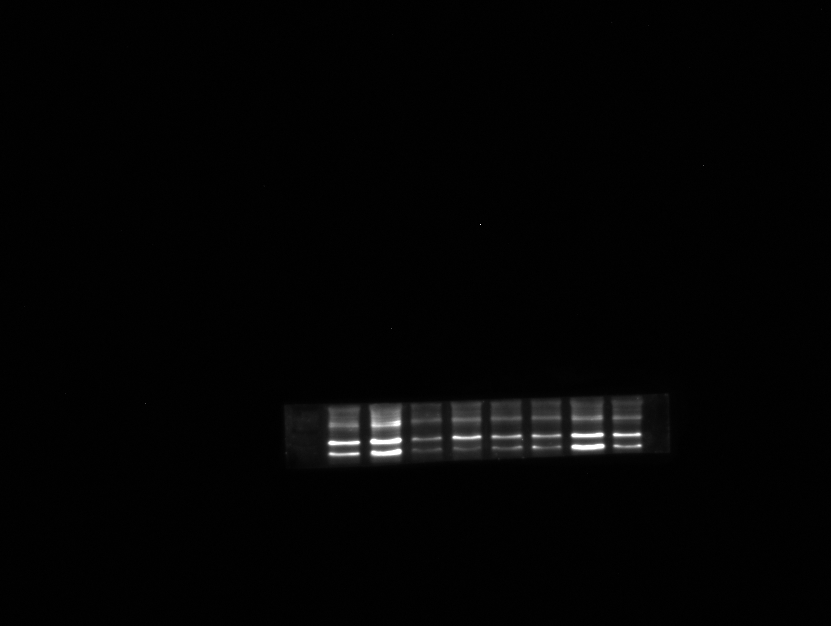

Supplement: Supplementary file 1 [file vetsci-13-00526-s001.zip › Supplementary file S1/Original Images wb/My-3mk/My-3mk2023-12-08_14-59-32_1_16bit.png]

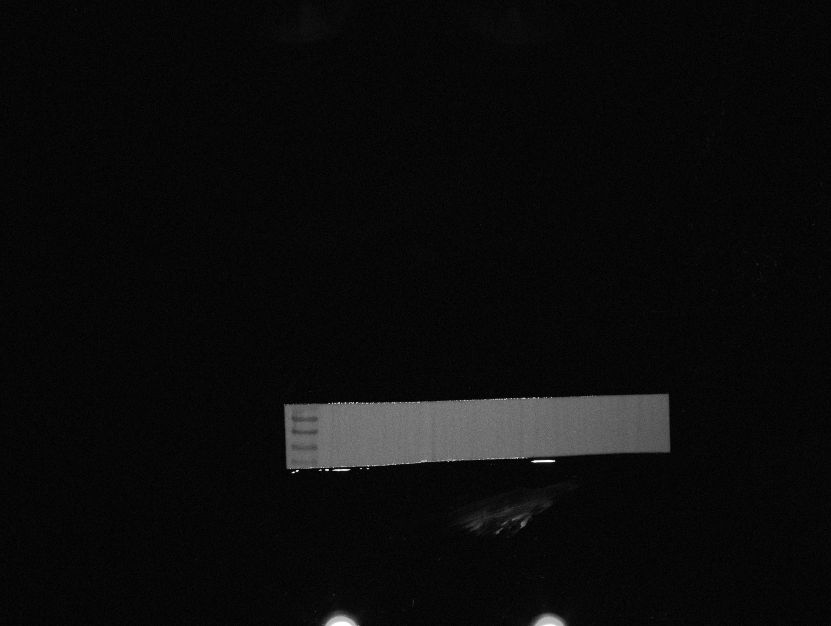

Supplement: Supplementary file 1 [file vetsci-13-00526-s001.zip › Supplementary file S1/Original Images wb/My-3mk/My-3mk2023-12-08_14-59-32_2_16bit.png]

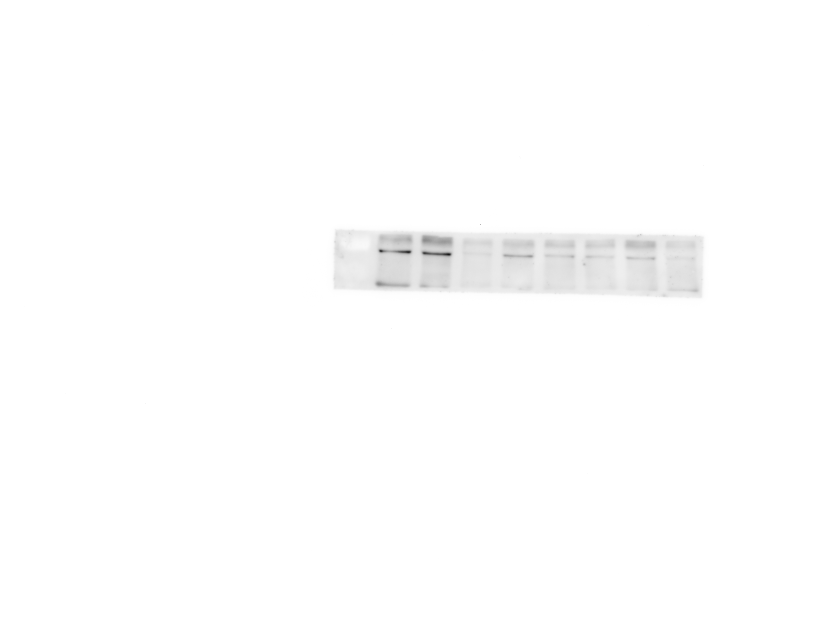

Supplement: Supplementary file 1 [file vetsci-13-00526-s001.zip › Supplementary file S1/Original Images wb/NfκB-2/contrast/contrast_0.png]

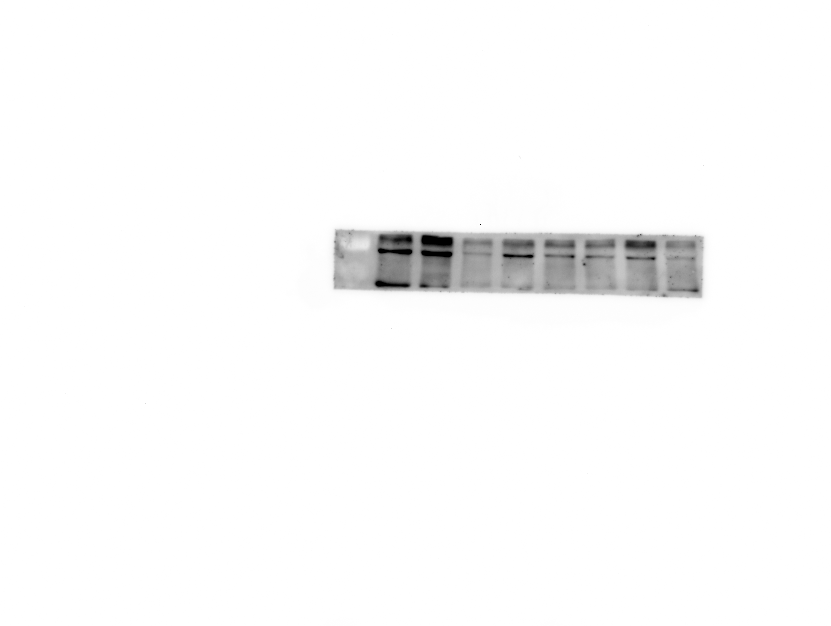

Supplement: Supplementary file 1 [file vetsci-13-00526-s001.zip › Supplementary file S1/Original Images wb/NfκB-2/contrast/contrast_1.png]

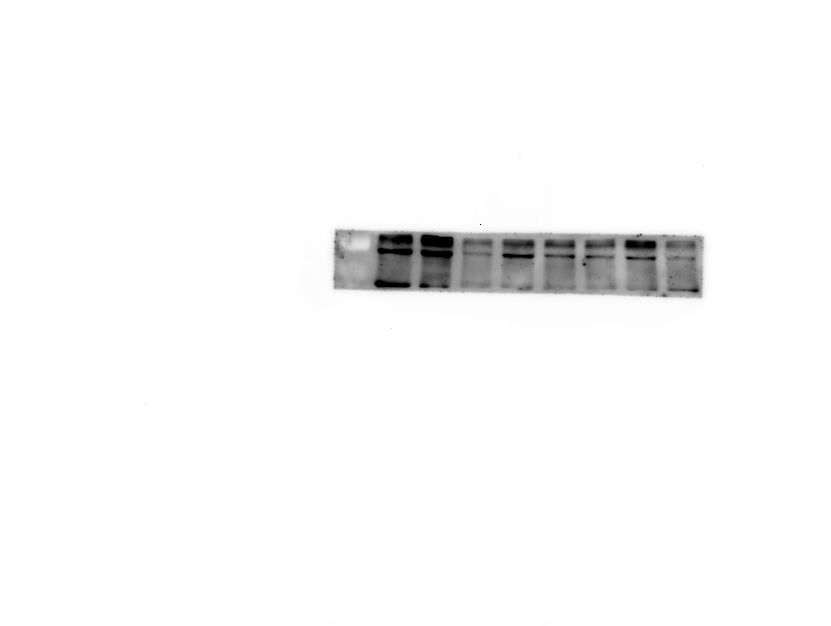

Supplement: Supplementary file 1 [file vetsci-13-00526-s001.zip › Supplementary file S1/Original Images wb/NfκB-2/contrast/contrast_2.png]

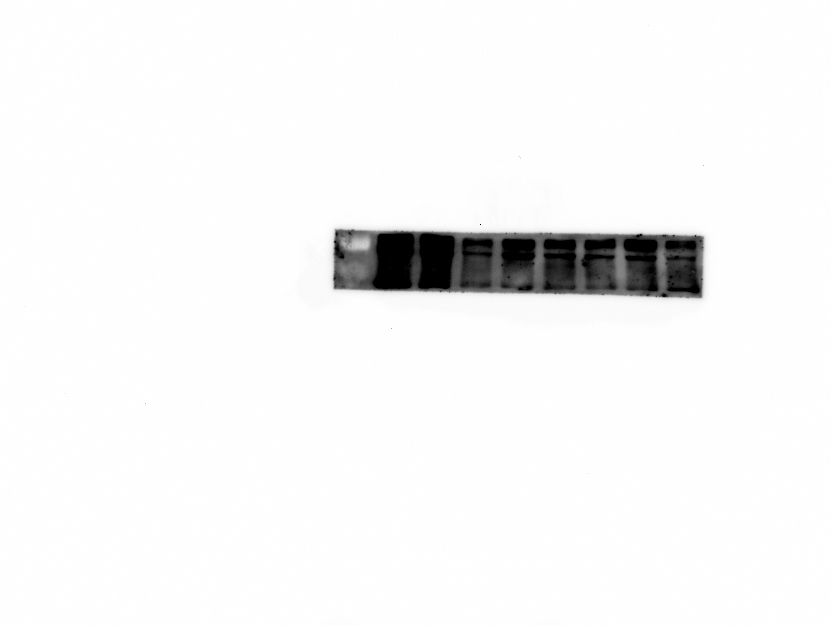

Supplement: Supplementary file 1 [file vetsci-13-00526-s001.zip › Supplementary file S1/Original Images wb/NfκB-2/contrast/contrast_3.png]

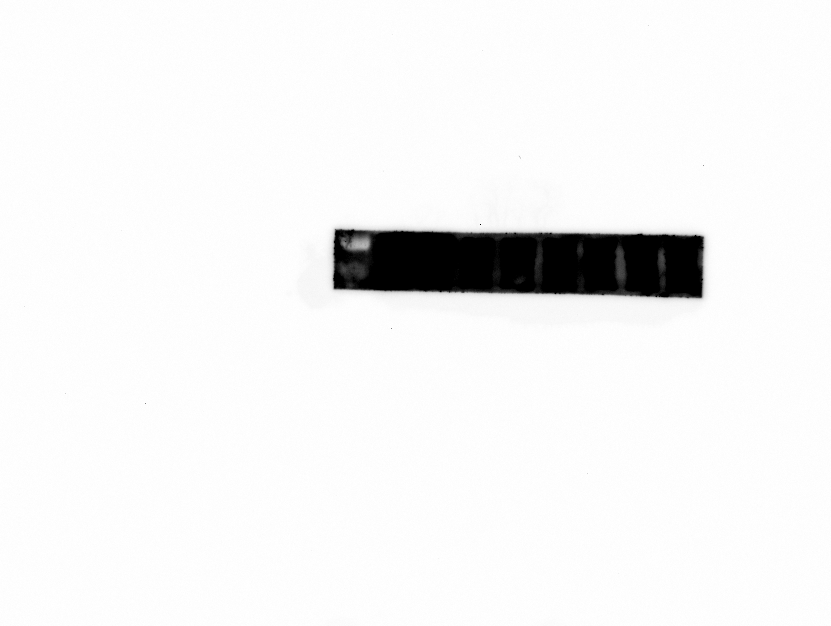

Supplement: Supplementary file 1 [file vetsci-13-00526-s001.zip › Supplementary file S1/Original Images wb/NfκB-2/contrast/contrast_4.png]

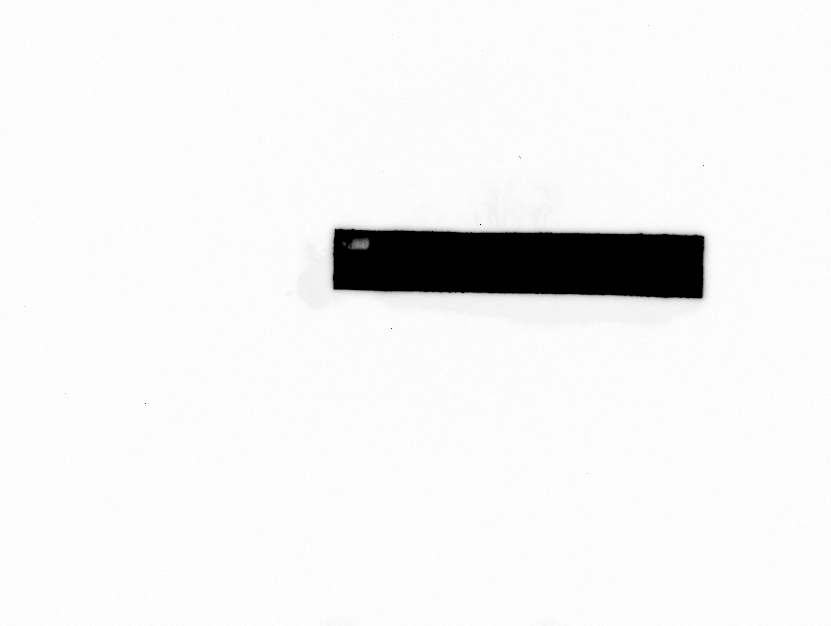

Supplement: Supplementary file 1 [file vetsci-13-00526-s001.zip › Supplementary file S1/Original Images wb/NfκB-2/contrast/contrast_5.png]

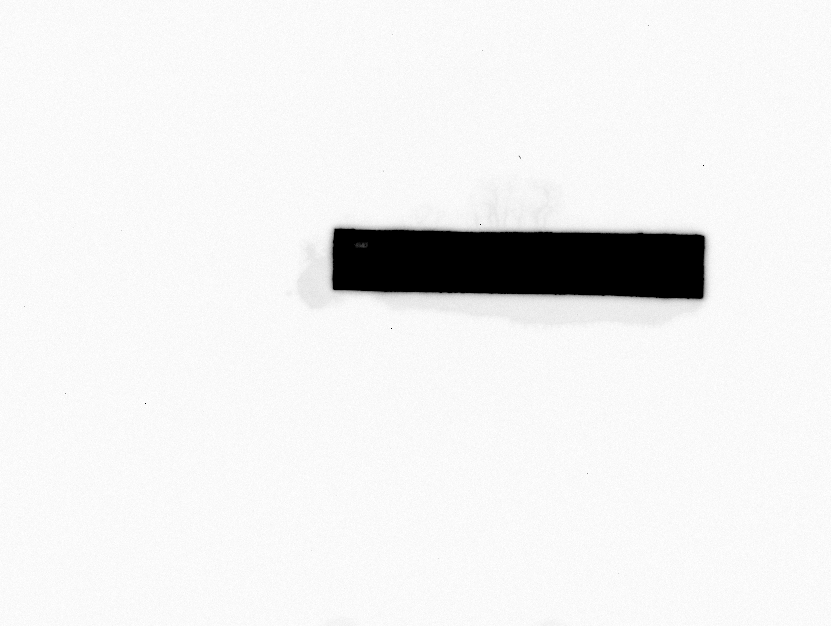

Supplement: Supplementary file 1 [file vetsci-13-00526-s001.zip › Supplementary file S1/Original Images wb/NfκB-2/contrast/contrast_6.png]

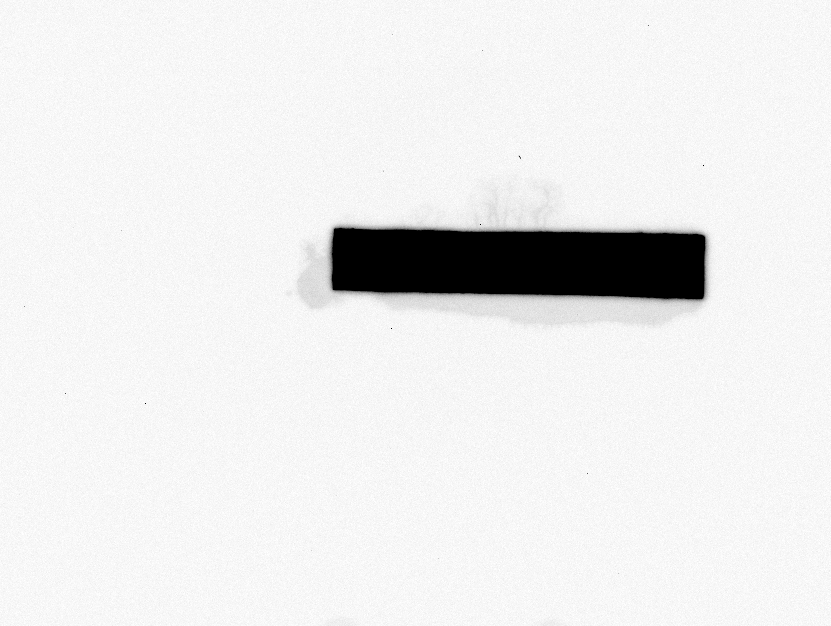

Supplement: Supplementary file 1 [file vetsci-13-00526-s001.zip › Supplementary file S1/Original Images wb/NfκB-2/contrast/contrast_7.png]

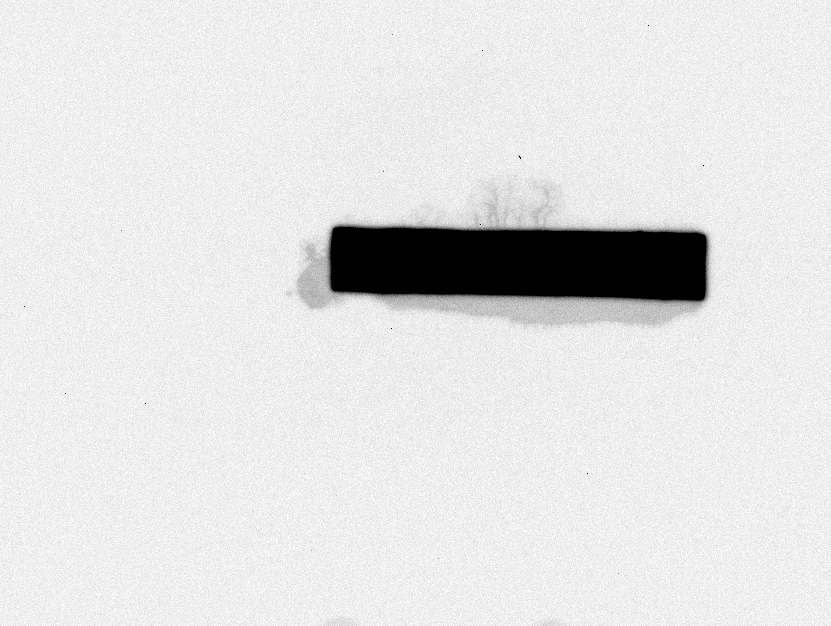

Supplement: Supplementary file 1 [file vetsci-13-00526-s001.zip › Supplementary file S1/Original Images wb/NfκB-2/contrast/contrast_8.png]

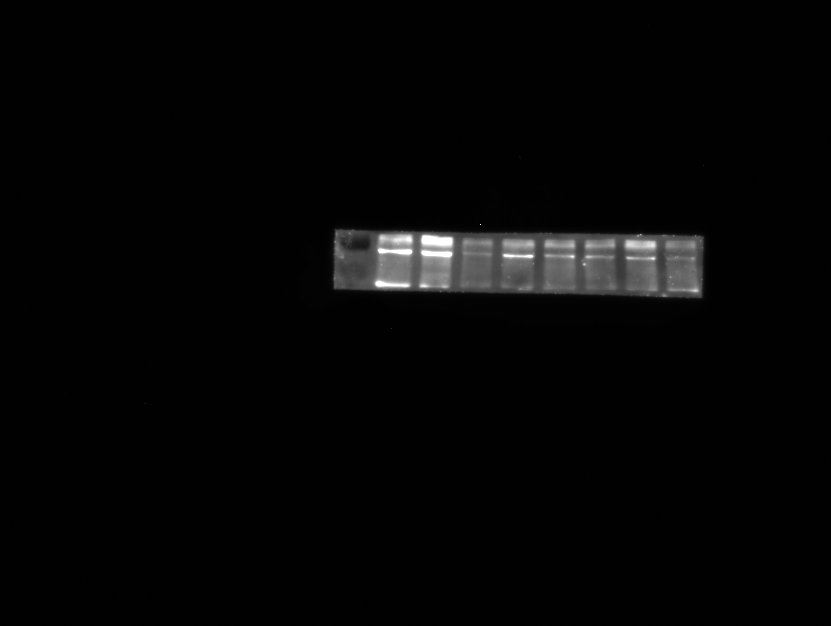

Supplement: Supplementary file 1 [file vetsci-13-00526-s001.zip › Supplementary file S1/Original Images wb/NfκB-2/Nf-2-2023-11-16_10-52-50_1_16bit.png]

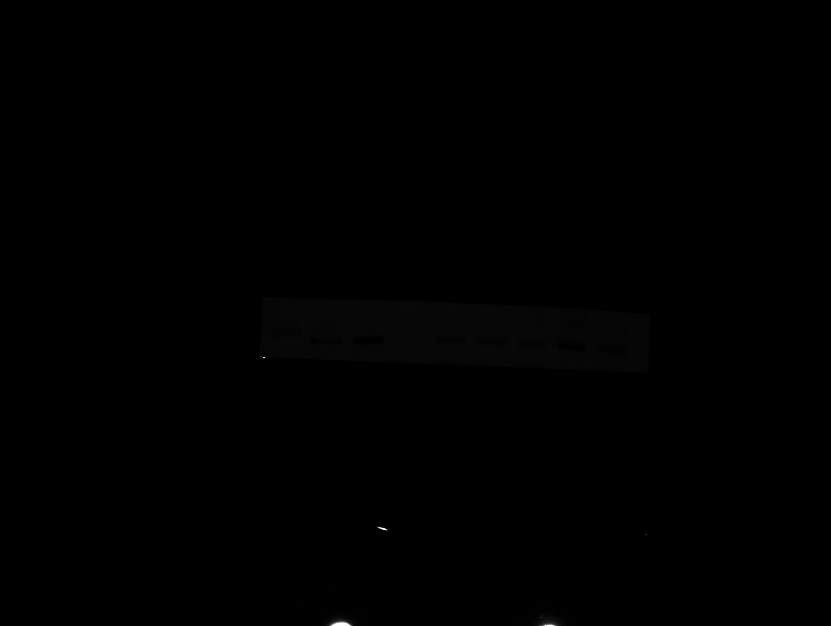

Supplement: Supplementary file 1 [file vetsci-13-00526-s001.zip › Supplementary file S1/Original Images wb/Tlr4-2mk/contrast/contrast_0.png]

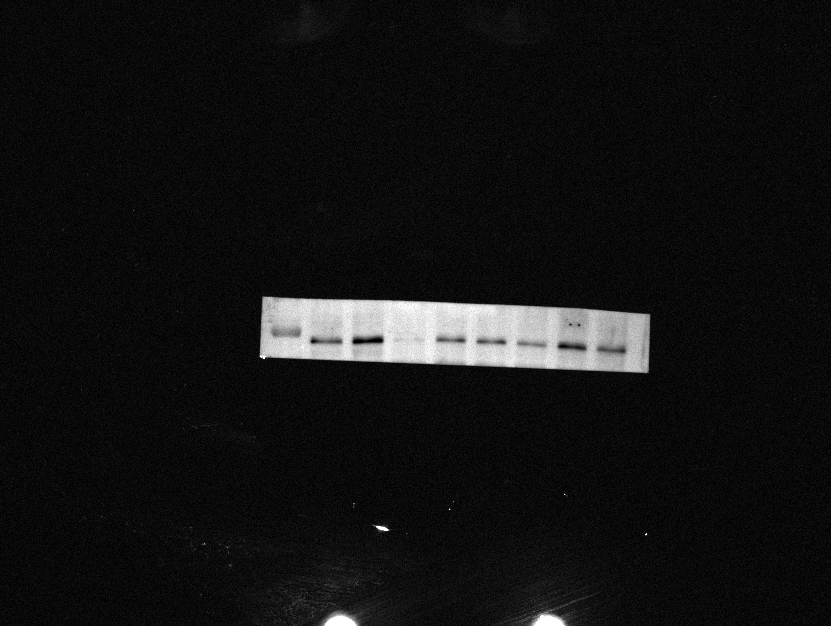

Supplement: Supplementary file 1 [file vetsci-13-00526-s001.zip › Supplementary file S1/Original Images wb/Tlr4-2mk/contrast/contrast_1.png]

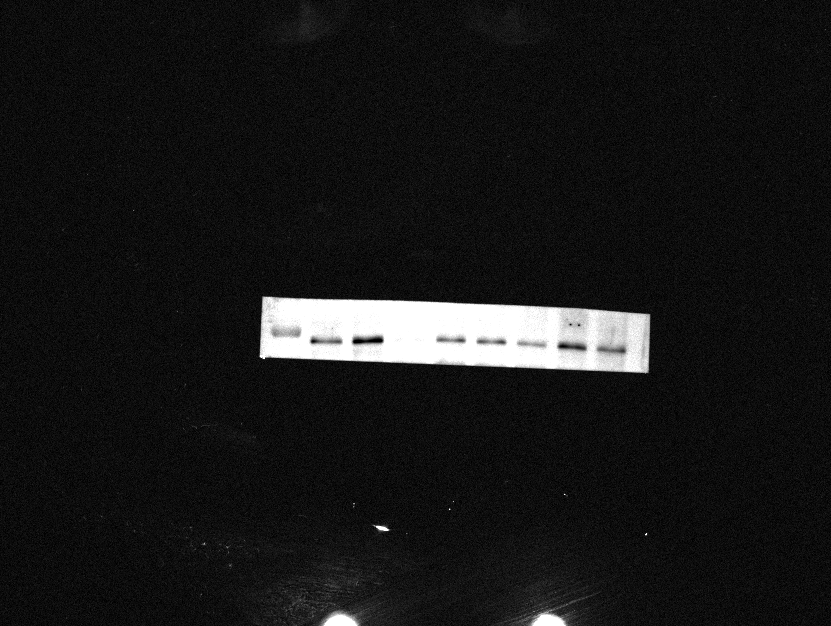

Supplement: Supplementary file 1 [file vetsci-13-00526-s001.zip › Supplementary file S1/Original Images wb/Tlr4-2mk/contrast/contrast_2.png]

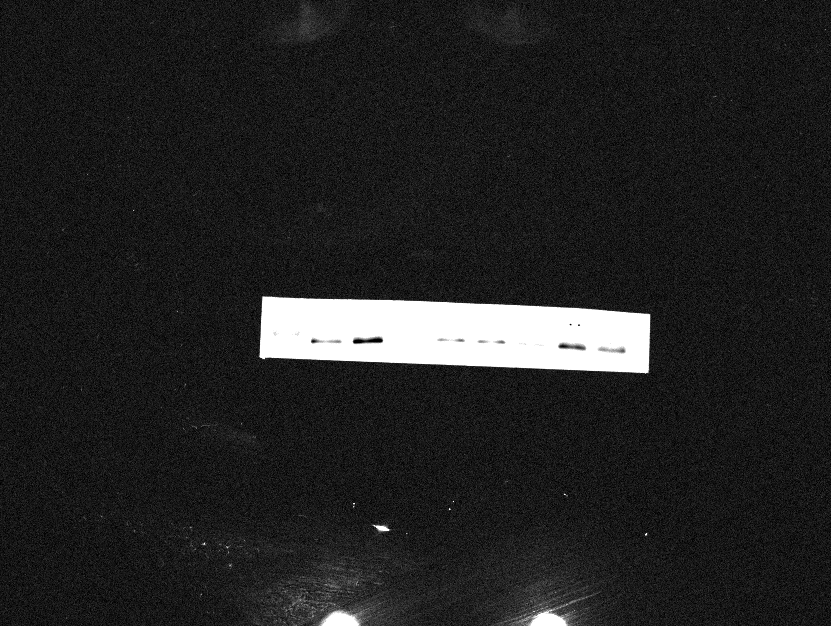

Supplement: Supplementary file 1 [file vetsci-13-00526-s001.zip › Supplementary file S1/Original Images wb/Tlr4-2mk/contrast/contrast_3.png]

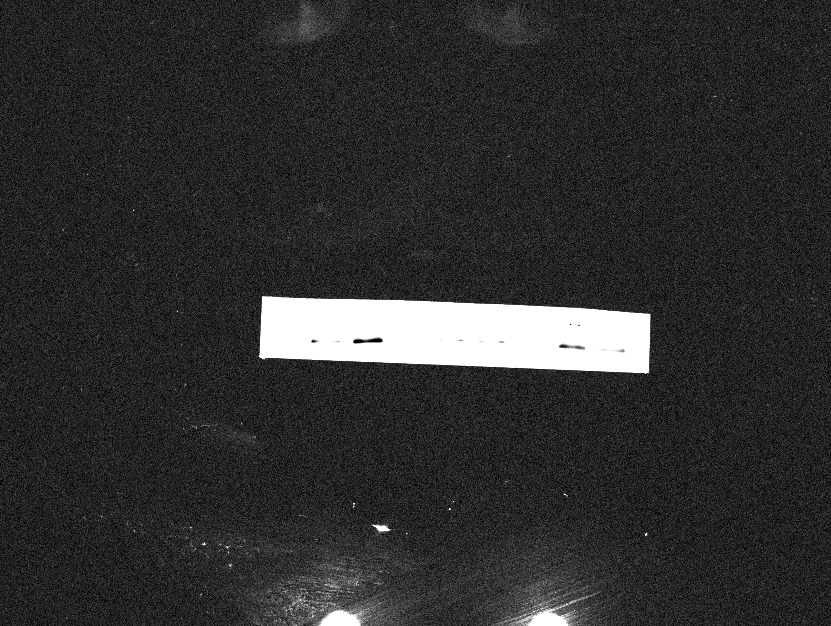

Supplement: Supplementary file 1 [file vetsci-13-00526-s001.zip › Supplementary file S1/Original Images wb/Tlr4-2mk/contrast/contrast_4.png]

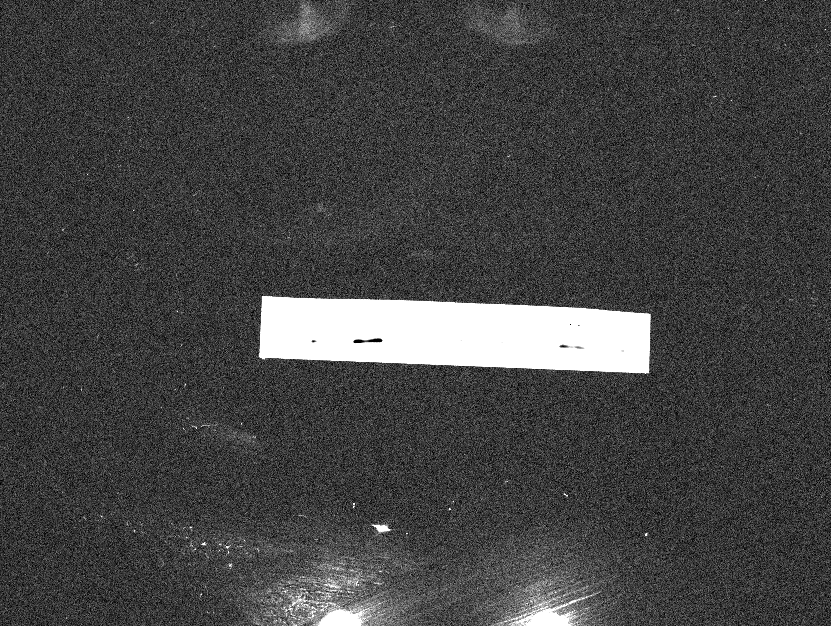

Supplement: Supplementary file 1 [file vetsci-13-00526-s001.zip › Supplementary file S1/Original Images wb/Tlr4-2mk/contrast/contrast_5.png]

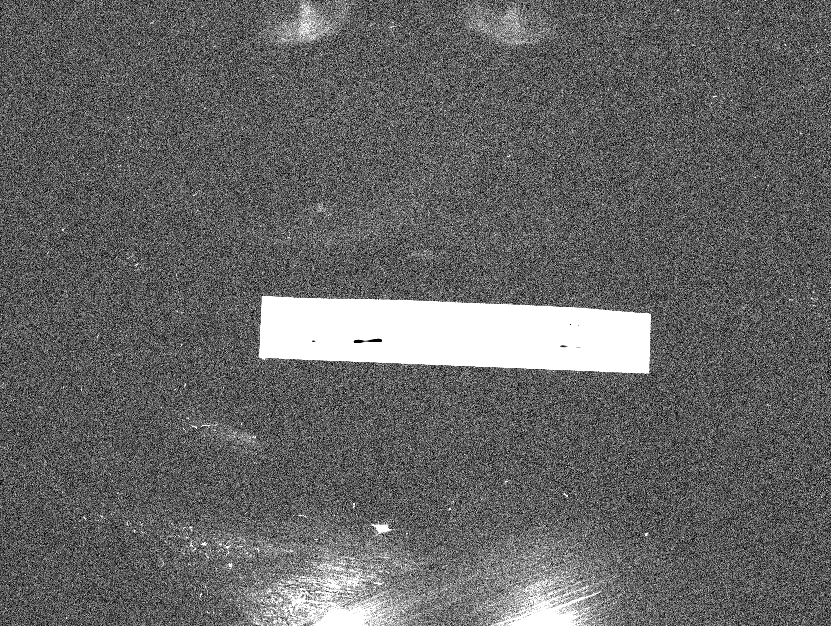

Supplement: Supplementary file 1 [file vetsci-13-00526-s001.zip › Supplementary file S1/Original Images wb/Tlr4-2mk/contrast/contrast_6.png]

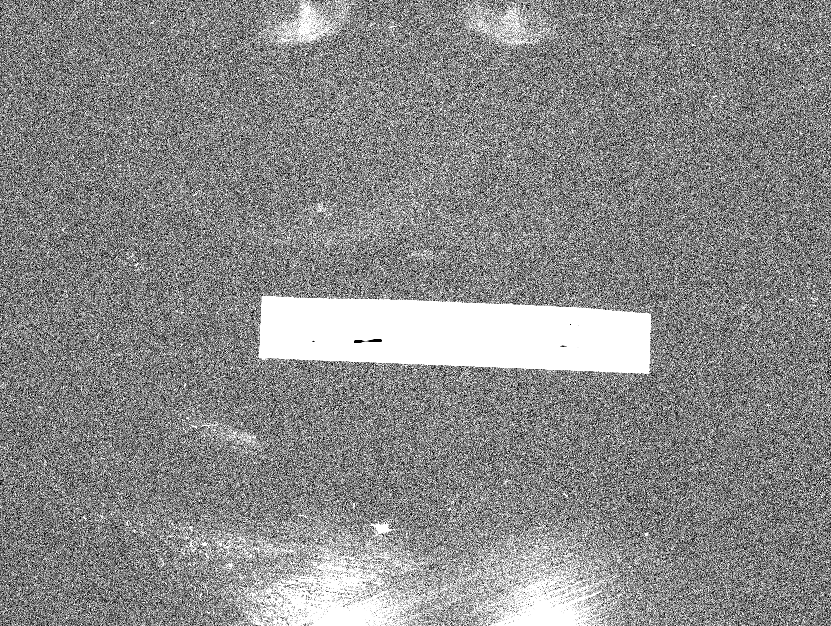

Supplement: Supplementary file 1 [file vetsci-13-00526-s001.zip › Supplementary file S1/Original Images wb/Tlr4-2mk/contrast/contrast_7.png]

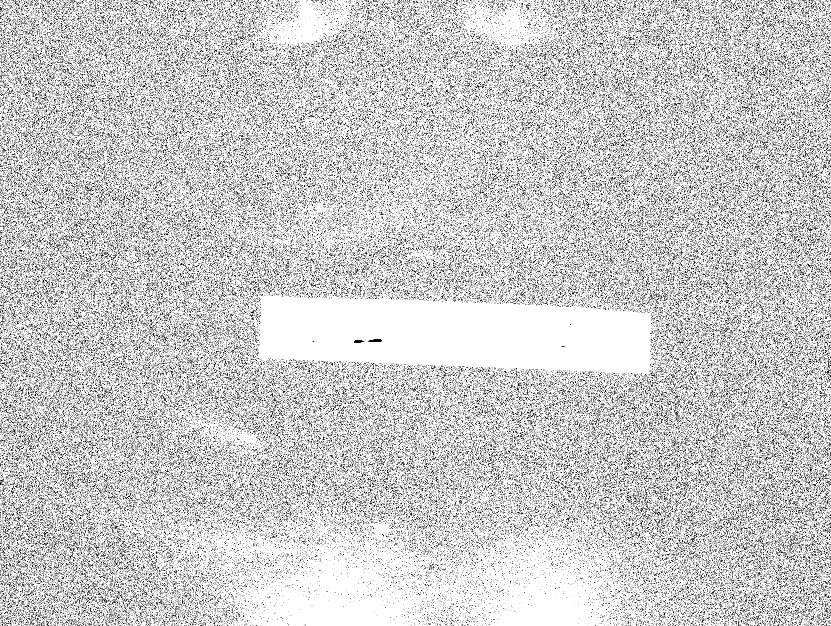

Supplement: Supplementary file 1 [file vetsci-13-00526-s001.zip › Supplementary file S1/Original Images wb/Tlr4-2mk/contrast/contrast_8.png]

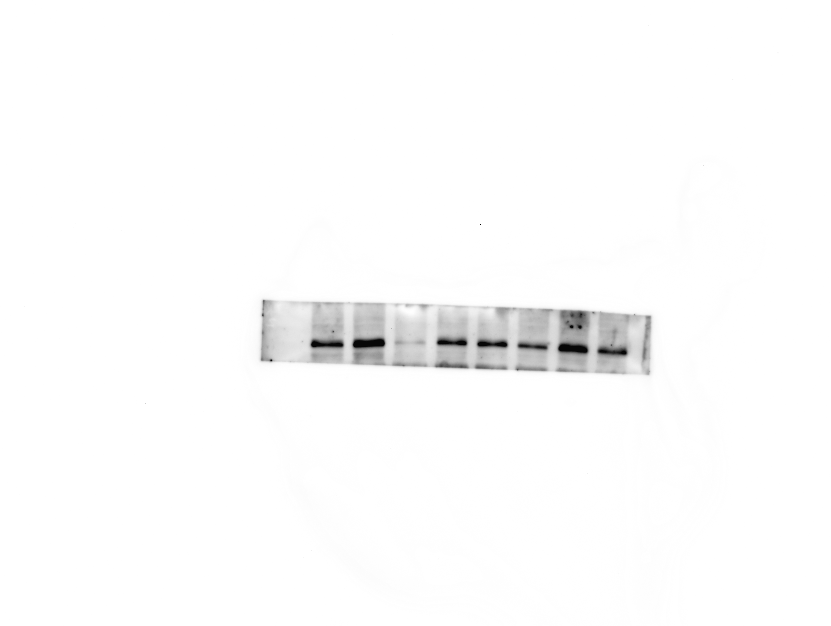

Supplement: Supplementary file 1 [file vetsci-13-00526-s001.zip › Supplementary file S1/Original Images wb/Tlr4-2mk/contrast_1.tif]

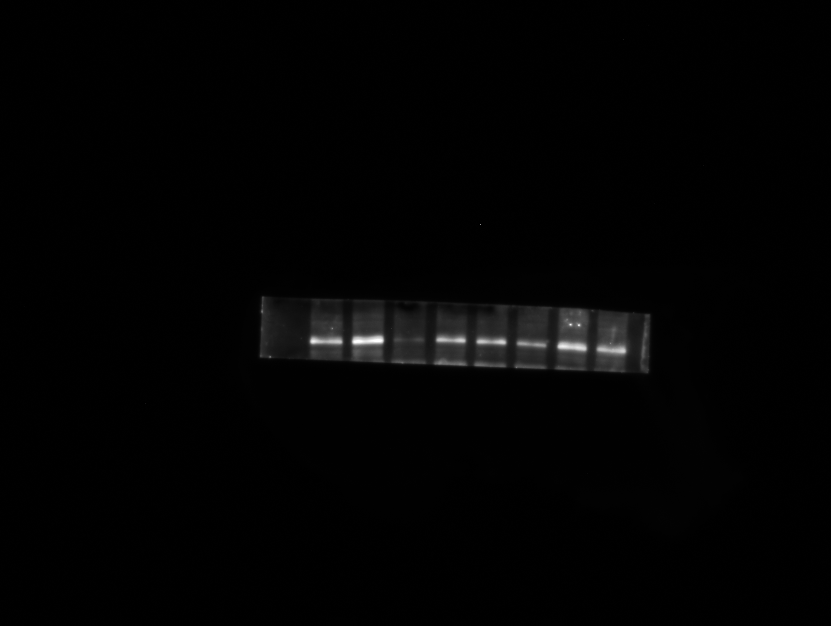

Supplement: Supplementary file 1 [file vetsci-13-00526-s001.zip › Supplementary file S1/Original Images wb/Tlr4-2mk/Tlr4-2mk2023-10-30_13-43-29_1_16bit.png]

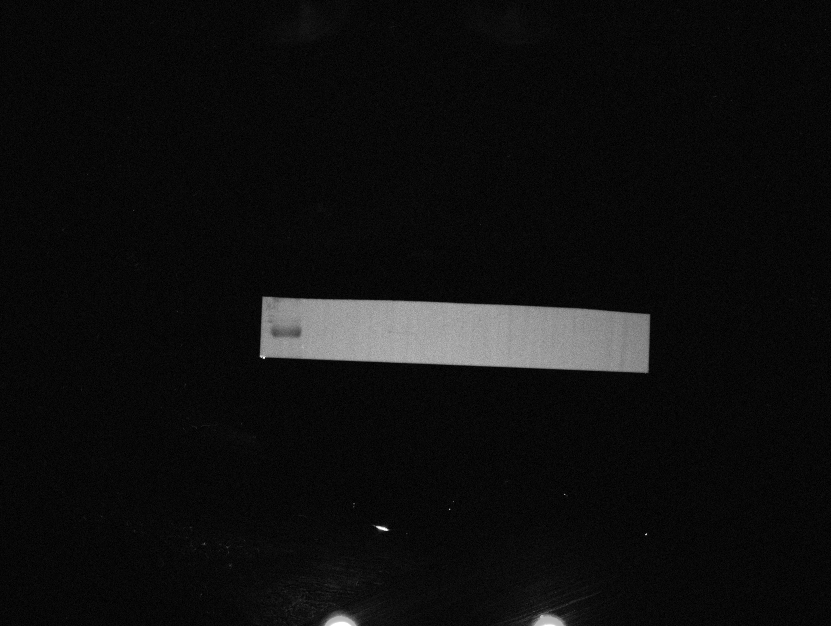

Supplement: Supplementary file 1 [file vetsci-13-00526-s001.zip › Supplementary file S1/Original Images wb/Tlr4-2mk/Tlr4-2mk2023-10-30_13-43-29_2_16bit.png]

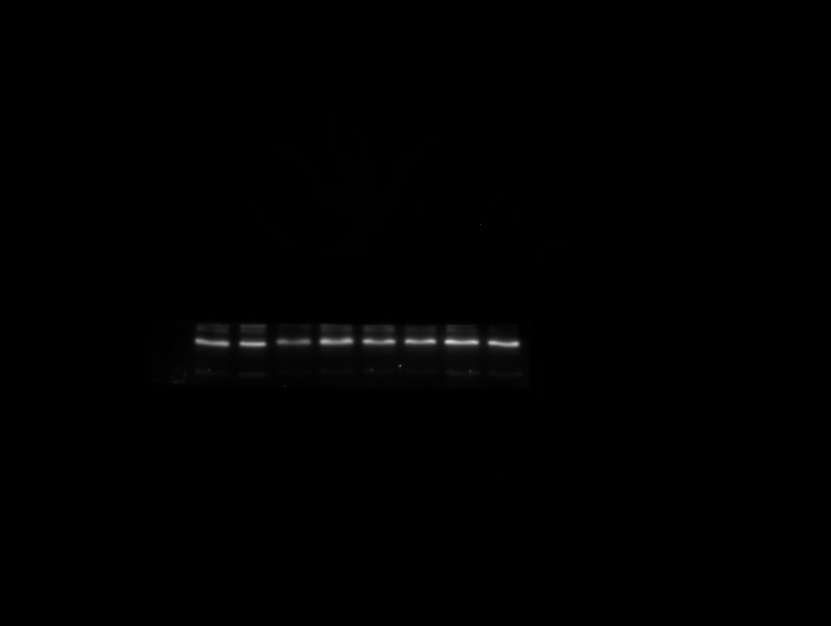

Supplement: Supplementary file 1 [file vetsci-13-00526-s001.zip › Supplementary file S1/Original Images wb/TNFα -2/contrast/contrast_0.png]

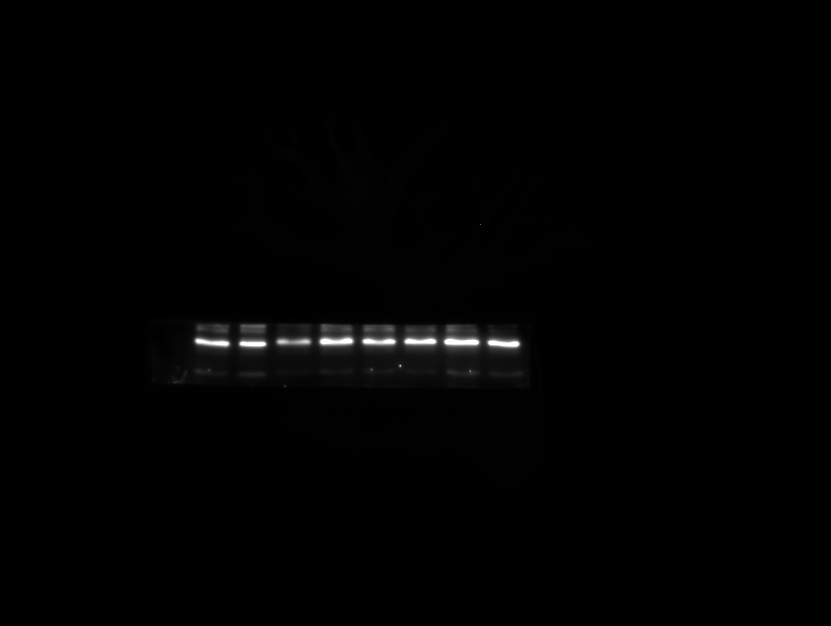

Supplement: Supplementary file 1 [file vetsci-13-00526-s001.zip › Supplementary file S1/Original Images wb/TNFα -2/contrast/contrast_1.png]

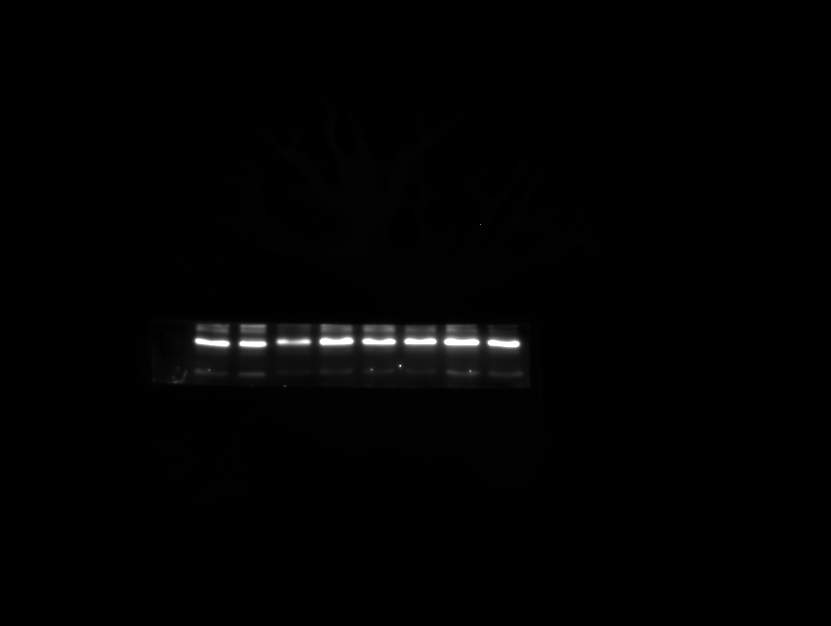

Supplement: Supplementary file 1 [file vetsci-13-00526-s001.zip › Supplementary file S1/Original Images wb/TNFα -2/contrast/contrast_2.png]

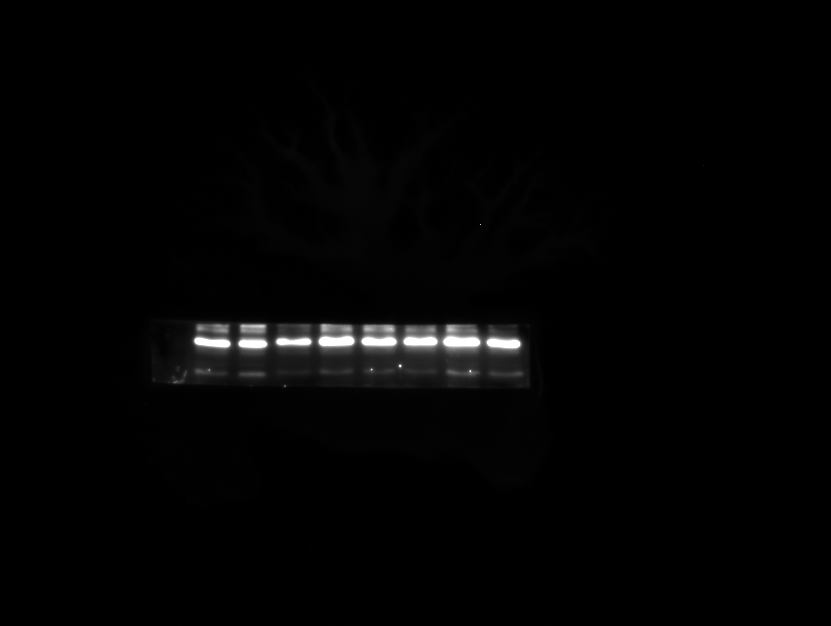

Supplement: Supplementary file 1 [file vetsci-13-00526-s001.zip › Supplementary file S1/Original Images wb/TNFα -2/contrast/contrast_3.png]

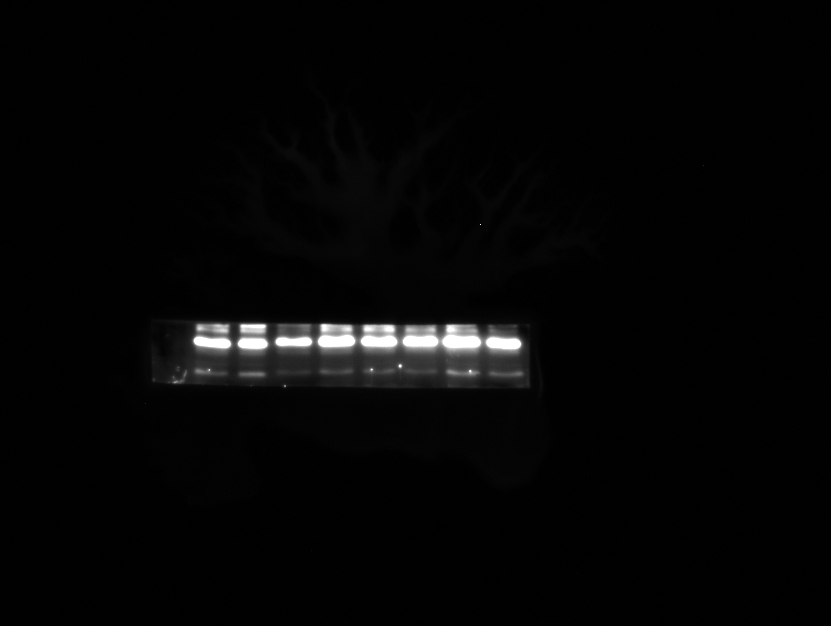

Supplement: Supplementary file 1 [file vetsci-13-00526-s001.zip › Supplementary file S1/Original Images wb/TNFα -2/contrast/contrast_4.png]

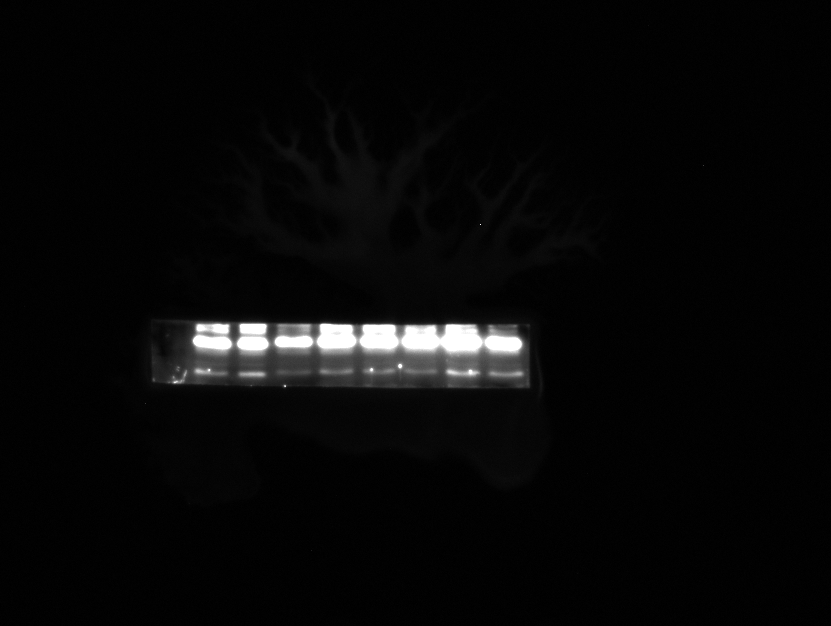

Supplement: Supplementary file 1 [file vetsci-13-00526-s001.zip › Supplementary file S1/Original Images wb/TNFα -2/contrast/contrast_5.png]
